# Emergent heterogeneity in putative mesenchymal stem cell colonies: Single-cell time lapsed analysis

**DOI:** 10.1371/journal.pone.0213452

**Published:** 2019-04-03

**Authors:** Deena A. Rennerfeldt, Joana S. Raminhos, Samantha M. Leff, Pristinavae Manning, Krystyn J. Van Vliet

**Affiliations:** 1 Department of Biological Engineering, Massachusetts Institute of Technology, Cambridge, Massachusetts, United States of America; 2 Department of Materials Science, Faculty of Science and Technology, New University of Lisbon, Caparica, Portugal; 3 Department of Materials Science and Engineering, Massachusetts Institute of Technology, Cambridge, Massachusetts, United States of America; Pennsylvania State Hershey College of Medicine, UNITED STATES

## Abstract

Bone marrow stromal cells (BMSCs) include a subset of stem cells that are considered promising for developmental studies and therapeutic applications. While it is appreciated generally that BMSC populations can exhibit morphological and functional heterogeneity upon *in vitro* culture expansion, the potential for heterogeneity within a single colony forming unit–generated ostensibly from a single mother cell–is less explored but is critical to design of both fundamental studies and cell therapy production. Here we observed BMSC colony formation in real time via time lapsed optical imaging and analysis, to quantify whether and how heterogeneity emerged over multiple cell divisions spanning the duration of a typical colony formation unit assay. These analyses demonstrate that such colonies are neither homogeneous subpopulations of stem cells nor necessarily derived from single originating cells. While the mechanisms for and causes of this intracolony heterogeneity are not understood fully, we further demonstrate that extensive cell-cell contacts do not correlate with senescence, but that media exchange was concurrent with diversification in even the most uniform single-cell-derived colonies. These direct quantitative observations and visualizations of colony formation provide new insights that are motivated by significant implications for both basic research and stem cell-based therapies.

## Introduction

Bone marrow stromal cells (BMSCs), a subset of which are characterized as multipotent mesenchymal stem cells, are considered potential cell therapies for diverse medical conditions–from bone tissue regeneration to autism and Parkinson’s disease [1–3]. Such culture-expanded BMSC populations have been implemented in over 600 clinical trials since 1995 (http://www.clinicaltrials.gov). Despite these advances at the lab scale and in ongoing clinical trials, however, we are aware of no therapies to date that have been approved by the U.S. Food & Drug Administration for delivery of BMSCs or putative mesenchymal stem cells (http://www.clinicaltrials.gov).

It is discussed broadly that one contributing factor of unsuccessful clinical trials is variability among individual participants. In the case of BMSC autologous therapies, this variability of outcome can be attributed to both variability among patients receiving treatment and to the variability among donors of the initial bone marrow aspirates and the ensuing *in vitro*-expanded BMSC populations derived thereof [4–6]. However, even for cells obtained from a single donor, variation is manifest in the functional heterogeneity–including variable proliferation rates, number of colony forming units, and differentiation potential–that emerges within *in vitro*-expanded BMSC populations that are cultured under ostensibly identical culture protocols [5,7–9]. For at least some clinical endpoints, including bone marrow regeneration, it has been shown that functional heterogeneity among the administered BMSCs can lead to substantially diminished therapeutic outcomes [10]. Nevertheless, heterogeneity onset in stem cell cultures–while acknowledged anecdotally–is often overlooked and underappreciated in many studies [11,12]. Further, there is no standardized protocol to maintain or quantify critical quality attributes of culture-expanded BMSCs indicative of safety and efficacy for clinical trials beyond pre-expansion screening of the surface marker panels suggested by the International Society for Cellular Therapy [13], and completed clinical trials typically do not publish their methods and findings in peer-reviewed journals [14,15].

To avoid the complications inherent in studying heterogeneous cell populations, many studies focusing on basic BMSC biology or therapies have used so-called “clonal” BMSCs. These cells are those within a BMSC population that belonged to or were expanded from a single colony. As such, it is tacitly assumed by most that the colony was derived from a single cell and that cells within the resulting colony were functionally homogeneous stem cells. Such assumptions, which are also implicit in many studies of other cell classes, are sufficiently pervasive that many consider colony formation as a defining criterion for the putative stem cells within a BMSC population. However, a competing consideration is the demonstration that even cells within a BMSC colony are heterogeneous in terms of gene and surface marker expression and morphology (see Rennerfeldt and Van Vliet [16] for review). Here, we present our findings from time lapse image analysis of multiple colonies grown under a standard colony-forming-unit assay culture protocol. We demonstrate variability among colonies generated from cells obtained from the same donor and expanded in the same vessel, and we present a single-cell-analysis of dozens of biophysical and biological properties to report the extent of single-cell-level heterogeneity within individual colonies. Our findings suggest that colonies grown in sparsely plated BMSC cultures most typically are neither accurate representatives of their entire putative stem cell subpopulation nor collections of functionally homogeneous stem cells originating from a single rapidly dividing mother cell. We further demonstrate that the onset of senescence within colonies cannot be explained by extensive cell-cell contacts and provide additional evidence demonstrating that common changes in the biochemical environment can instigate deviations in cell behavior. Such findings hold implications for, and suggest future work required to enable, both basic research on stem cell biology and applications of culture expanded BMSCs for therapeutic applications.

## Materials and methods

Detailed methods are located following the References, for manuscript readability. These methods include: cell source, expansion, and serial dilution to attain sparsely seeded dishes for colony imaging; time-lapse image acquisition of colony growth; image analysis including extensive image preprocessing to identify and track and distinguish cells over days of continuous culture and imaging, parameter extraction and classification; construction of multivariate lineage trees; correlation analysis linking parameters to intracolony heterogeneity; processes for creating data visualizations; and statistical analysis to identify differences among and within colonies. We have also provided a detailed experimental protocol at protocols.io [17] and source code for image processing and analysis as a GitHub repository [18].

## Results

### Intercolony heterogeneity can result from multiple progenies within a putative clone

**Fig 1:**
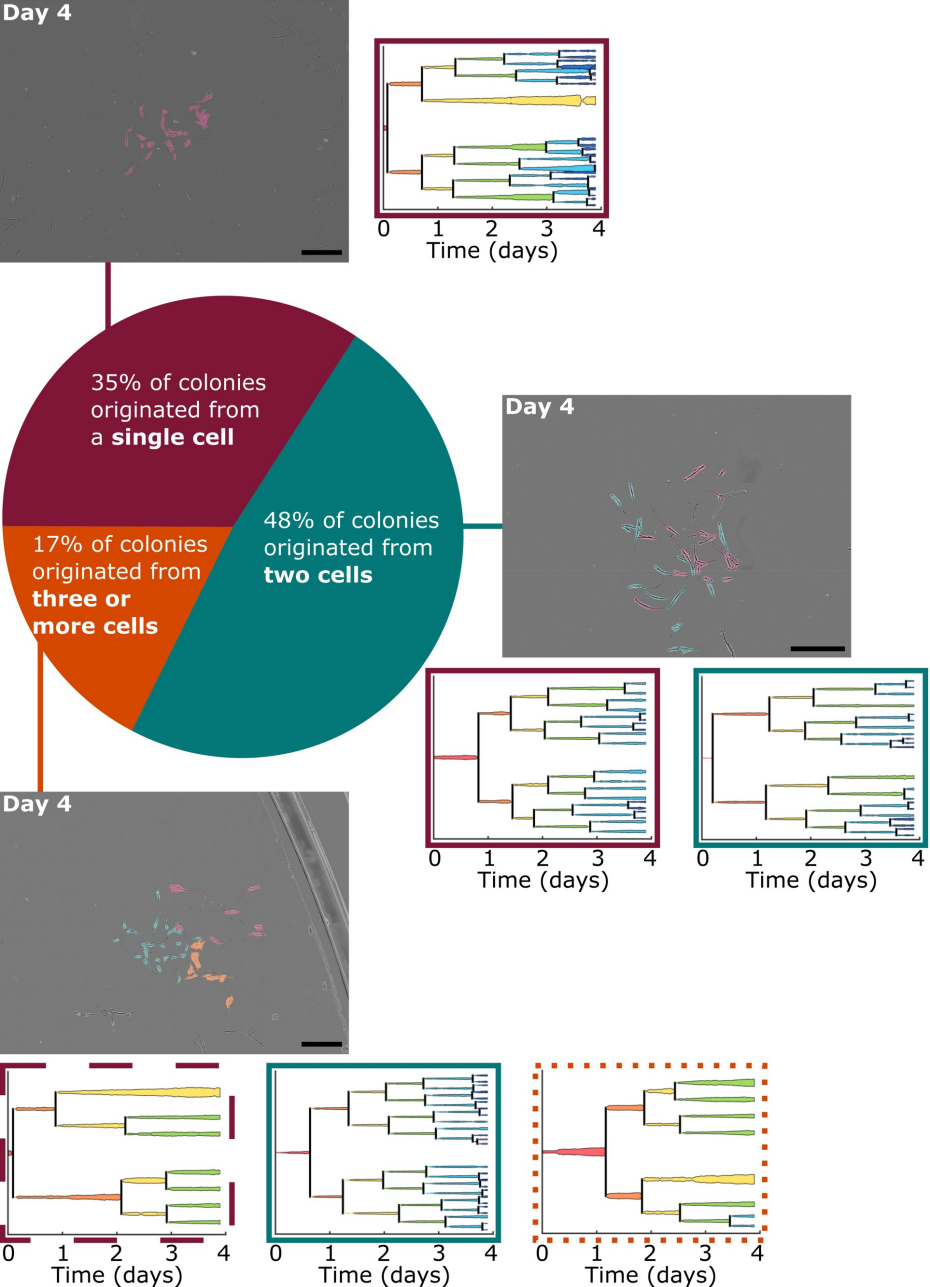
Colonies observed after four days of growth varied by the number of originating progenies. Of all locations in which colony formation was observed, approximately 35% originated from a single tracked cell, 48% originated from two analyzed cells, and the remainder formed from three or four originating cells that attached near each other at the start of the experiment. Phase contrast images of three representative colonies after four days of growth are displayed, organized by the number of analyzed cells the colonies originated from. Images are montages of nine or sixteen fields of view and were altered post-experiment to artificially color the BMSCs by the progeny they belong to. Cells without color added are BMSCs that migrated into the montaged field of view after initial imaging. Lineage trees of the colonies’ originating cells are also presented, where the width of the lineage lines is representative of the respective cell spread area at each 15 min time point. *Lineage trees and images of all colonies at several time points are presented in S1 Fig*. Lineage tree outlines indicate the proliferative capacity of the progeny. Dashed line (- -): progeny classified as slow proliferator; dotted line (…): progeny classified as moderate proliferator; solid line (–): progeny classified as fast proliferator. Scale bars = 0.5 mm.

It is well documented that BMSC colonies derived from a given donor (i.e., single biological cell source) vary substantially from each other in terms of colony size, confluency, and multilineage potential [5,19–23]. Unfortunately, the vast majority of reported studies involving colony formation have not demonstrated rigorously that each colony included solely the progeny of a single originating cell [16]. To understand the prevalence of single cell origin under typical colony-forming conditions, we plated human BMSCs at low density (approximately 30 cells/cm^2^; see Methods) and imaged at sufficiently high resolution to resolve individual cells (1344 x 1024 pixels per field of view, Fig 1) every 15 min over the course of 14 days. Following conventional BMSC culture protocols, cells were fed twice per week, beginning at the end of Day 3 of the experiment. We processed and analyzed these images at the single-cell level for the first four days of growth for 28 colonies, and we further analyzed colony-wide properties at Day 7 up to Day 10 to ensure full colony formation, defined herein as a group of at least 50 cells. This time lapse imaging approach enabled us to confirm definitively the potential for apparent colonies to include progeny of multiple originating cells.

We termed colonies originating from a single isolated cell (i.e., only analyzable cell in the viewing frame at the onset of the experiment) as single-cell-derived (SCD) colonies and colonies that originated from more than one cell as multi-cell-derived (MCD) colonies. We observed that nearly half (48%) of colonies analyzed at the single-cell level through Day 4 originated from two BMSCs, while a lower fraction (35%) originated from single cells (Fig 1). For each of the studied colonies, S1 Fig includes lineage trees up to Day 4 and phase contrast images at Days 4, 7, and 10. Importantly, within seven days of colony growth, all SCD colonies contained at least one cell that had migrated toward and infiltrated the originating progeny (S1 Fig, Colonies A, D, E, F, L, P, Q2, T, and Z). Time lapse imaging also enabled us to conclude that some units which would be designated as a colony at later time points (e.g., Day 7 or 10) were actually multiple colonies that joined to form a larger MCD colony (S1 Fig, colonies Y and F), or a collection of several adjacent progenies of varying proliferative capacity (S1 Fig, Colonies C, H, I, and M). Growth of one SCD and one MCD colony, respectively, is illustrated additionally in S8 and S9 Figs.

We also categorized the progenies comprising each colony in terms of proliferative capacity, in order to understand the proliferation kinetics needed to form a colony in sparsely-seeded conditions. We classified originating cells that yielded more than 16 cells (four population doublings) at Day 4 as “fast proliferators,” and progenies that produced fewer than 8 cells (three population doublings) as “slow proliferators.” The remaining progeny–any progeny containing 8 to 16 cells at the Day 4 time point–were classified as “moderate proliferators.” Of the 52 progenies contributing to colony formation, 28% were classified as fast proliferators, 50% as moderate proliferators, and 22% as slow proliferators. Several combinations of slow, moderate, and fast-dividing progeny contributed to colony formation (S1 Fig). Among the SCD colonies, only 33% were formed by fast proliferators (S1 Fig, Colonies A, D, and T), and the remaining 67% were formed by moderate proliferators. Additionally, 39% of MCD colonies contained at least one slowly proliferating progeny.

**Fig 2:**
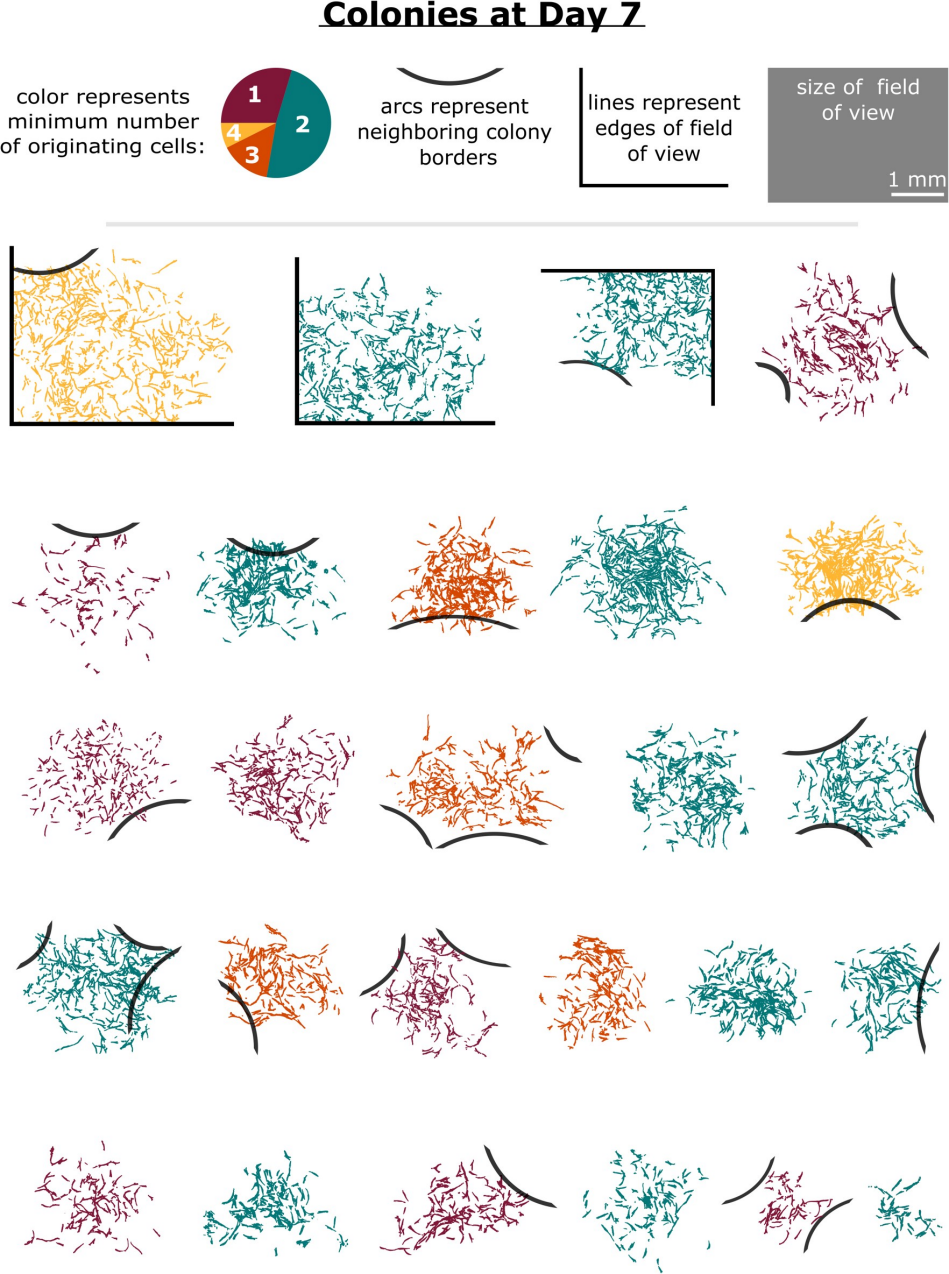
Colonies varied by size, confluency, number of originating progenies, and degree of isolation after seven days of growth. Binarized and recolored phase contrast images of all analyzed colonies after seven days of growth. The color of each imaged colony was altered to represent the number of originating cells associated with each colony, as analyzed by time lapse image analysis at the single-cell level up to Day 4. Borders of neighboring colonies that were either touching or overlapping the colonies studied are represented as black arcs and were traced based on the original phase contrast images. The size of the 16 montaged fields of view at Day 7 is also provided for reference. No statistical correlation was found between the number of cells the colony originated from and colony diameter, confluency, or number of neighboring colonies. (See S2 Fig for further details.) Colonies are ordered by approximate diameter. Unaltered phase contrast images of all colonies are provided in S1 Fig.

We then sought potential correlations with colony properties that did not require detailed time lapse image analysis in an effort to predict multi-cell origin. Fig 2 provides images of the colonies after 7 days of growth and summarizes tests for correlation between the number of colony-originating progenies and more readily observable colony features such as colony diameter, confluency, and number of neighboring colonies. The colonies analyzed at Day 7 exhibited an average confluency of 12.7 +/- 4.9% and an average approximate diameter of 1.9 +/- 0.8 mm (S2 Fig). We compared the above colony-level properties to several other detailed parameters, including the number of fast/moderate/slow-proliferating progeny contributing to the colonies, the number and fraction of senescent cells at Day 4 (defined by the failure to divide by Day 7), and the total and cumulative number of cells belonging to colonies over four days of growth (S1 Table). The properties measurable without time lapse image analysis at Day 7 –colony confluency, diameter, and number of neighboring colonies–did not significantly correlate with the number of originating progeny nor with any of the other properties measured (S1 Table), as determined by the critical value of the Spearman rank correlation coefficient [24]. Further, we noted no statistical significance in these three properties between SCD colonies and MCD colonies when using a Bonferroni-corrected p value of 0.0005 as a threshold for significance (S2D Fig and S1 Table).

### Intracolony heterogeneity: Differences emerge among cells within colonies, even when known to be derived from single cells

While many studies assume that colonies formed in BMSC cultures are functionally uniform (and therefore differences among cells isolated from a given colony are due to other causes, such as environmental cues), there is evidence to suggest that cells within each BMSC colony are functionally heterogeneous [16]. To test the implicit assumption of intracolony similarity with the benefit of our time lapse imaging data, we conducted principal component analysis (PCA) on the z-score normalized dataset to determine how similar cells within a colony were to each other, compared to cells in other colonies. We quantified a total of 54 properties (see “Materials and methods”) relating to the morphology and location of cells, measured for 1384 cells at every 15 min time point of the cells’ existence during the first four days of colony growth. We combined properties relative to each cell’s timeline–namely, each cell’s properties at the time point of its birth, as well as properties averaged over the course of each cell’s lifetime–with additional manually calculated properties (S2 Table) into one dataset for a total of 128 properties.

**Fig 3:**
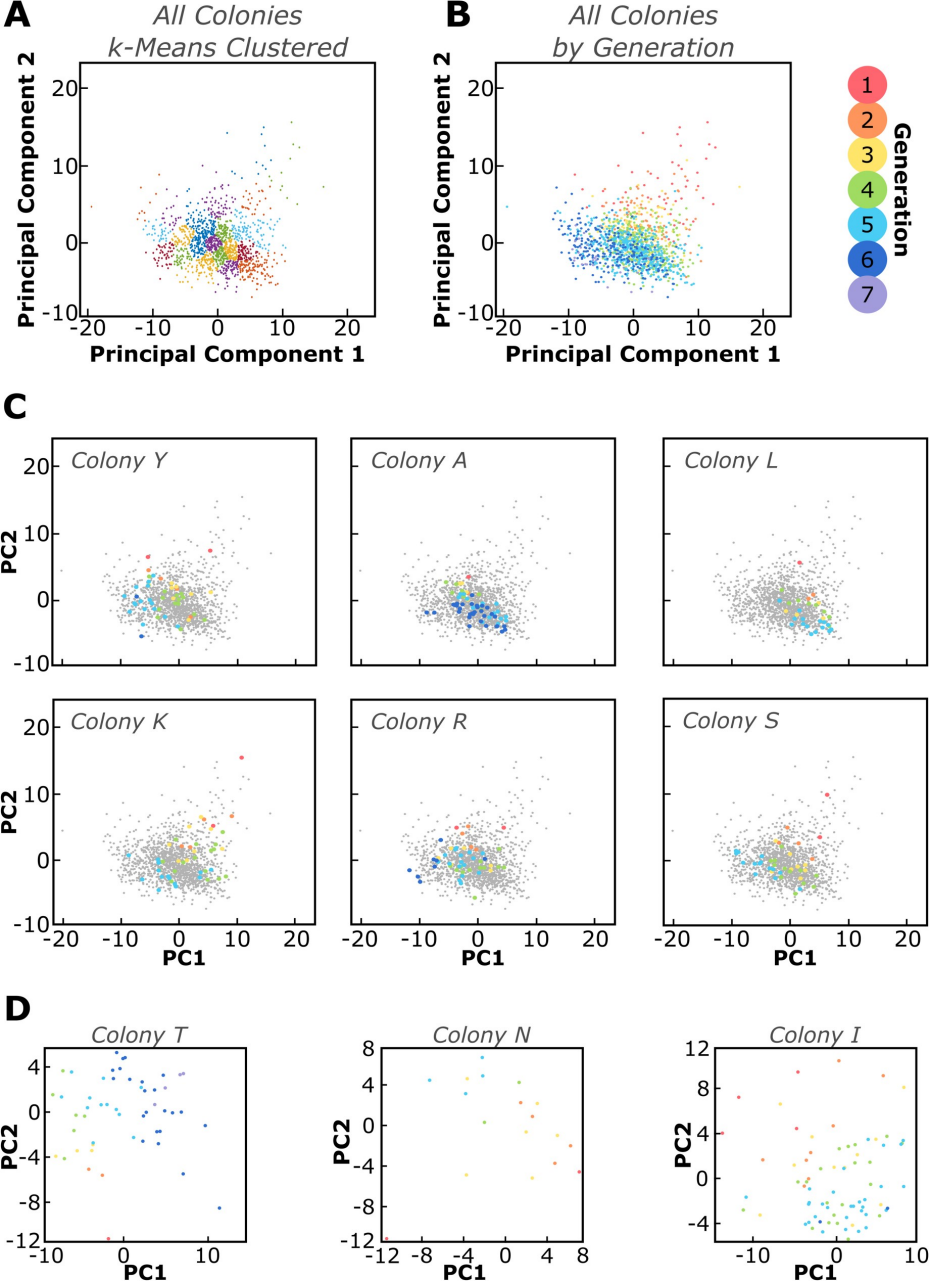
Principal component analysis revealed a trend in generation associated with cell behavior. (A) A total of 128 variables (listed in Methods and S2 Table) related to morphology, location, proliferation, and association with neighboring cells were analyzed using principal component analysis (PCA) for 1384 cells belonging to 28 colonies. k-means clustering revealed no clusters that were especially distinct, indicating that the biophysical properties of these cells behave as a spectrum rather than signifying discrete subpopulations. (B) Color-coding the same PCA plot by generation revealed a trend in PC1-PC2 space. (C) The same PCA plots are color-coded such that only cells from one colony are highlighted, demonstrating that cells from individual colonies did not cluster together. (D) PCA was also conducted on data subsets containing cell properties from only one colony at a time. The trend in generation was observed in individual colonies to varying extents. All plots highlighting individual colonies were chosen at random using the “rand” function in MATLAB.

Upon first inspection, PCA indicated no visually distinct clusters of cells (Fig 3A). Additionally, when ordered by coefficient value, there were no single driving properties dictating a cell’s position in principal component 1 (PC1) and PC2 space (S3 Fig), including the colony identity of the cell. In other words, a given k-means cluster was not enriched for cells from a particular colony, and thus this analysis provided no indication that cells originating within a given colony were more similar to each other than they were to cells from another colony. However, cells within a given cluster were similar in generation of cell division. This trend with generation was readily apparent when rendered in PC1-PC2 space (Fig 3B). For example, Fig 3B shows that cells which were all at Generation 2 were in close proximity in PC space, even though those cells originated from several different colonies, and regardless of the overall proliferative capacity of those colonies (Figs 1 and S1). Fig 3C illustrates this point by highlighting a single colony within the same multi-colony PC space, with each cell in that colony colored by generation. This remarkable trend with generation also persisted to varying extents when we conducted PCA on individual colonies (Fig 3D).

### Visual representations of colony parameters provide prospective indicators of intercolony and intracolony heterogeneity

**Fig 4:**
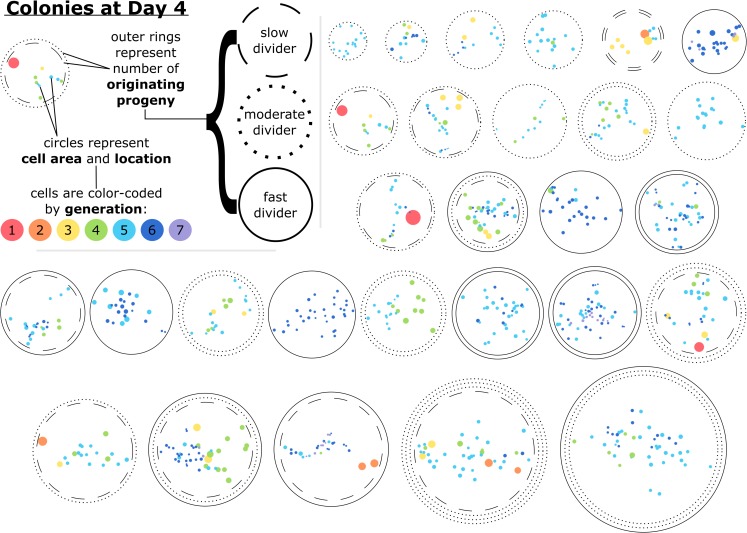
Cells within a colony after four days of growth varied by their size, generation, proximity to other cells, and the proliferative capacity of the progeny they belonged to. Glyphs representative of each colony studied at the single-cell level at Day 4 are presented. Cells are represented by filled circles, which are color-coded by generation and oriented based on the coordinates of the final time point of the single-cell time lapse image analysis. Similarly, the spread area of each cell is represented by the size of the color-filled circles. The outer rings of each colony represent the number and proliferative capacity of the originating progeny; slow dividers had less than three population doublings and fast dividers had more than four population doublings by Day 4. The example provided in the upper-left-hand corner is a representation of a colony after four days of development that originated from two cells, one of which proliferated slowly and one that proliferated moderately. This example colony had 10 cells at Day 4 ranging from first to fifth generation, with the first generation cell being relatively isolated and having a large spread area. The colonies are ordered horizontally by their approximate diameter.

Having identified distinguishing metrics of heterogeneity within a colony, we next considered how the extent of intercolony and intracolony heterogeneity–including properties related to colony diameter, cell area, proliferation kinetics, and cell generation–could be represented visually and concurrently to aid in further classification and insights. Fig 4 visually demonstrates the degree of intracolony heterogeneity in cell generation, as well as these other properties, for all colonies analyzed at the single-cell level up to Day 4. Fig 4 also provides spatial information in which the extent of cell-level heterogeneity in size and position within a colony can be compared readily with colony size and rates of cell division, reinforcing the finding that larger colonies are not necessarily highly proliferative, and smaller colonies are not necessarily single cell-derived (Fig 2).

### As expected, slow-dividing progeny exhibit longer cell lifetimes

Fig 4 further demonstrates the variability in proliferative capacity of the originating progenies contained in each colony, classified as slow, moderate, or fast proliferators as defined above. We compared cells belonging to fast- or slow-dividing progeny with respect to several other key properties (S2 Table), including generation, average and maximum spread area, and lifetime. Cells that belonged to slow-dividing progeny had longer lifetimes, were born of cells that had longer lifetimes, and had more senescent daughters on average (Student’s t-test, p < 0.05). We further analyzed cells belonging to moderate- or fast-dividing progeny to determine whether or not their behavior was different if they belonged to a colony containing a slow-dividing progeny. Statistically, such cells migrated less within the local environment (as quantified by total distance traveled, not persistent trajectory distance) when belonging to a colony containing a slow-dividing progeny and exhibited a larger average spread area 1–2 hours before division (p<0.05), though these differences were small (80 μm and 556 μm^2^, respectively, each approximately 20% of the standard deviation of all cells for those properties). It should be noted that cells did not differ in their proliferative capacity when they belonged to a colony containing a slow-dividing progeny: cells were not statistically more likely to have senescent daughters, and second-generation cells did not produce a significantly different number of progenies, when in a colony with a slow-dividing progeny.

### Asynchronous cell division and senescence drive intracolony heterogeneity

Using cell generation as a metric for heterogeneity, we determined that the standard deviation of generations at Day 4 ranged from 0 to 1.3 generations, with single-cell-derived (SCD) colonies exhibiting statistically smaller standard deviations than multi-cell-derived (MCD) colonies (p<0.001; see S4 Fig). One potential cause for differences in generation at any given time point relates to the differences in lifetimes of cells within a colony. There were several cases of twin cells that, while both proliferative, had notable differences in lifetimes and therefore asynchronicity in mitotic events in the progeny overall (resulting in a greater distribution in cell generation at a given time point). Cells were classified as “asynchronous” relative to their twin if their lifetimes differed by more than one standard deviation of the lifetime of all dividing cells in the study (0.27 days). Such asynchronous cells are labeled in their respective lineage trees in S1 Fig. Twins classified as asynchronous exhibited statistically longer lifetimes compared to twins that divided at relatively the same time (p<0.05), suggesting that asynchronicity within a colony was due to one twin dividing later than expected on average, not earlier. Proliferative twins classified as asynchronous also had more senescent daughter cells (p < 0.05), and second-generation cells classified as asynchronous had fewer total progeny (p < 0.05) when compared to twins that divided at approximately the same time.

**Fig 5:**
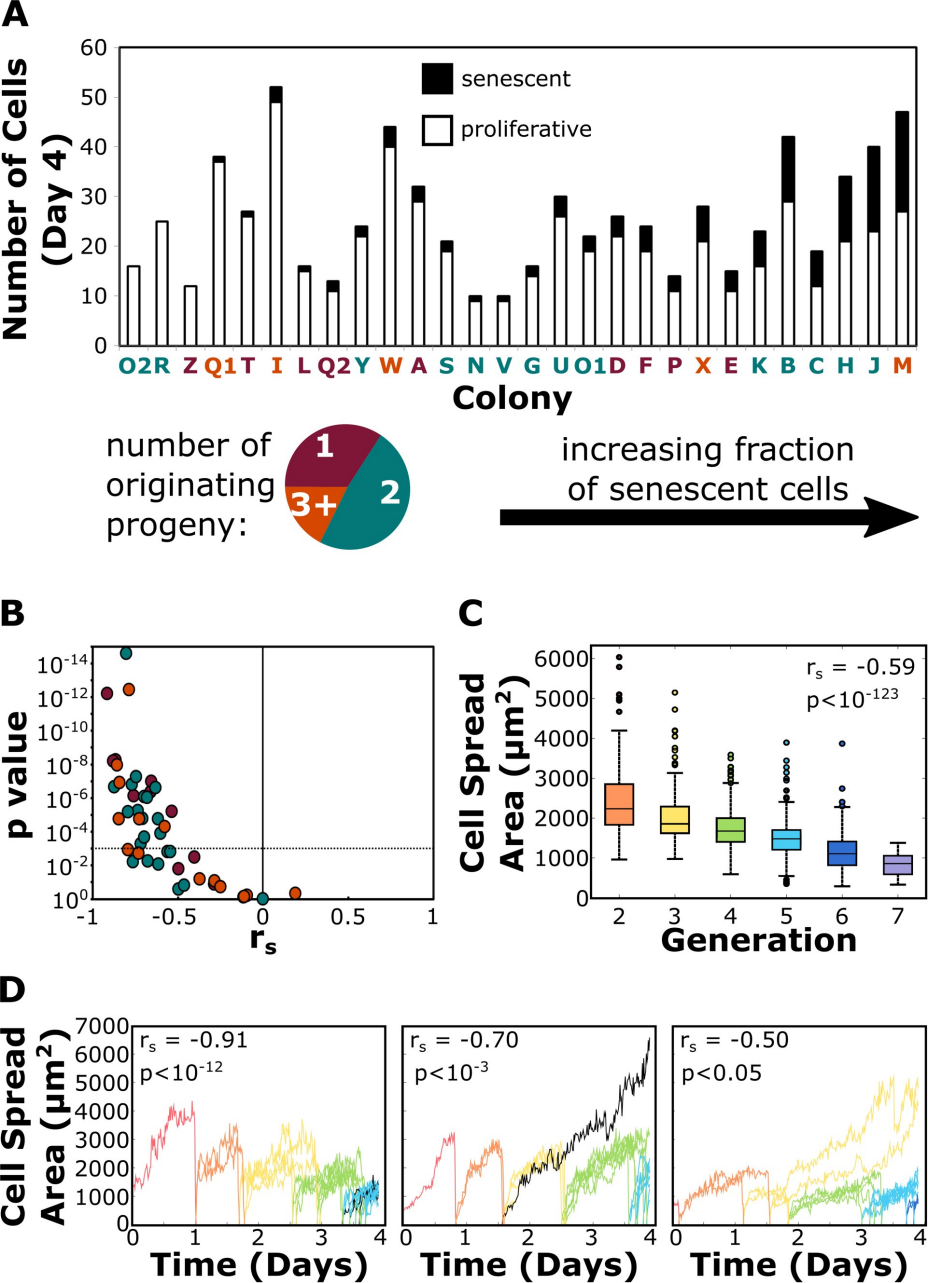
Most colonies contained senescent cells after four days of development, which is partially detectable by deviations in area dynamics. The spread area of cells was measured at every fifteen-minute time point for every colony during the first four days of development. Additionally, cells in each colony at Day 4 were monitored via time lapse videos and marked as senescent if they did not divide by Day 7. (A) The number of proliferative and senescent cells at Day 4 is reported. The color of the colony label represents the number of originating progenies, and colonies are rank-ordered by the fraction of cells that were senescent. (B) The Spearman correlation coefficient *r*_*s*_ and associated p value for the comparison of average cell area versus generation is presented for all progenies contributing to colony formation. The majority of progenies exhibited a negative trend between area and generation to varying degrees of statistical significance. The dashed horizontal line indicates the Bonferroni-corrected p value threshold for a significant correlation, and the color of the progeny data points represents the number of originating cells their respective colony developed from. (C) Cells belonging to all colonies also showed a negative trend between area and generation when their datasets were pooled, where the area of each cell was calculated as the average area of all time points during the first 0.83 days of their lifetimes. The Spearman correlation coefficient and associated p value is also reported (N = 1384). (D) Line plots of individual cell area in single progenies over time are presented for three progenies. Data lines are color-coded by cell generation, with the exception of senescent cells (labeled black). Many progenies uniformly produced smaller cells with each new generation (left); however, heterogeneity onset occurred when cells deviated from this trend, occasionally due to senescence (middle) or from cells having longer lifetimes (right). Also displayed for each progeny is the Spearman correlation coefficient and associated p- value for the comparison of average cell area versus generation, as presented in (B).

Another contributing factor for the distribution of generations within colonies at a given time point was the well-documented phenomenon of senescence in MSC cultures [6,7]. We therefore used the two-week time lapse videos to study the future timeline of cells that were present at the final time point of single-cell analysis (approximately Day 4). Each cell analyzed up to Day 4 was also tracked visually in the time lapse videos to determine whether that cell was still present at Day 7 or had divided into two daughter cells within that time frame up to Day 7. Cells present at Day 4 were considered senescent if they did not divide by Day 7, a particularly stringent criterion given that the average lifetime of all dividing cells studied was 0.83 +/- 0.27 days. Only three of the colonies contained zero senescent cells by Day 4 (one of which was single-cell-derived). The fraction of senescent cells in each colony was highly variable; on average, senescent cells comprised 16% +/- 13% of colonies at Day 4. The number of senescent cells in each colony is presented in Fig 5 and graphically demonstrated in S1 Fig. We found no strong correlations with the number or fraction of senescent cells a colony contained and a colony’s confluency, diameter, degree of isolation, or number or proliferative capacity of the originating cells (S1 Table).

### While larger cells are senescent, successively dividing cells decrease in size with increasing generations

Cell spread area provides an additional and well appreciated property by which to evaluate intracolony heterogeneity (demonstrated in Fig 4), as the largest of cells within a given MSC population have been shown to be committed osteoprogenitors [9] that have exited the cell cycle [7]. In the present study, cells belonging to slow-dividing progeny had larger average and maximum cell spread areas when compared to cells in fast-dividing progeny (p<0.05), and there was a strong positive correlation for all pooled cells between average cell spread area and the maximum area of the corresponding parent cells (r = 0.71, p < 10^−144^, S2 Table). Senescent cells continued to grow over their remaining studied lifetimes (Figs 5 and S5) and had statistically higher maximum and average areas compared to proliferative cells (p<0.01). However, we identified no objective threshold in which all cells below or above a certain cell spread area were predictably proliferative or senescent, respectively (S5 Fig). Contrarily, the average area of many cell progeny decreased with each successive generation; 57% of the progenies exhibited a strong negative correlation between area and generation (p < 0.001), and 96% of progenies had a negative trend between area and generation to varying degrees of statistical significance (Figs 5 and S5). There was also a negative correlation between average area and generation for pooled cells from all colonies and time points, even when the average area was calculated only for the first 0.83 days of the cells’ lifetime (to prevent low-generation, senescent cells from inflating this correlation due to larger cumulative area), as presented in Fig 5C.

### Cell-cell proximity and contact do not correlate with intracolony heterogeneity

An additional metric assessed for intracolony heterogeneity (Fig 4) was the proximity of each cell to other cells in its colony, which generally relates to the potential for cell-cell contact to modify cell division time or phenotype. Previous work has shown that the cumulative and average number of cell-cell contacts (where contact is defined as a cell’s edge bordering another within a distance of 15 px, or 9.7 μm) in sparsely seeded cultures did not coincide with the proliferative capacity of BMSCs [7]. In the present study, the maximum, average, and cumulative number of neighboring cells–as well as the number of neighboring cells at birth–were calculated for the 1384 cells over the first four days of colony development. We found no strong correlation between these properties and several other key properties, including average and maximum spread area, number of progenies, and lifetime (S2 Table). Further, senescent cells on average did not have a statistically different number of neighboring cells when compared to proliferative cells, nor was there a statistically significant difference in number of neighbors between cells that produced two proliferative daughters and cells that produced one or two senescent daughters. There was also no statistical difference in cell contacts between asynchronous dividing twins compared to twins that divided at approximately the same time.

**Fig 6:**
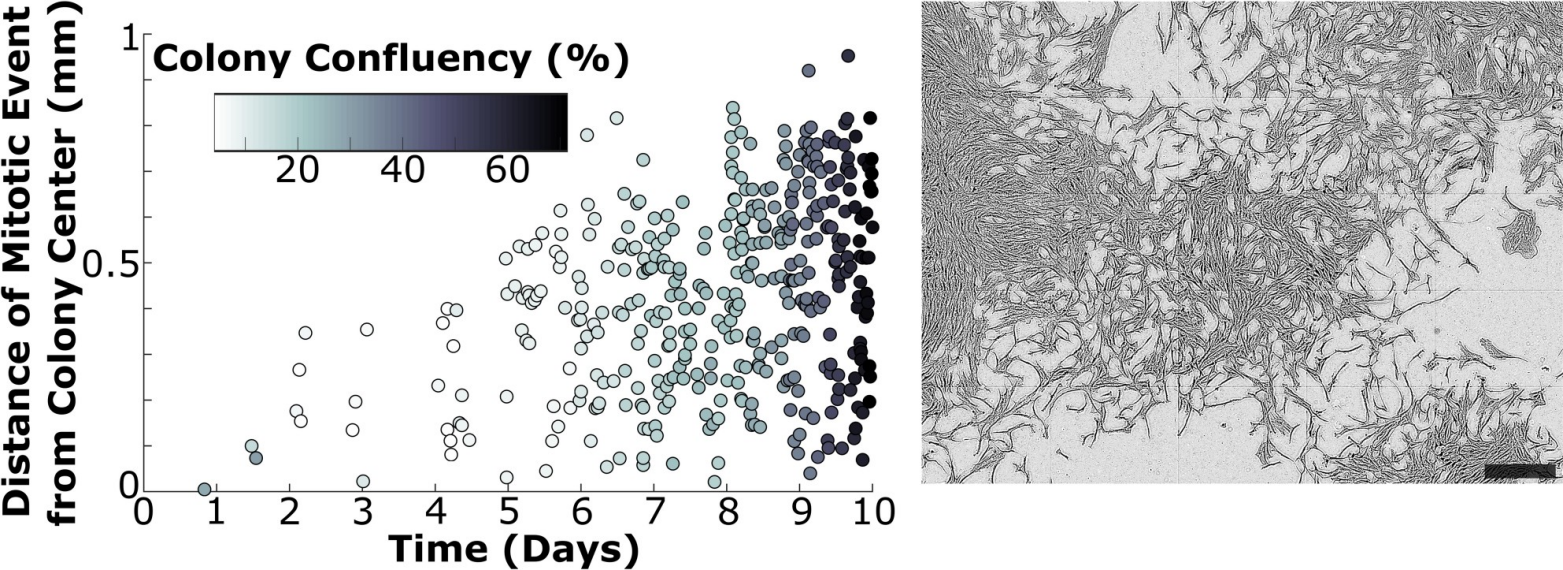
Proliferation continues to occur within confluent colonies. The time and location of each cell division–as well as colony confluency–was measured for the first ten days of colony development in one colony. Mitotic events are reported in terms of their location relative to the colony center at each time point, and the shade of the data points is representative of the measured colony confluency (left). Cells continued to proliferate even at the center of the confluent colony after ten days of development. A phase contrast image of the colony at the final time point is provided (right) as a reference point of the measured confluency. Analysis of a second colony is provided in S6 Fig. Scale bar = 0.5 mm.

There have been several studies describing MSC proliferation as inhibited under confluent culture conditions [20,25,26], including Ylostalo et al. reporting that cells at the periphery of a colony divided more often than those at the more confluent colony center. However, cells in the present study had relatively few contacts compared to the cited studies, as single-cell time lapse analysis was only and intentionally conducted within the first four days of growth at low plating density. We thus carried out further analysis at later time points to consider the effect of colony confluency on cell division. We manually identified every cell division occurring within a monitored colony (using images taken every fifteen minutes as well as the corresponding time lapse video), which we then quantified for time and location (see Methods). We additionally processed and quantified colony centroid location and confluency in CellProfiler. Fig 6A demonstrates that cells continued to proliferate in the center of a colony, even under highly confluent conditions. Fig 6 includes a phase contrast image of the analyzed colony at Day 10 for reference, and this colony was quantified as 67% confluent at this final time point. We carried out this analysis on one additional colony with similar results (S6 Fig), which matched our qualitative observations from studying the corresponding time lapse videos (S8 and S9 Figs).

### Intracolony heterogeneity can emerge even in initially uniform single-cell-derived colonies

**Fig 7:**
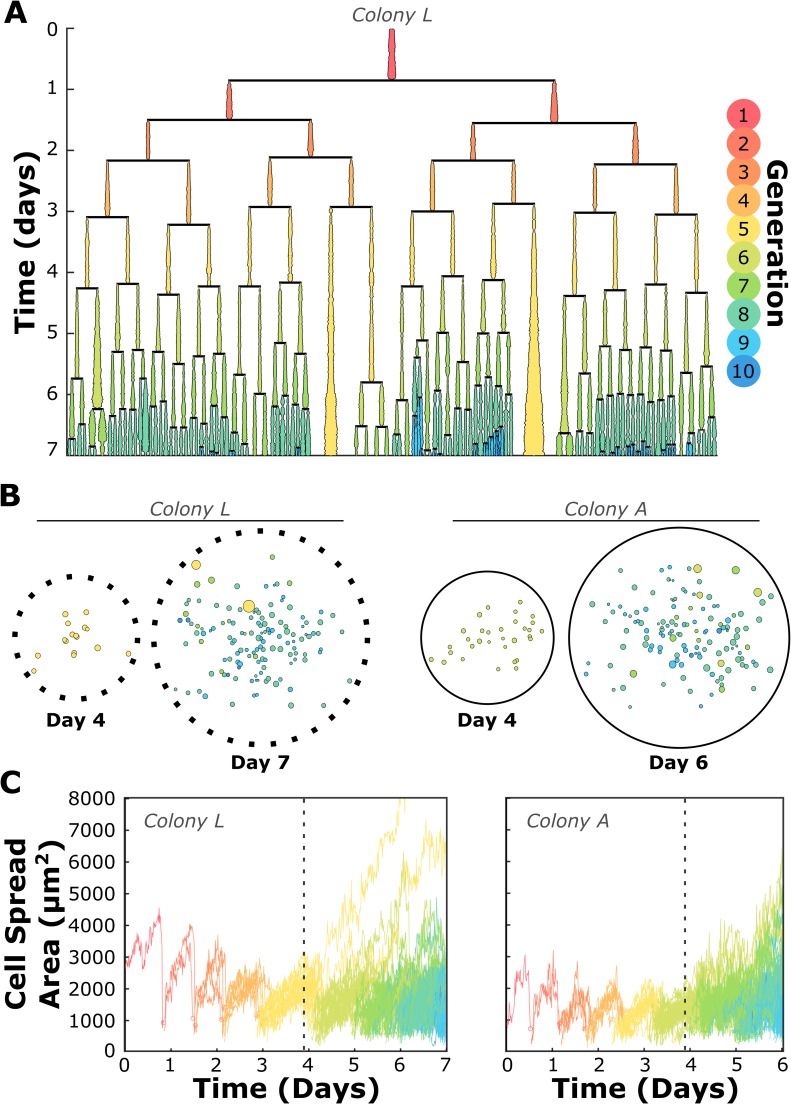
Heterogeneity arose in the most uniform single-cell-derived (SCD) colonies within seven days of development. (A) The lineage tree of one SCD colony studied long-term at the single-cell level demonstrates that even a uniformly-proliferating colony will produce large, senescent cells and asynchronous divisions within its progeny before becoming fully developed. Lineage line widths represent cell spread area at each 15-minute time point. (B) Glyphs similar to those presented in Fig 4 for two SCD colonies (see Fig 4 for key) demonstrate the onset of intracolony heterogeneity. Colony L was classified as a moderate proliferator and consisted of 16 5^th^ generation cells at Day 4, and Colony A consisted of 32 6^th^-generation cells at Day 4. Both colonies developed a distribution in cell area (represented by the size of the color-filled circles) and generation (represented by color) at the later time points indicated. (C) Area-vs-time curves of individual cells for the same two SCD colonies. Graph lines are again color-coded by cell generation. Both colonies presented a decrease in cell area with each new generation, a trend that ceased at approximately the time point the cell medium was replaced (indicated by vertical dashed lines) as part of the conventional BMSC culture protocols of focus in this study.

While most colonies reached a high degree of heterogeneity with respect to cell generation, cell proliferation kinetics, cell area, and degree of cell-cell contacts, there were some colonies which appeared quite uniform in these characteristics for the first four days of growth. We thus conducted single-cell time lapse analysis on an SCD colony for which these characteristics and the corresponding lineage tree were relatively uniform at Day 4 to determine whether such a progeny was capable of remaining homogeneous upon further cell proliferation and colony growth. Fig 7 presents the resulting lineage tree, demonstrating the onset of senescence and diversity in division lifetime among cells of this once-uniform colony. S1 Fig includes a time-lapse video with cells color encoded to match generation, providing a real-time demonstration of the onset of heterogeneity as colony growth continues. Long-term time lapse analysis of two additional colonies is also presented in Figs 7 and S7. In all progenies analyzed beyond Day 4, disruption of these coordinated cell divisions and progressive reduction of cell size with increasing generation occurred approximately at the time of medium exchange (Figs 7 and S7).

## Discussion

### Single-cell origin must be confirmed rigorously

One key finding in this study is the demonstration that cells plated at low density frequently formed apparent colonies which actually comprised multiple originating cells. Unfortunately, we identified no facile predictive visual marker at Day 7 to indicate how many cells a colony originated from, as evidenced by the lack of significant correlation between the number of originating progeny and colony diameter, confluency, or number of neighboring colonies (S1 Table). The lack of correlation can be explained by the observation that colonies were formed by combinations of progenies of varying proliferative potential (Fig 4). For example, colonies F and Y (S1 Fig) each had 24 cells at Day 4 and both exhibited medium confluency at Day 7, yet one originated from a fast-dividing progeny and the other from two moderately-dividing progenies, respectively.

Interpretation of results in many experiments, including the common CFU assay, can be distorted without knowledge of the number and proliferative capacity of progenies comprising each colony. Of the 52 originating cells in the present study that contributed to the formation of 26 colonies by Day 7, 33 had the potential to form a single colony on their own (i.e., they contained 12 cells at Day 4, which was the number of cells in the slowest dividing SCD colony at that time point). Analysis of the CFU number at Day 7 would have thus indicated only 26 colonies, leading to 20% error in the CFU measurement (Fig 2). Another confounding effect of this phenomenon is the observation that several colonies can eventually combine to form one very large colony (see colonies Y and F in S1 Fig). While the CFU assay may be able to distinguish large differences among populations or treatments (as more colonies will generally form if there are more rapidly dividing progenies), such an error rate renders smaller differences among populations as undetectable unless the number of originating progenies is confirmed for each colony.

This finding underscores the need for rigorous methods to verify single-cell origin of colonies in all studies utilizing colony-derived cells. Even the lowest of plating densities cannot guarantee that cells will not attach near each other, and all SCD colonies in this study were infiltrated by nearby migrating cells within seven days of development. While obtaining colonies of single-cell origin might be thought to be ensured by physically isolating single MSCs, our data suggest the need for more rigorous validation than the observation of an apparent single colony in a well several days after plating. Single-cell isolation is not guaranteed with flow cytometry or serial dilution, as these methods rely on a Poisson distribution with a reported success rate of 26% [21], and so each well must be visually inspected for the presence of a single cell. Single MSCs are notoriously difficult to see in meniscus-dominated dishes such as 96-well-plate wells [16], so fluorescently labeling as demonstrated by Russell et al. [21] provides one approach to such verification if such cell labeling has been validated to not interfere with the cell behavior of interest. Another viable option is demonstrated in Choi et. al [27], who distributed cells into microwells and used an AVISO CellCelector to isolate single cells under the guidance of a microscope, enabling them to observe in real time that only one cell was selected for each well. Though such rigorous methods are time-consuming, the frequency of MCD colonies identified in this study indicates that such approaches are essential to ensuring that intracolony heterogeneity is not elevated due to experimental artifact and that the number of colonies formed from a given population is truly representative of the number of progenies that have colony-forming potential.

### Cells in a colony are neither clones nor necessarily stem cells

Despite growing evidence and anecdotal appreciation for emergent or inadvertent heterogeneity within colonies, many experimental designs are fundamentally based on the assumption that all cells derived from the same BMSC colony are homogeneous stem cells. In the present study, we have demonstrated that the morphological and proliferative behavior of MSCs is primarily determined by the generations of the cells (relative to the time point of plating), and not to the colony of origin. In other words, if a functionally uniform subpopulation is desirable, one would obtain a purer population by isolating cells belonging to the same generation rather than cells belonging to the same colony. This finding is evidenced by the trend in generation observed in principal component space (Fig 3B–3D) as well as the observation that cells within a given colony spread throughout PC space rather than clustering (Fig 3C). Even the most uniform and rapidly dividing of progenies in the present study exhibited a broad distribution of cell properties within seven days (Fig 7; see also S7 and S10 Figs), which is still less time than the standard 10–14 days colonies are expanded in typical studies. Further evidence of the functional heterogeneity among cells within MSC colonies is the finding that 91% of colonies contained senescent cells after only four days of expansion–cells that would be considered stem cells by virtue of belonging to a colony and were not, by definition, stem cells. Notably, our criterion in classifying cells as senescent at Day 4 was especially stringent, as cells were not classified as such unless they did not divide before Day 7 –meaning that a cell could be born from cell division just before Day 4, have a lifetime 10 standard deviations above the mean, and still be considered proliferative if it divided before Day 7.

These findings on emergence of biophysical heterogeneity at the intracolony level add to the existing body of evidence of heterogeneity in MSC colonies at the transcription and translation level, as reviewed in Rennerfeldt et al. [16]. For example, Ylostalo et al. [20] found that cells at the inner regions of BMSC colonies were upregulated for genes related to extracellular matrix production, while cells at colony peripheries expressed genes associated with the cell cycle. Similarly, Tremain et al. [28] also concluded that BMSC colonies are heterogeneous in gene and protein expression, reporting that colonies expressed genes associated with late-stage differentiation of several contrasting lineages and were not uniform in expression of various surface markers. However, the design and nature of those prior gene and protein expression studies did not allow for verification of the number of originating cells within each colony at Day 1 (single-cell-derived and multi-cell-derived colonies), nor morphological and biophysical attributes that are more readily accessible during *in vitro* culture.

### Meaningful progress necessitates better understanding of basic MSC behavior in vitro

The onset of heterogeneity within colonies–even those that were known to have originated from a single cell and were initially uniform in area and proliferation kinetics (Fig 7)–demonstrates that any subpopulation, however pure initially, will become functionally heterogeneous within one week of growth under current standard culture protocols. Such heterogeneity and lack of characterization can result in reduced therapeutic outcomes [10] and leads to increased experimental noise for the manifold important MSC studies that do not or cannot employ single-cell resolution. To make meaningful progress in using MSCs as therapeutic agents, several areas of investigation are further needed, including gaining a better understanding of the mechanisms underlying promising results on the benchside, quantifying the therapeutic effects of functionally different subpopulations within a typically expanded MSC population, more concise metrics by which to characterize subpopulations of therapeutically effective MSCs, and sterile and non-retrospective methods to obtain purified populations of MSCs shown to have those critical quality attributes. In order to achieve these milestones, we require increased understanding of the causes of heterogeneity onset under conventional culture protocols.

Though time lapse image analysis is a powerful tool for characterizing cell behavior and can potentially measure hundreds of cell properties, relatively few possible causes of behavioral deviations can be tested without additional coupling to other existing technologies such as RNA sequencing or analysis of surface markers. Nonetheless, some possible causes of heterogeneity onset have been explored in the present study while minimally perturbing the culture system. Heterogeneity was enabled partly by cells within a progeny changing systematically with each new generation. We found that most progenies produced smaller cells with each generation under standard culture conditions (Fig 5). This slow change in cell behavior with each successive generation is further demonstrated by the trend with generation in PC1-PC2 space (Fig 3), which represents a linear combination of dozens of properties. Combined with the observations that overall mitotic events within a colony were rarely synchronous (Fig 6; see also S1 and S5 Figs) and became less so with time (Figs 7 and S7), this slow, asynchronous change in cell behavior with generation and time in culture is one enabler of the eventual onset of heterogeneity in traditional MSC cultures.

Colony confluency surprisingly appeared to have no effect on heterogeneity nor the cells’ ability to proliferate, as mitotic divisions frequently occurred even at the center of a confluent colony after 10 days of growth or more (Figs 6 and S6, S8 and S9), and no strong correlations were found between the average, maximum, and cumulative number of cell-cell contacts and several key properties (S2 Table). The lack of association between degree of cell-cell contacts and behavior does not necessarily conflict with results reported on confluency affecting cell behavior in previous studies. For example, cells grown in extremely confluent conditions–beyond the detailed analysis of this study–did eventually stop proliferating and acquired a more quadrilateral morphology several days after 100% confluency was attained (S8 and S9 Figs). Further, cell-cell contact in this study was defined as cells coming within 15 pixels (9.7 μm) of each other, a metric that does not describe the extent of interaction between the cells, and generally cells had very few cell-cell contacts during their first four days of growth due to being in a sparsely seeded culture. Finally, studies comparing confluent to sparsely seeded cells would naturally yield substantially different properties if confluent cells were cultured for longer periods of time, as longer-term cultures would have higher generations of cells and therefore potentially different characteristics (Fig 3).

In the same vein, an additional cue for the onset of heterogeneity could be in the many factors differentially secreted by various subpopulations in a given culture [10] as well as the dynamics of available nutrients supplied by expansion medium. Between times of medium exchange, secreted factors from cells accumulate in the expansion medium, and available nutrients become increasingly depleted, both with time and with successive cell divisions. One could speculate that this progressive change in the cells’ biochemical environment could explain the change in cell properties with each generation; later generations were born at later time points (r_s_ = 0.90, p< 10^−200^) and therefore were possibly exposed not only to a higher concentration of signaling molecules but also decreased nutrients. (Cell media was not exchanged until Day 4 in this study as part of conventional biweekly media exchange protocol.) Such an empirical trend with generation is particularly interesting considering that the generation assigned to the analyzed cells was relative to the beginning of the experiment. In other words, all “first generation” cells reported here were any number of generations in their previous passages prior to our time lapse imaging experiment. Yet, once the cells were removed from a ~70% confluent culture and plated in very sparse conditions, the cells exhibited biophysical properties more similar to each other than to cells in their own progeny several generations later (Figs 3 and 5). Further, upon initial plating, the so-termed first generation cells had an unusually long growth phase (S5 Fig) that has been documented elsewhere [7]; most first generation cells did not divide until ~1 day after plating (approximately the average lifetime of all cells studied), despite the fact that these cells were presumably at different cell cycle points when harvested at the previous passage. These findings motivate future studies that explore whether media composition (and changes in that media composition over culture time) are causal or correlative in the resetting or adaptation of these cell attributes during passaging.

While it was of primary interest to determine whether or not colonies that appeared homogeneous at Day 4 eventually diversified in our study, analysis of select colonies beyond Day 4 (the time point at which medium was also exchanged, incidentally) was also motivated by the quantitative observation that cell spread area decreased with each new generation. We did not design this study to determine whether heterogeneity was caused by the media exchange protocol or composition, but we recognized that this media exchange step in a conventional culture protocol addresses nutrient depletion and byproduct removal. Indeed, as is demonstrated in Figs 7 and S7, rapidly and uniformly dividing colonies exhibited abrupt and strong deviations from generational trends upon replacement of the culture medium at Day 4. We also note that cells belonging to colonies that contained slow-dividing progeny migrated a shorter distance over the course of their lifetimes (p < 10^−6^) and had a larger area before division (p < 0.01) when compared to cells belonging to colonies containing only fast or moderate proliferators; future studies could be designed to explore whether the local chemical environment (e.g., cell-secreted factors) differ for cells of different proliferative capacity as another possible source for the seemingly stochastic deviations in the generational trends of a given progeny (Figs 5 and S5). Of course, such effects of chemical promoters of BMSC intracolony and population heterogeneity are speculative in the current study design. These observations are included to motivate future efforts in elucidating the effects of expansion medium replenishment and signaling from BMSC subpopulations, as well as contributions thereof to the causes of emergent heterogeneity in proliferating MSCs.

### Conclusions

We provide herein a detailed analysis of dozens of morphological, positional, and proliferative kinetics of colony-forming cells, obtained through time lapse image analysis. We have demonstrated that colonies developed under sparsely seeded conditions (approximately 30 cells/cm^2^) were rarely formed by single cells. Regardless of origin, most colonies demonstrated heterogeneity in terms of proliferative capacity and biophysical properties after only four days of growth (Figs 4 and S1), and even the most uniform single-cell-derived colonies were infiltrated by neighboring progenies and exhibited heterogeneity within seven days (Figs 7 and S7), suggesting that heterogeneity onset even in putative stem cell colonies is inevitable under currently typical culture conditions. Additionally, after only four days of growth, a fraction of cells in nearly all colonies became senescent and by definition were no longer stem cells. Our findings suggest that, typically and unless otherwise confirmed, colonies grown in sparsely plated BMSC cultures are neither accurate representatives of the entire putative stem cell subpopulation, nor collections of functionally homogeneous stem cells originating from a single rapidly dividing mother cell.

This analysis demonstrated that physical cell-cell contacts did not alter the biophysical properties or proliferative capacity of cells, (Figs 6 and S6), a quantification that matched our previous findings [7] as well as our observations of extended time lapse movies of colony formation (S8 and S9 Figs). We have additionally reported that BMSCs, regardless of their proliferative and biophysical history during previous expansion, slowly change their behavior with each successive generation and that cells within the same generation could exhibit behavior more similar to each other than to cells belonging to the same colony or cells in their own progeny born later in time. Such phenomena demonstrate the need for further study of how the traditional culture conditions (and perturbations of such) impact rapidly dividing cells of any origin. More specifically, these findings provide first steps toward developing a better understanding of the mechanisms underlying biophysical and functional heterogeneity onset, in an effort to improve therapeutic outcomes for this promising class of stem cells.

## Materials and methods

### 1. Contact for reagent and resource sharing

Further information and requests for resources and reagents should be directed to and will be fulfilled by the Lead Contact and corresponding author, Krystyn Van Vliet (krystyn@mit.edu).

### 2. Experimental model and subject details

#### 2.1 Cell source

BMSCs were obtained through generous donation by RoosterBio, Inc. (Frederick, MD, USA) at passage 2 (P2). The cells were obtained from a healthy male donor (age 31–45). The cells were screened by the company for positive adipogenic and osteogenic differentiation staining, as well as for biomarkers via flow cytometry with the following results: <5% positive for CD14, CD34, and CD45; >99% positive for CD166, CD105, CD90, and CD73. These cells are commercially referred to by the company as human bone marrow-derived mesenchymal stem cells.

#### 2.2 Cell culture: Expansion and freezing

BMSCs were cultured in expansion medium containing 89% low-glucose Dulbecco’s Modified Eagle Medium, 1% penicillin-streptomycin (10,000 U/mL), and 10% fetal bovine serum (FBS) lot-screened for colony formation, population doublings, and differentiation into the adipogenic and osteogenic lineages. Cells were cultured until 70% confluent, detached using trypsin-EDTA (0.05%) and cryogenically frozen as P3 in a solution of 90% FBS and 10% dimethyl sulfoxide until further use. For experiments, cells were thawed at P3 and plated at 500 cells/cm^2^ in the same expansion medium described above. Cells were fed twice per week and, upon reaching 70% confluency, were trypsinized as P4 cells and replated for experimentation. All reagents were purchased from ThermoFisher Scientific (Waltham, MA, USA).

### 3. Method details

#### 3.1 Cell plating density assay

**3.1.1 Motivation.** To our knowledge, an assay to estimate actual plating density used in experiments involving BMSCs has not been published prior to this work. In our time lapse imaging experiments, we used glass Petri dishes rather than the traditional tissue culture polystyrene ones to optimize image quality. While we did plasma treat the glass approximately two hours prior to seeding, we hypothesized that fewer cells would attach to the dish than expected, motivating the development of a protocol to estimate actual plating density.

**3.1.2 Plating into Petri dishes using limiting dilution.** Petri dishes that were 60 mm in diameter and that each contained a glass well 35 mm diameter were plasma treated for 10 minutes using a plasma chamber (Diener Electronic, Ebhausen, Germany). Dishes were then placed under a UV light in a sterile tissue culture hood for approximately two hours while cells were counted and diluted. Prior to plating, dishes were also rinsed with water to remove any dust acquired from plasma treating and to ensure the glass surface was hydrophilic.

BMSCs were expanded to 70% confluency in T-175 flasks and trypsinized as P4 cells. Following trypsin neutralization and centrifugation, the cells were counted using a standard glass hemacytometer. After counting, cells were pipetted again to ensure uniform suspension, and 10% of the cell solution was transferred to a microcentrifuge tube and diluted with expansion medium to 1 mL. Cells in the 1 mL suspension were again pipetted, and 100 μL of that 1 mL was pipetted into a new centrifuge tube and diluted to 1 mL. This process was repeated until a suspension of approximately 1,000 cells/mL was achieved (usually 3 dilutions were needed). The final suspension of cells was then plated into the glass well of a Petri dish at a theoretical density of 100 cells/cm^2^. This entire process was then repeated for each Petri dish using a new, unique dilution, where one experiment consisted of five dishes.

**3.1.3 Plating density measurements.** After approximately six hours (the time at which imaging began in the main experiment), cells were fixed and stained with a 0.5% solution of crystal violet (Sigma Life Science, St. Louis, MO, USA) in 100% methanol (Sigma-Aldrich, St. Louis, MO, USA). All cells present in each dish were then counted (N = 5 dishes for each of two experiments), and the plating density was calculated by dividing the number of cells by the surface area of the glass well. Counting cells was aided by drawing a grid on the bottom of each dish in a fashion akin to a hemocytometer. In the first experiment, the plating density was 17 +/- 5 cells/cm^2^, and in the repeat experiment the density was 29 +/- 4 cells/cm^2^, from which we have concluded that, in our hands, a theoretical plating density of 100 cells/cm2 on glass-bottomed dishes leads to a much lower actual plating density.

#### 3.2 Time lapse imaging

**3.2.1 Initial image acquisition.** Time lapse imaging of developing colonies was conducted in two separate, identical experiments. For each experiment, BMSCs were plated into five plasma-treated glass Petri dishes in the same fashion as outlined above (see *Plating into Petri Dishes using Limiting Dilution* in the Cell Plating Density Assay section prior to this one). After approximately six hours, dishes were inspected to ensure cells had attached. The dishes were then assessed for how far apart individual cells were. The dish with the most isolated cells was used in time lapse imaging, and the remaining four were kept in an incubator at 5% CO_2_ and cultured in parallel as positive controls of cell growth. Expansion medium was added to all dishes such that the total volume was 5 mL.

Imaging was done with the petri dish kept in a stagetop incubator (Tokai Hit, Shizuoka, Japan) set at 5% CO2 and the water bath kept filled with filtered, autoclaved water for maximum possible humidity. A temperature probe was kept submerged in the MSC expansion medium at all times as a feedback sensor so that the stagetop incubator could be kept at 37°C, and the instrument was calibrated for temperature control before each use. Imaging was done with an Olympus IX81 microscope (Olympus, Shinjuku, Tokyo, Japan) with a phase contrast 10x objective. Beginning at the top of the glass well, the dish was systematically scanned left to right, top to bottom for attached cells. Cells that were especially large and circular in morphology, reminiscent of the often-cited “Type II” morphology [8], as well as cells that had more than 2 neighbors in the field of view (FOV), were omitted from imaging; the location (microscope coordinates) of all other cells was captured using Metamorph’s Multidimensional Data Acquisition application, with the cell(s) of interest positioned at the center of the FOV. This process was carried out to capture the location of as many cells as possible over the course of one hour in an effort to begin imaging before cell division occurred, resulting in the capturing of 55 locations in one experiment and 70 locations in the second. Once the locations were recorded, a MATLAB script was used to calculate the appropriate stage coordinates such that each location had a 2x2 FOV grid (1.7 x 1.3 mm). These coordinates were saved as a text file and imported back into Metamorph. Time points were set to every fifteen minutes, and imaging began approximately 7.5 hours after initial plating.

**3.2.2 Monitoring of colony development.** As the BMSCs migrated and proliferated into colonies, there was a need to increase the number of fields of view (FOV) during the course of the 14-day experiments. However, because our microscope stage was limited to being able to capture 400 images per 15-minutes time point, locations being monitored needed to be omitted with each FOV expansion. This monitoring was made possible through a MATLAB script that organized images by the location they belonged to, stitched the images into a montage, and made a video (see S8 and S9 Figs). The script ran every hour in order to reduce the total number of files in the output folder of Metamorph and to ensure that any videos monitored would represent the most current state of each location.

Videos were monitored daily for colony growth. Approximately two days after initial seeding, some of the monitored cells were close to migrating out of the FOV grid, and so the number of FOVs for each location was increased from 4 (2x2 FOVs, 1.7 x 1.3 mm) to 9 (3x3 FOVs, 2.6 x 2.1 mm). Locations in which cells were proliferating slowly (or not at all) were omitted from further monitoring in this transition. This process was continued until the FOV grid needed to be expanded again to a 4x4 FOV grid (Day 4, 3.5 x 2.6 mm) and finally a 5x5 FOV grid (Day 8, 4.3 x 3.3 mm). A total of 15 monitored locations remained in each experiment after the transition to a 5x5 FOV grid, and these areas were monitored for approximately 14 days. Out of the combined 30 colonies, 28 were suitable for analysis, while the other two migrated too rapidly for single cell tracking. In two of the remaining 28 locations, the colony under development at Day 4 merged with another nearby analyzed colony by Day 7 (see colonies O1/O2 and Q1/Q2 in S1 Fig). Both experiments resulted in approximately 1.1. terabytes of raw data (phase contrast images) each.

#### 3.3 Single-cell measurements of time lapse images

**3.3.1 Image processing and cell segmentation.** Several specific and detailed steps were required in order to track cells in each colony at the single-cell level. All colonies were processed and tracked as individual datasets, and estimates for the time required of each step for a typical colony are provided. The first step consisted of masking the images in an attempt to remove all background pixels and was performed using the phase contrast reconstruction algorithm proposed by Yin and Kanade [29], which minimizes a quadratic cost function and then applies thresholding in an attempt to segment cells. All acquired phase contrast images of the 28 aforementioned locations (approximately 25,000 images per colony) were masked using high-performance clusters located at the Massachusetts Green High-Performance Computing Center (Holyoke, MA, USA).

Masked images were then run through a simple pipeline in CellProfiler version 2.0.11710 [30] that identified all objects (i.e., BMSCs and artifacts not removed in masking) in the image, converted those objects into a new image, and saved that image separately. The resulting “identified objects” (IO) image was a representation of how CellProfiler interpreted individual cells; each interpreted object was a different color, and touching cells that were not successfully segmented would appear as the same color. From there, side-by-side stacks of images–one stack of the original phase contrast images, and one stack of the identified objects images–were opened side by side in ImageJ [31]. The windows synchronization tool enabled researchers to correct the identified objects images by directly tracing the phase contrast images with the paintbrush. The following corrections were made to the identified objects images: 1) all cells present in the colony that belonged to the originating progeny had a 10-pixel white dot placed on them as a “seed” for downstream processing, 2) in the case of larger, flatter cells, the cells were outlined to connect missing pieces of the cell caused by overly-aggressive masking, and 3) cells that were touching (as shown by appearing as the same color in the identified objects images) were segmented by drawing a null-pixel (black) line 5 pixels wide between their touching borders. Cells that were touching (i.e., sharing perimeters, and therefore not easily identified as separate objects in a single static image) were often identified by researchers by flipping through several images at previous and future time points. For each colony, images corresponding to 374 15-minute time points were processed in this way and took approximately 22–70 hours per colony (depending on the size and complexity of the colony). These adjusted identified objects images were then processed in a second CellProfiler pipeline that filled in all holes created by cell outlines and eliminated any objects not seeded in the previous step (approximately 12–17 hours per colony, depending on the available memory and processing power of the computer used). These processed images were then saved, inspected to ensure they were representative of the phase contrast images (e.g., no cells were missing, and no debris was present), and then submitted to tracking in CellProfiler.

**3.3.2 Tracking and measurements in CellProfiler.** Images processed using the methods in the previous section were run through a simple pipeline in CellProfiler that identified primary objects, assigned those objects numbers, tracked the objects, displayed the object numbers on the images, and saved the images (approximately 22–36 hours per colony, depending on the available memory and processing power of the computer used). The resulting output file was then run through a MATLAB script (versions 2014a through 2016a; The MathWorks Inc., Natick, MA, 2000) that assigned a unique identifier to each cell (e.g., if one cell was present at day 1, it was assigned an ID of 1, and its children IDs of 2 and 3 at later time points) as well as the corresponding object number of that cell at every time point. The result was a two-dimensional matrix of object numbers, with rows corresponding to each of 374 time points and columns corresponding to each unique cell over time. Cells that did not exist at any given time point (e.g., the originating cell after it divided into two unique daughter cells) were given an object number of 0. This matrix was exported as an Excel spreadsheet for further analysis.

The resulting spreadsheet was formatted in Excel to highlight all cells with a value of 0 for ease of visualization. The spreadsheet was then inspected for tracking errors: a given column contained non-zero object numbers for all time points (rows) the corresponding cell was in existence. At the time point a column began containing zeros (signifying a supposed division), two columns previously containing zeros would begin yielding non-zero object numbers (signifying the daughter cells of the dividing cell). Any instances where this was not the case was considered an error and subject to further investigation. Further, the object number of a dividing cell at its final time point, as well as the object numbers of the corresponding daughter cells at the next time point, were noted and verified in the tracked images (which displayed the object number of each cell at each time point). Any errors found in this manner (for instance, two cells that were not properly segmented for a time point and therefore appeared in the spreadsheet as one cell disappearing without two new cells appearing) were corrected, and the image set was run through the tracking pipeline again. This process was iterative, with the total active time for correcting each colony amounting to approximately 2–3 hours (depending on the complexity of the colony and the number of initial errors), and the tracking process requiring approximately 22–36 hours for each iteration (depending on the memory and processing power available for a given computer used).

Once no apparent tracking errors were present, the images were run through a more complex tracking pipeline in CellProfiler, which included several more modules in addition to the ones in the previous pipeline. These modules measured the area occupied in each image, information about neighboring cells for each cell (where a neighbor was considered at 15 pixels away), and information about the cells’ size and shape (approximately 70–110 hours per colony, depending on the computing power of the computer used). The pipeline further saved images of the identified objects as .mat files (which, when loaded into MATLAB, yielded a 2D matrix of every pixel in the image, where the values were either 0 for a null pixel or the object number for a pixel belonging to an identified object) for further use in data visualization (see “Procedures for Data Visualization” below). The resulting output file was then run through a MATLAB script that constructed cell lineage trees (with the width of the lineage lines representative of cell spread area, developed for Whitfield et al., 2013), which were inspected for any further possible errors. Errors spotted, if any, were corrected and run through the extensive pipeline again. The occurrence of errors at this stage was rare and took less that one hour to correct (plus an additional 70–110 hours to run through CellProfiler again). The resulting output file for each colony was analyzed further using MATLAB (see “Single-Cell Time Lapse Image Analysis” in the Quantification and Statistical Analysis section below).

Overall, each colony required 4–8 weeks of full-time work (where a week here is defined as 50 hours, with 100% of time devoted to processing) in order to process 374 images per colony at the single-cell level. Together, all processed colonies are a result of the combined efforts of 25 researchers, though we note that improvements and semi-automation of the processing over this period resulted in reduced human time required for validated image processing.

#### 3.4 Procedures for data visualization

**3.4.1 Image overlay to represent cell generation and progeny (Figs 1 and S1).** During tracking, .mat files were saved for each image (time point) in a given colony. Uploading these files into MATLAB displayed a 2-D matrix representative of the image, where each entry in the matrix was a value corresponding to a pixel. The values were 0 for all null-pixels; pixels belonging to an object (cell) identified in the image through the tracking pipeline described above contained the object number in the corresponding location of the matrix. These files were required for relating the analyses of the cells (described below; see Quantification and Statistical Analysis) to the cells qualitatively seen in the original phase contrast images (see S1 Fig, Day 4 images). Using the same MATLAB script employed to construct lineage trees, the object number of each cell in the final time point analyzed was mapped to that corresponding cell’s generation and originating progeny ID.

The original phase contrast images were adjusted for brightness and contrast using GIMP (www.gimp.org). A MATLAB script then conducted the following steps to create images with overlays corresponding to cell generation and progeny presented in Figs 1 and S1) imported the adjusted phase contrast image for the final time point, 2) imported the identified objects image and corresponding .mat file described above, 3) calculated the generation and originating progeny of each cell in the image (based on the tracking output file and the code used to generate cell lineage trees), along with the cells’ corresponding object number, 4) changed the RGB color value of all pixels corresponding to a given object in the identified objects image according to either the numeric value of their generation or their progeny, and 5) overlaid a transparent version this new image on top of the phase contrast image imported.

**3.4.2 Representation of whole-colony properties (Fig 2).** Phase contrast images at the 672^nd^ time point (corresponding to the final image of Day 7) were masked using the same technique described above (see “Image Processing and Cell Segmentation” above). All cells not belonging to the colony at that time point were removed from the image using a black paintbrush in ImageJ. The images were then inverted and saved as binary images. Binary images were imported into Inkscape (www.inkscape.org), and the color of the all black pixels in a given image was changed to represent the number of originating progenies belonging to that colony (as determined by studying the corresponding time lapse video from the first time point onward).

**3.4.3 Representation intracolony heterogeneity (Figs 4 and S1).** For a given colony, the output file from single-cell tracking in CellProfiler (see “Tracking and Measurements in CellProfiler” above) was imported into MATLAB, and from the dataset a matrix was constructed containing the x-coordinate, y-coordinate, generation, and cell spread area of each cell at the 374^th^ (approximately Day 4) time point was constructed. The x- and y-coordinates of each cell were then plotted in a scatter plot, with the color of the data point representing the cell generation and the size of the data point set to be relative to the corresponding cell area. The plot was then imported into Inkscape, where rings were constructed to surround the cells, representing the number and proliferative capacity of the progeny comprising the colony. For S1 Fig, a scale bar of arbitrary length (that was consistent for each colony) was added to each glyph, and the two combined were enlarged to fit the space available. Data points corresponding to senescent cells were manually marked with a “/” (see “Classification Analyses” in the Quantification and Statistical Analysis section below).

### 4. Quantification and statistical analysis

#### 4.1 Single-cell time lapse image analysis

**4.1.1 Description of data formatting.** For a given colony, the output file generated from the tracking pipeline in CellProfiler described above was reorganized in in two general ways: by constructing an array of all raw properties measured in CellProfiler (reorganized by time and unique cell identifier), and by constructing a data table of manually-calculated cell properties based off of the raw measurements. In the former, a unique identification number was assigned to each cell using the same code used to construct cell lineage trees described above. Using this ID and the object numbers of cells at each time point, the data matrix was transformed into an array with properties as columns, time points as rows, and each matrix entry a vector containing the numeric values of a given property and time point, in order of unique cell ID. The second data set constructed was a subset of manually-calculated properties (such as the average and maximum spread area over the course of each cell’s lifetime). These properties are tabulated in S2 Table.

**4.1.2 Correlation analysis.** The Spearman’s rank correlation coefficient (r_s_) and associated p value for each pairwise comparison for all pooled colony datasets of the manually-calculated properties described in the paragraph above were calculated using the corr function in MATLAB and are presented in S2 Table (created using Excel). For each property, appropriate filters were carefully considered so as not to skew the statistics due to unrepresentative values. For example, cells which did not have a documented lifetime–either from being a first-generation cell (and therefore an unknown birth time point), a senescent cell, or a cell that did not divide within the first four days of the experiment–were filtered out of the pooled dataset before calculating the correlation coefficient between lifetime and all other listed properties. Any filters used for each property are indicated in S2 Table. Correlation coefficients and p values are highlighted in green for any pairwise comparison whose p value passed the Bonferroni-corrected p-value threshold (1.8 x 10^−4^, calculated with an initial p value of 0.05, divided by 276 pairwise comparisons). All p values less than 0.05 and greater than the Bonferroni-corrected p value threshold were highlighted in blue. In the main body, statistical significance is only reported for coefficients passing the Bonferroni-corrected p-value threshold.

**4.1.3 Principal component analysis (PCA).** Three types of datasets were constructed and combined to form an input matrix for PCA. The first dataset consisted of all properties measured by CellProfiler for all cells (see Table 1 below), averaged over each cell’s lifetime. The second dataset also contained all properties measured by CellProfiler for all cells, but at the time point of each cell’s birth. The third dataset consisted of all manually-calculated properties present in S2 Table. All data columns created by CellProfiler that corresponded to automatic tagging of the data in each frame (e.g., object numbers, which will naturally increase with increasing number of cells in the imaged frame), properties belonging to the progeny as a whole (e.g., CellProfiler’s integrated distance property, which measures the sum of the cumulative distance migrated for a cell and all of its ancestors), or properties that were replicates (e.g., the average area calculated as part of all CellProfiler properties and the average cell area present in the manually-calculated data matrix in S2 Table) were filtered from the PCA input matrix. The table below lists all 54 properties, as measured and named by CellProfiler, that we retained for further analysis.

**Table 1:** All properties measured by CellProfiler at every 15 min time point and considered in further analysis.

Location_Center_X	AreaShape_Zernike_5_5
AreaShape_Zernike_7_5	AreaShape_Zernike_5_3
AreaShape_Zernike_7_7	AreaShape_Perimeter
AreaShape_Area	AreaShape_FormFactor
AreaShape_EulerNumber	AreaShape_MajorAxisLength
Neighbors_AngleBetweenNeighbors_15	AreaShape_Zernike_9_1
AreaShape_Center_Y	AreaShape_Zernike_4_4
AreaShape_Center_X	AreaShape_Zernike_4_2
AreaShape_Zernike_6_6	AreaShape_Zernike_1_1
AreaShape_Zernike_0_0	AreaShape_Zernike_4_0
AreaShape_Compactness	AreaShape_Zernike_9_3
Neighbors_SecondClosestDistance_15	AreaShape_Zernike_8_8
TrackObjects_DistanceTraveled_50	AreaShape_Zernike_9_7
AreaShape_MinorAxisLength	AreaShape_Zernike_5_1
Neighbors_FirstClosestDistance_15	AreaShape_Zernike_9_5
AreaShape_Zernike_7_3	AreaShape_Zernike_8_2
AreaShape_Zernike_3_1	AreaShape_Zernike_6_4
AreaShape_Extent	AreaShape_Zernike_8_0
AreaShape_Zernike_3_3	AreaShape_Solidity
AreaShape_Zernike_9_9	AreaShape_Zernike_8_4
AreaShape_Zernike_2_0	AreaShape_Eccentricity
AreaShape_Zernike_2_2	Parent_cells
AreaShape_Zernike_6_2	Neighbors_NumberOfNeighbors_15
AreaShape_Zernike_6_0	AreaShape_Zernike_8_6
Location_Center_X	AreaShape_Orientation
AreaShape_Zernike_7_5	Neighbors_PercentTouching_15
AreaShape_Zernike_7_7	TrackObjects_TrajectoryX_50

PCA was conducted using the pca function in MATLAB, and plots of principal component (PC) 1 and PC2 were constructed using scatter and gscatter in MATLAB. Each cell’s data point was color-coded by the generation it belonged to, the colony it belonged to, and the cluster ID it belonged to (as determined by k-means clustering using the kmeans function, where the number of clusters was set to the square-root of N divided by 2). This analysis was performed for each individual colony as well as for all colonies pooled. Similarly, PCA was also performed with all measured CellProfiler properties at specific time points (Days 1, 2, 3, and 4) for all cells in existence at that time point in all colonies combined (S3 Fig).

**4.1.4 Colony infiltration.** Time lapse videos of all developing single-cell-derived colonies were studied at various speeds and directions in time to assess the general time a given colony expanded to the point of approaching nearby colonies or non-migratory cells. During this assessment, it was observed that nearby cells not belonging to the colony were migrating and infiltrating the colony borders, prompting further exploration. In order to determine which, if any, cells in a perceived colony at Day 7 did not actually belong to the originating mother cell at Day 0, an image of a colony was opened in ImageJ for the final time point of Day 7, and the time lapse video of the colony was played backwards and forwards from Day 7 to the start of the experiment until four or more infiltrating cells were identified. Such cells were marked by transparent red dots on the Day 7 phase contrast images in S1 Fig (Colonies A, D, E, F, L, P, Q2, T, and Z).

#### 4.2 Analysis of mitotic events

We have developed a novel method for manually tracking the mitotic events of BMSCs in time lapse imaging. For a given colony, images were masked for the first 960 time points (corresponding to ten days of development) using the methods described above (see “Image Processing and Cell Segmentation” above). Cells not belonging to the colony at each time point were then eliminated using a black paintbrush in ImageJ. In some instances, images were further corrected by eliminating cells at the periphery of the colony if they protruded significantly away from the other cells (which would drastically lower the measured confluency in a way not representative of the overall colony area). Confluency was measured at every time point by running these images through a MATLAB script that measured the area occupied by the colony’s convex hull (calculated with the bwconvhull function). As a quality control, images displaying the union convex hull were also saved, overlayed on top of the original phase contrast images, and inspected to ensure the measurements were representative of the space occupied by the colony. The area occupied by the cells in the colony was measured by running the masked images through a simple pipeline in CellProfiler that identified primary objects and measured the area occupied by objects in the image. The resulting output file was imported into MATLAB, and the center of the colony at each time point was measured as the centroid of all objects at a given time point.

The time and location of mitotic events within the colony were evaluated using a combination of ImageJ and CellProfiler. In ImageJ, two stacks of images were opened side-by-side: a stack of the original phase contrast images and a stack of images the same size containing entirely zero-value pixels. The windows synchronization tool was then utilized, which allows for simultaneous scrolling through time points as well as for making marks added by the researcher in one stack appear in the other. Researchers then flipped through time points, and for every cell division (evidenced by a cell suddenly balling up into a small sphere, followed by two cells emerging), a white dot was placed on the dividing cell with the paintbrush tool at the time point just before division. Dots were placed on the original phase contrast images, and so appeared instead on the corresponding blank image in the same location. This process was conducted for 960 time points per colony, and blank images (including those that were marked with a cell division) were saved in order of their representative time point. These images were then run through a simple pipeline in CellProfiler that identified the pixel location of each marked division for all time points. The resulting output file was then imported into MATLAB, and the distance of each mitotic event from the colony center (calculated as described above) was tabulated and plotted.

#### 4.3 Classification analyses

Classification processes of individual cells and colonies are described below and were used in statistical analyses described either above in “Single Cell Time Lapse Image Analysis” or below in “Whole-Colony Analysis.”

**4.3.1 Senescence.** For each colony, an image at 374^th^ time point (the final time point analyzed for single-cell time lapse imaging), which displayed the object number of each cell, was used as a reference point in determining which cells divided at future time points (referred to as the “reference image” from this point forward). Time lapsed videos as well as individual images at time points beyond the reference image were then studied extensively; each cell present in the reference image was tabulated by its object number and manually tracked in the videos and/or images. Cells that divided within the next three days after the reference image were marked as proliferative, and cells that did not divide were marked as senescent.

**4.3.2 Degree of isolation.** Time lapse videos of each colony were studied and scored for how isolated they were from neighboring cells as they developed. A scoring system categorized colonies with a score of 1 through 5 for the following observations: 1) the colony developed with other cells present in the 2x2 fields of view (1.7 x 1.3 mm) from the start of the experiment (neighboring cells were close enough to appear in the combined field of view but migrated out of view and therefore could not be tracked); 2) neighboring cells crawled into the combined fields of view (FOVs) within the first two days of the experiment; 3) neighboring cells were revealed when the number of FOVs was expanded from a 2x2 to a 3x3 grid (2.6 x 2.1 mm) at Day 2; 4) neighboring cells migrated into the combined FOVs after they were first expanded, approximately 2–4 days after the start of imaging; 5) neighboring cells were not revealed until the FOVs were again expanded from a 3x3 to 4x4 grid (3.5 x 2.6 mm) at Day 6.

**4.3.3 Asynchronicity of cell lifetime preceding cell division.** The mean and standard deviation of lifetimes for all cells belonging to all colonies existing at any time point during the single-cell analysis (i.e., within the first four days) were calculated in MATLAB. The difference in lifetimes between each cell and its twin was then calculated, and cells differing in lifetime by more than the overall standard deviation (0.27 days) were tabulated as “asynchronous” for further analysis.

**4.3.4 Fast vs. slow proliferators.** Lineage trees of each progeny contributing to colony formation were studied to tabulate how many cells were present at the final time point analyzed at the single-cell level. Progenies were categorized as “slow dividers” if they contained less than eight cells at the final time point (corresponding to three population doublings), “fast dividers” if they contained more than 16 cells at the final time point (corresponding to four population doublings), and “moderate dividers” if they contained 8–16 cells at the final time point. Each colony was then tabulated in terms of the number of fast, moderate, and slow dividers they comprised of (S1 Table), and each cell was classified by the type of progeny it belonged to. Similarly, all cells belonging to a colony that contained a slow-dividing progeny were tabulated.

**4.3.5 t-tests on binary properties.** All single-cell properties having two possibilities for a given category were compared in a t-test for all manually-calculated properties displayed in S2 Table. These comparisons of “binary” classifications are listed as follows: senescent vs proliferative cells; cells belonging to a fast-dividing progeny vs cells belonging to a slow-dividing progeny; cells classified as asynchronous with their twin vs cells that were not; cells that belonged to a colony with a slow-dividing progeny vs not; and cells that had one or two senescent daughters vs. not. For each property tested in these binary classifications, a filter was applied where applicable so as not to skew results with nonrepresentative or nonsensical data. For example, when comparing the lifetime of cells for one binary category versus the other (e.g., the lifetime of cells classified as asynchronous vs cells classified as not asynchronous), all cells not having a documented lifetime–either by not dividing over the course of the experiment or by being a 1^st^-generation cell whose birth time point was unknown–were omitted from the student’s t-test. The filters applied for each property tested are listed in S2 Table. The threshold for significance was a Bonferroni-corrected p-value of 0.05 divided by the number of comparisons being made for a given filter. For example, in looking at properties of cells in terms of their parents, three properties–maximum cell spread area of the parent, cell spread area of the parent before division, and lifetime of the parent–were analyzed by excluding all 1^st^- and 2^nd^-generation cells (whose parents have unknown properties due to the nature of the experiment). The p-value threshold for significance for these three properties under this filter was therefore 0.05/3 = 0.167.

#### 4.4 Whole-colony analysis

**4.4.1 Measurements.** Image processing was performed on all colonies at the 672^nd^ time point (corresponding to seven full days of colony development) as described above (see “Representation of Whole-Colony Properties” in Method Details). For each colony, the area occupied by the colony’s convex hull was calculated in MATLAB using the bwconvexhull function, and the approximate diameter reported was calculated from this measured area. In the few cases where the colony observed at Day 7 was much larger than the fields of view, the fraction of the colony imaged was approximated by visual inspection and factored into the calculations accordingly. Similarly, the confluency of the colony was calculated as the total area occupied by the cells divided by the area of the convex hull (described in more detail in “Analysis of Mitotic Events” above). The number of neighboring colonies at Day 7 was tabulated by careful observation of the original phase contrast images at the 672^nd^ time point by multiple observers. All other colony-wide properties, such as the number of senescent cells at Day 4, were tabulated from the results of the classification analyses described in the previous section.

**4.4.2 Statistical analysis.** The Spearman’s rank correlation coefficient (r_s_) and associated p-value for all combinations of pairwise comparisons for the 14 whole-colony properties described above (listed in S1 Table) was calculated using the corr function in MATLAB. The results of that analysis are reported in S1 Table, which was created using Microsoft Excel. The critical threshold for r_s_ was +/- 0.619 [24] using the Bonferroni-corrected p value of 0.005 (calculated with an initial p value of 0.05, divided by 105 pairwise comparisons. In the main body, statistical significance is only reported for coefficients passing the Bonferroni-corrected p-value threshold. A student’s t-test was also performed to compare single-cell-derived colonies to multi-cell-derived ones for all listed properties using the ttest2 function in MATLAB and assuming unequal variance among the experimental groups.

#### 4.5 Generation trend calculations

Upon studying plots of area-vs-time for individual cells in a given progeny (see Figs 5D and 7C for examples), it was observed that cells appeared to have a lower spread area with each successive generation. This prompted us to quantify this observation via the Spearman’s rank correlation coefficient for area vs. generation in each progeny. To account for cells with unusually long lifetimes or for cells that were senescent, the area of each cell was defined as the average area over the first 0.83 days (the average lifetime of all cells in all colonies at all time points). This prevented large, low-generation cells from biasing the calculated correlation coefficient to higher statistical significance. The correlation coefficient between cell generation and this modified average area was calculated for each progeny using the corr function in MATLAB.

## Supporting information

S1 Fig In-depth profiles of every colony studied at the single cell level. **Top left:** phase contrast image of the colony at after four days of growth. Cells in images were color-coded *post hoc* to match the progeny they belong to (see figure key). **Top center:** phase contrast image of the colony at Day 4, with cells color-coded by their generation. **Top right:** glyph representing cell area, generation, relative location in colony, and proliferative capacity of the progeny/progenies that formed the colony (see Fig 4). Filled circles represent individual cells at Day 4, with the size of the circles representing the relative cell spread area and their color representing the generation they belong to. The outer rings represent the number and proliferative capacity of the originating progeny; solid line (–) = fast proliferator, dotted line (…) = moderate proliferator, dashed line (- -) = slow proliferator. Cells that didn’t divide by Day 7 are marked (/). Glyphs were enlarged from Fig 4 for added detail; scale bar is relative only to other glyphs and does not represent an absolute length. **Middle:** lineage trees of colony-originating cells for the first four days of development. Width of lineage lines represents cell spread area at each 15-minute time point. Cells that did not divide by Day 7 and cells whose lifetime differed from their twin more than one standard deviation from the pooled average of all 1384 cells (0.27 days) are indicated (see key). **Bottom:** phase contrast images of the colony at Days 4, 7, and 10. Images underwent a process of background flattening and brightness/contrast adjustment (see Methods). Transparent red dots were placed on the Day 7 phase contrast images for single-cell-derived colonies and mark cells that do not belong to the originating progeny. Scale bars = 250 μm.
(PDF)

S2 Fig Colony confluency, size, and degree of isolation are not indicative of number of originating progenies. **(A)** The confluency of colonies at Day 7, organized by number of originating progenies studied at the single-cell level up to Day 4. In general, single-cell-derived (SCD) colonies tended to be lower in confluency relative to colonies originating from two or more cells, though there was no statistical correlation between colony confluency and number of cells it originated from. **(B)** The approximate diameter of all colonies studied at Day 7 is reported; again, no statistical correlation was found between colony diameter and the number of originating progenies. **(C)** A categorical analysis of the degree of isolation the colonies developed in is presented. Four colonies developed from originating cells that attached relatively close to neighboring cells. In these cases, the initial 1.7 x 1.3 mm montaged field of view (FOV) contained one or more cells close enough to the studied progeny/progenies to be observed at the first time point, yet far enough away that they and their progeny migrated into and out of view and therefore could not be analyzed at the single-cell level. In the next category, neighboring colonies migrated into the FOV of the developing colony within the first two days of development. Many of the colonies were classified into the middle category: no neighboring cells were observed until the FOV was expanded to 2.6 x 2.1 mm at the end of Day 2 (see Methods). In the fourth category, cells not belonging to the originating progeny migrated into the expanded FOV between Days 2 and 4. In the final category, the neighboring cells closest to the developing colony were not revealed until the FOV was again expanded (3.5 x 2.6 mm). **(D)** Boxplots reporting pairwise comparisons of single-cell-derived (SCD) versus multi-cell-derived (MCD) colonies of several properties using a Student’s t-test (see S1 Table for all properties analyzed). Ovals outside of whiskers denote statistical outliers. All measured colony properties differing between MCD and SCD colonies at the p < 0.05 level are shown; however, only one of the several properties measured (the number of asynchronous twin pairs after four days of growth, far right) passed our threshold for significance (Bonferroni-corrected p-value threshold = 0.0005 for 105 pair-wise comparisons). * = p < 0.0005
(TIF)

S3 Fig Further principal component analyses (PCA) demonstrate key biophysical properties driving generational trends, which are not apparent at single time points. **(A)** Coefficient values of principal component 1 (PC1) for the properties analyzed in the PCA presented in Fig 3B. The observed trend in PC1-PC2 space in Fig 3 was not caused by a few properties, but rather a linear combination of many. Properties with the highest coefficient values in positive and negative PC1 space are listed and represent the average measurements over the course of the cells’ lifetimes (e.g., the average value for major axis length of cells over their lifetimes had the highest positive coefficient value for PC1). **(B)** Similarly, the coefficients for PC2 are presented for the PCA in Fig 3B. The top-contributing properties represent the average measurements over the course of the cells’ lifetimes, with the exception of those marked with an asterisk (*), which were properties measured at the time point of each cell’s birth (e.g., the solidity of cells at the time point of their birth had the third-largest contribution in negative PC2 space). **(C)** PCA was conducted on all cells existing at the indicated time points, where the input measurements were properties measured directly by CellProfiler at that time point in the experiment (rather than at time points relative to the cells’ timeline, as in Fig 3). The trend in generation is not apparent in this method of analysis, demonstrating that the biophysical properties of cells, while fundamentally different among cell generations over the course of their lifetimes, cannot indicate the generation or function of cells at any one given time point.
(TIF)

S4 Fig The distribution of cell generation at Day 4 varies by colony and increases with time. **(A)** Average generation of cells for each single-cell-derived (SCD) colony. Error bars represent one standard deviation. **(B)** Average generation of cells for each multi-cell-derived (MCD) colony at Day 4. The number of originating progenies is indicated by color. MCD colonies had statistically higher standard deviations in generation at Day 4 compared to SCD colonies in a t-test (p < 0.001). **(C)** The average cell generation for three SCD colonies analyzed up to Day 6 or 7. The range of generations for each colony increased after Day 4 in all three colonies.
(TIF)

S5 Fig Cell area decreased with each generation, though many cells deviated from this trend. **(A)** Further examples of area-vs-time curves for individual cells are given for several progenies to demonstrate the relationship between cell area, generation (color scheme provided), and senescence (labeled black). The Spearman correlation coefficient (r_s_) and associated p- value for the comparison of average cell area versus generation is also given for each progeny. The top two graphs represent two progenies with a strong decreasing trend in average cell area and generation. The middle two graphs show examples of cells within single progenies that deviate from this trend due to senescence, and the bottom two graphs demonstrate that not all deviating cells were senescent. **(B)** Area vs time for all cells studied, with time labeled relative to the start of the experiment (left) and relative to the birth time point of each cell (right). While a few senescent cells grew to a very large spread area, area alone could not distinguish senescent from proliferating cells.
(TIF)

S6 Fig Analysis of mitotic events in a second colony demonstrates the ability of MSCs to proliferate in highly confluent conditions. The location of each cell division was measured over the course of ten days of colony development and is reported as the location relative to the colony center (left). Colony confluency was also measured at each time point (indicated in the color bar), and a phase contrast image of the colony at Day 10 qualitatively demonstrates the confluent conditions in which mitotic events were detected (right; adjusted for brightness and contrast). Scale bar = 0.5 mm.
(TIF)

S7 Fig Extended single-cell analysis of three single-cell-derived colonies demonstrates consistent heterogeneity onset within putative stem cell colonies. Three single-cell-derived (SCD) colonies (Colonies A, L, and P) were analyzed at the single-cell level up to Day 6 or Day 7. **(A, F, K):** Lineage trees for each SCD colony. Lineage lines are color-coded by cell generation, and the width of the lines represents the area of the cell at each 15-minute time point. **(B, G, L):** Glyphs representing cell area, generation, location, and proliferative capacity of their progeny at fixed time points (see Fig 4 for detailed key). **(C, H, M):** Boxplots of cell area, categorized by cell generation. Circles outside of whiskers represent statistical outliers. Cell area was calculated as the average area of each cell over all 15-minute time points for the first 0.83 days of their lifetimes. One outlier data point in panel M (5^th^ generation, average area = 7,288 μm^2^) was removed from this figure for scaling purposes. **(D, I, N):** Area-vs-time curves for each cell over the course of analysis. Data line color represents cell generation, and the vertical dashed line indicates the time point at which cells were fed. **(E, J, O):** Principal component analysis (PCA) of all cells belonging to the colony over the course of analysis. Input variables were averaged values of 54 properties measured by CellProfiler (see Methods), and data point colors represent cell generation.
(PDF)

S8 Fig Time lapse video of a single-cell-derived colony for approximately fourteen days of development. This time lapse video was constructed using MATLAB at 10 frames per second, where each frame corresponds to one 15 min time point.
(AVI)

S9 Fig Time lapse video of a multi-cell-derived colony for approximately fourteen days of development. This time lapse video was constructed using MATLAB at 10 frames per second, where each frame corresponds to one 15 min time point.
(AVI)

S10 Fig Time lapse video of one single-cell-derived colony with color-coded cells. This time lapse video was constructed using MATLAB at 10 frames per second, where each frame corresponds to one 15 min time point. Images were altered post-experiment to color code the cells by cell generation.
(MP4)

S1 Table Spearman correlation coefficients (r_S_) and associated p values for colony-wide properties. Correlations passing thresholds for each are highlighted.
(XLSX)

S2 Table Spearman correlation coefficients (r_S_) and associated p values for single-cell properties of all pooled cells analyzed. Correlations passing thresholds for p value and critical values of the Spearman correlation coefficient are highlighted in green. p values which pass the Bonferroni-corrected p value threshold for significance, but whose corresponding correlation coefficient does not pass the threshold for significance, are highlighted in blue and may possibly represent weak correlations by less-stringent criteria.
(XLSX)
