# Supplementary material for: Emergent heterogeneity in putative mesenchymal stem cell colonies: Single-cell time lapsed analysis
Source: PLoS One. 2019 Apr 3;14(4):e0213452. doi: 10.1371/journal.pone.0213452 (PMC6447157; doi:10.1371/journal.pone.0213452)
Supplement: S1 Fig — Top left: phase contrast image of the colony at after four days of growth. Cells in images were color-coded post hoc to match the progeny they belong to (see figure key). Top center: phase contrast image of the colony at Day 4, with cells color-coded by their generation. Top right: glyph representing cell area, generation, relative location in colony, and proliferative capacity of the progeny/progenies that formed the colony (see Fig 4). Filled circles represent individual cells at Day 4, with the size of the circles representing the relative cell spread area and their color representing the generation they belong to. The outer rings represent the number and proliferative capacity of the originating progeny; solid line (–) = fast proliferator, dotted line (…) = moderate proliferator, dashed line (- -) = slow proliferator. Cells that didn’t divide by Day 7 are marked (/). Glyphs were enlarged from Fig 4 for added detail; scale bar is relative only to other glyphs and does not represent an absolute length. Middle: lineage trees of colony-originating cells for the first four days of development. Width of lineage lines represents cell spread area at each 15-minute time point. Cells that did not divide by Day 7 and cells whose lifetime differed from their twin more than one standard deviation from the pooled average of all 1384 cells (0.27 days) are indicated (see key). Bottom: phase contrast images of the colony at Days 4, 7, and 10. Images underwent a process of background flattening and brightness/contrast adjustment (see Methods). Transparent red dots were placed on the Day 7 phase contrast images for single-cell-derived colonies and mark cells that do not belong to the originating progeny. Scale bars = 250 μm. (PDF) [file pone.0213452.s001.pdf]

Figure S1: In-depth profiles of every colony studied at the single cell level. Top left: phase contrast image of the colony at after four days of growth. Cells in images were color-coded *post hoc* to match the progeny they belong to (see figure key). **Top center:** phase contrast image of the colony at Day 4, with cells color-coded by their generation. **Top right:** glyph representing cell area, generation, relative location in colony, and proliferative capacity of the progeny/progenies that formed the colony (see Figure 4). Filled circles represent individual cells at Day 4, with the size of the circles representing the relative cell spread area and their color representing the generation they belong to. The outer rings represent the number and proliferative capacity of the originating progeny; solid line (–) = fast proliferator, dotted line (…) = moderate proliferator, dashed line (- -) = slow proliferator. Cells that didn't divide by Day 10 are marked (/). Glyphs were enlarged from Figure 4 for added detail; scale bar is relative only to other glyphs and does not represent an absolute length. **Middle:** lineage trees of colony-originating cells for the first four days of development. Width of lineage lines represents cell spread area at each 15-minute time point. Cells that did not divide by Day 10 and cells whose lifetime differed from their twin more than one standard deviation from the pooled average of all 1384 cells (0.27 days) are indicated (see key). **Bottom:** phase contrast images of the colony at Days 4, 7, and 10. Images underwent a process of background flattening and brightness/contrast adjustment (see methods). Transparent red dots were placed on the Day 7 phase contrast images for single-cell-derived colonies and mark cells that do not belong to the originating progeny. Scale bars = 250 µm.

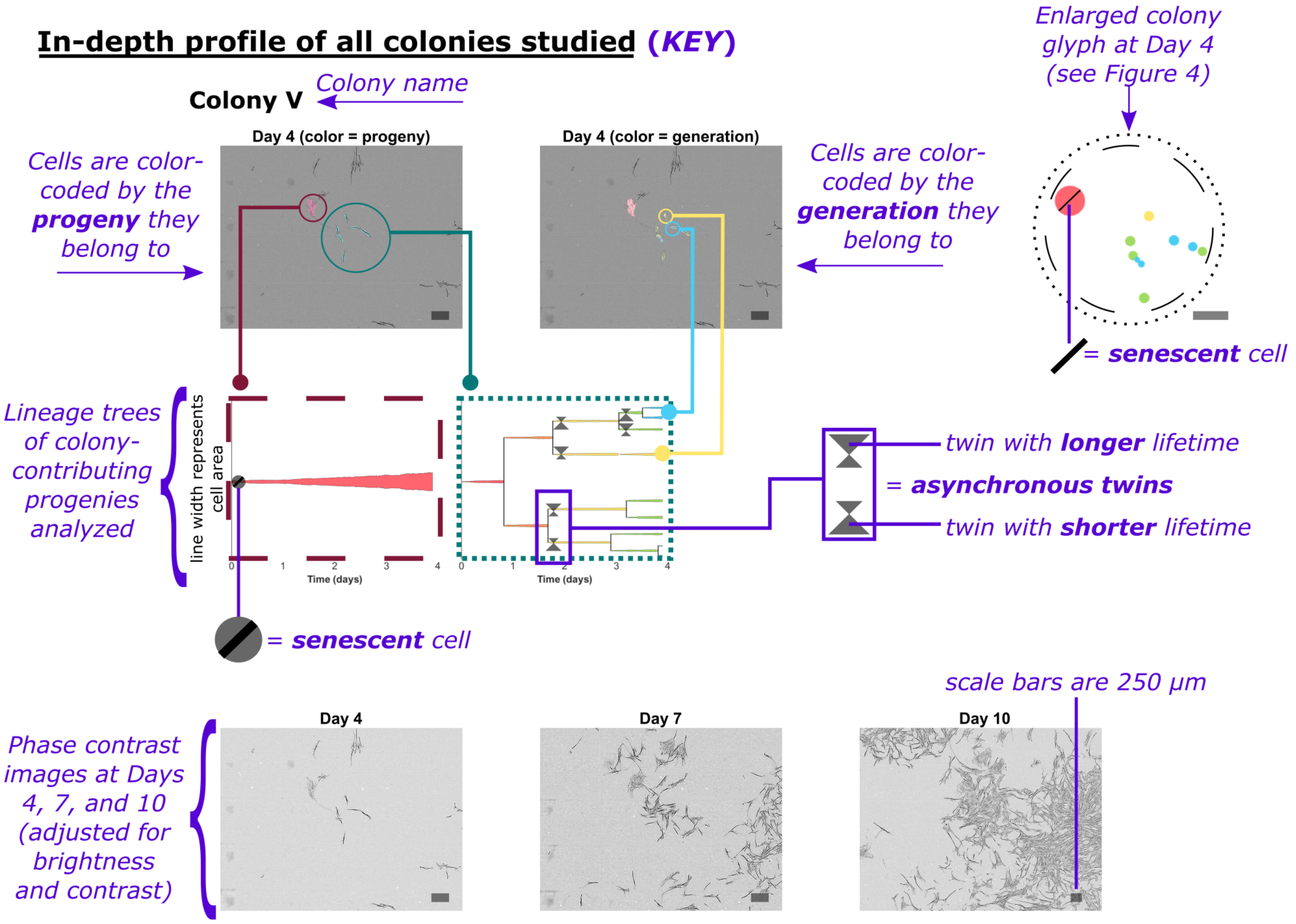
In-depth profile of all colonies studied (KEY)
Colony V
Colony name
Enlarged colony glyph at Day 4 (see Figure 4)
Day 4 (color = progeny)
Day 4 (color = generation)
Cells are color-coded by the progeny they belong to
Cells are color-coded by the generation they belong to
= senescent cell
Lineage trees of colony-contributing progenies analyzed
line width represents cell area
0
1
2
3
4
Time (days)
twin with longer lifetime
= asynchronous twins
twin with shorter lifetime
= senescent cell
scale bars are 250 μm
Phase contrast images at Days 4, 7, and 10 (adjusted for brightness and contrast)
Day 4
Day 7
Day 10

Colony A

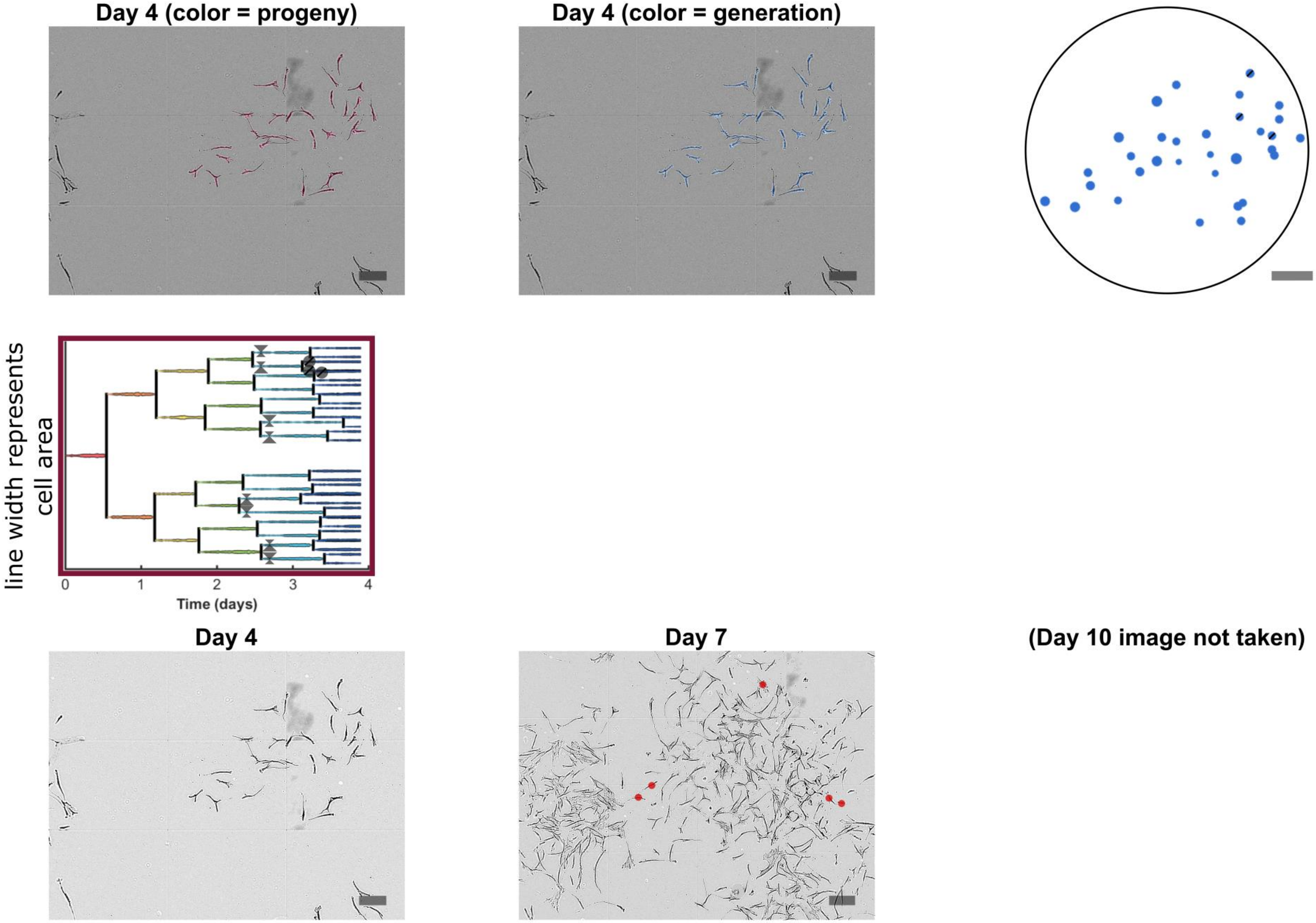

Day 4 (color = progeny)
Day 4 (color = generation)
line width represents cell area
0
1
2
3
4
Time (days)
Day 4
Day 7
(Day 10 image not taken)

Colony B

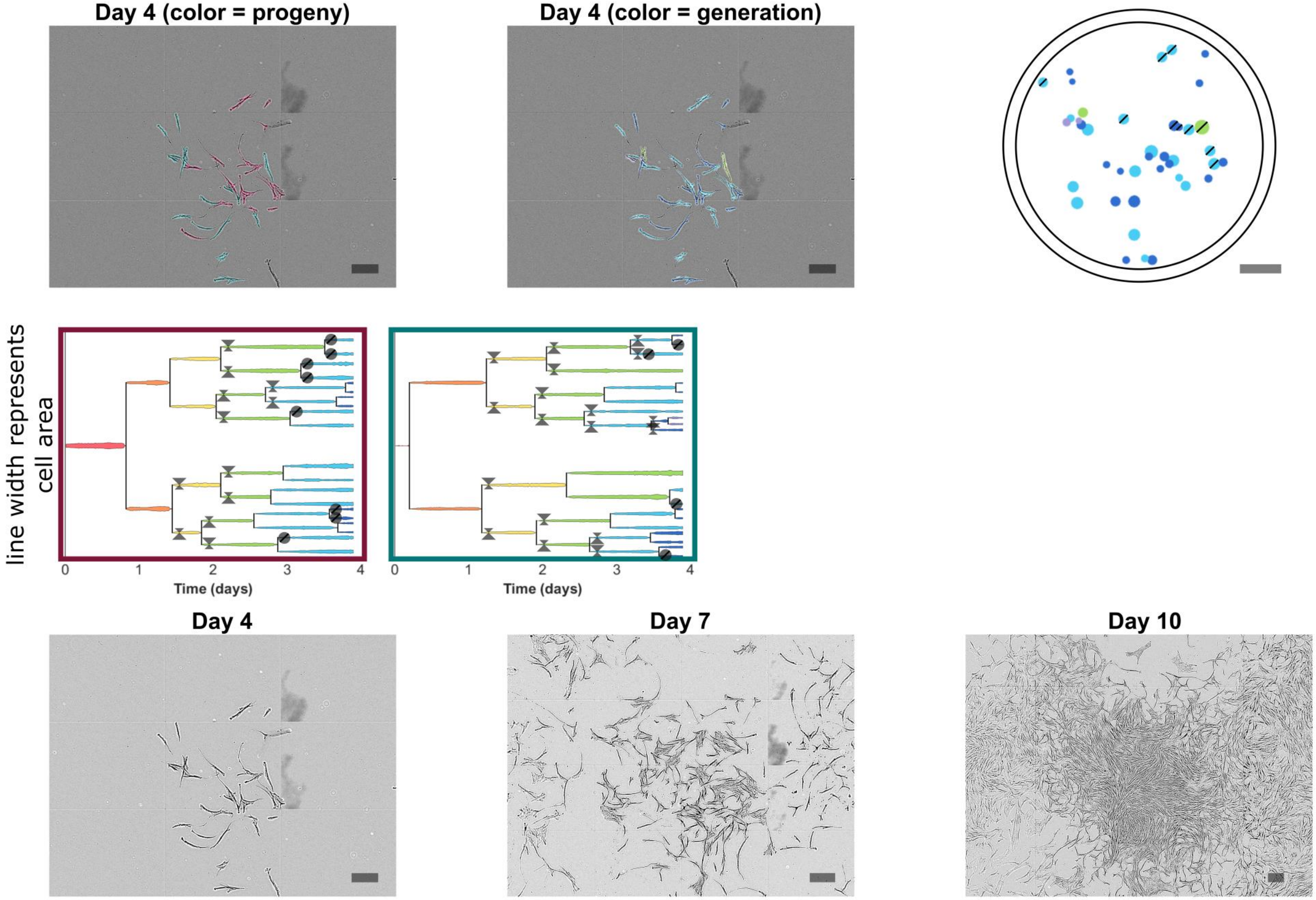

Day 4 (color = progeny)
Day 4 (color = generation)
line width represents
cell area
0
1
2
3
4
Time (days)
0
1
2
3
4
Time (days)
Day 4
Day 7
Day 10

Colony C

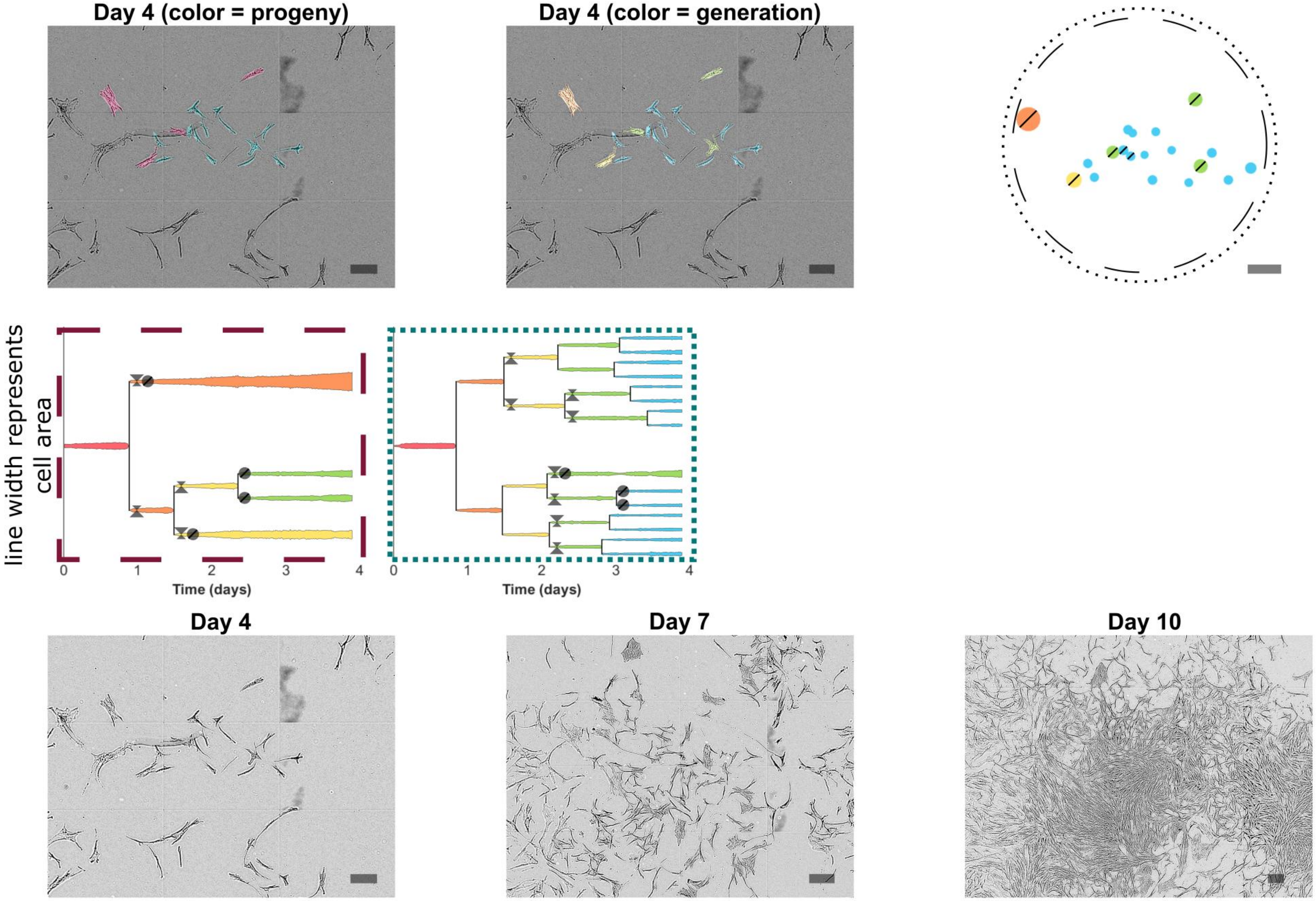

Day 4 (color = progeny)
Day 4 (color = generation)
line width represents cell area
Time (days)
0
1
2
3
4
Time (days)
0
1
2
3
4
Day 4
Day 7
Day 10

Colony D

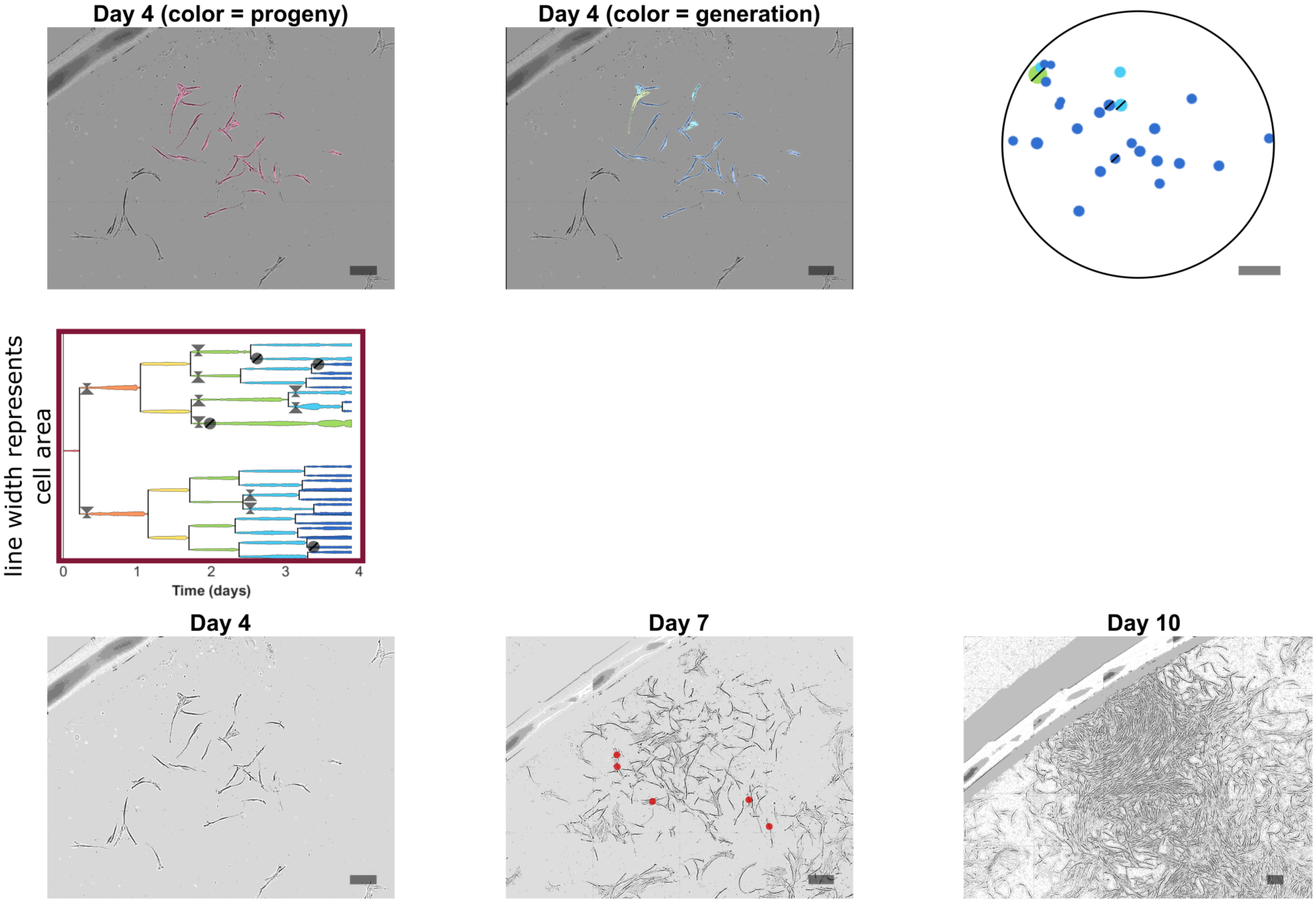

Day 4 (color = progeny)
Day 4 (color = generation)
line width represents cell area
0
1
2
3
4
Time (days)
Day 4
Day 7
Day 10

Colony E

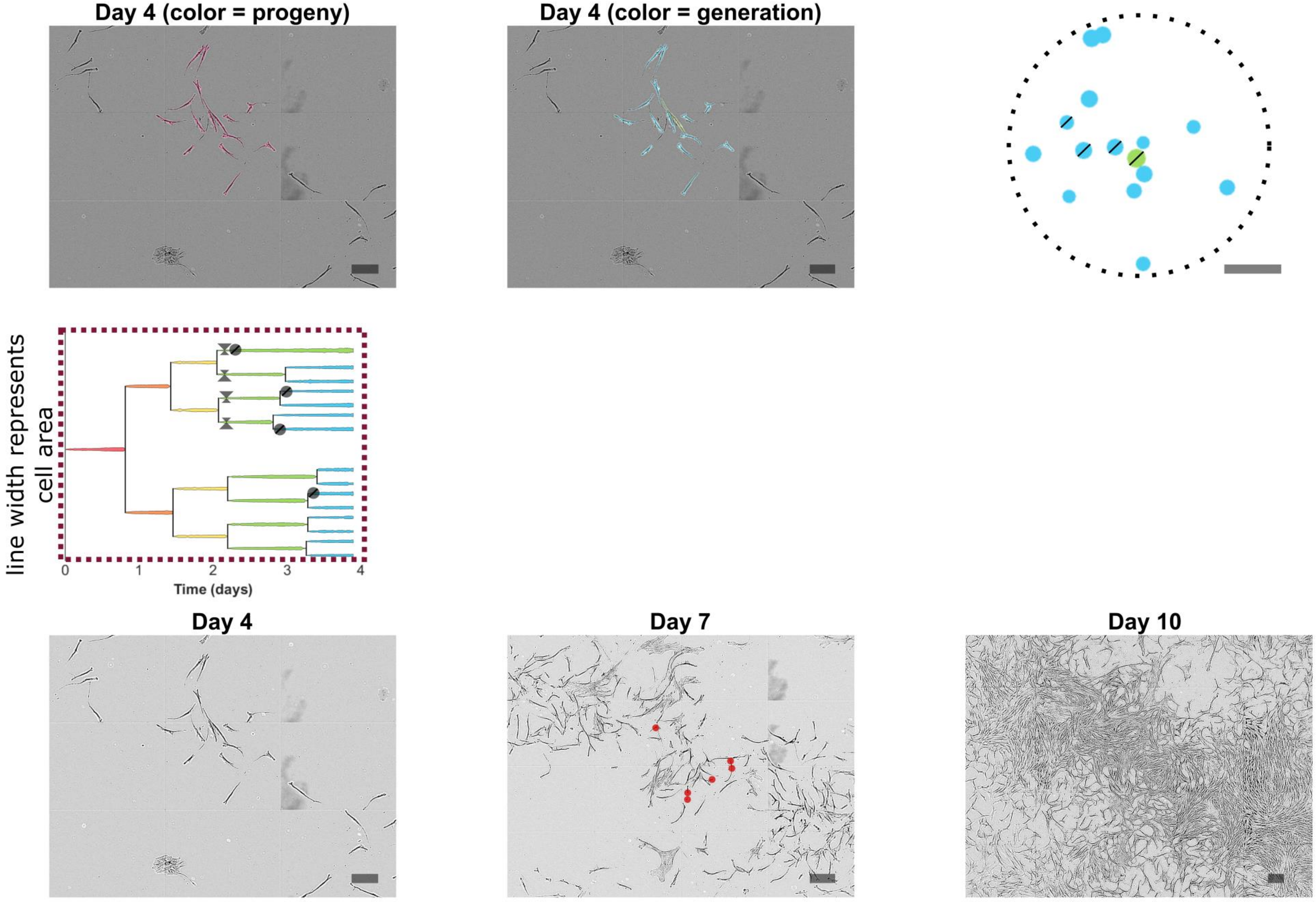

Day 4 (color = progeny)
Day 4 (color = generation)
line width represents cell area
0
1
2
3
4
Time (days)
Day 4
Day 7
Day 10

Colony F

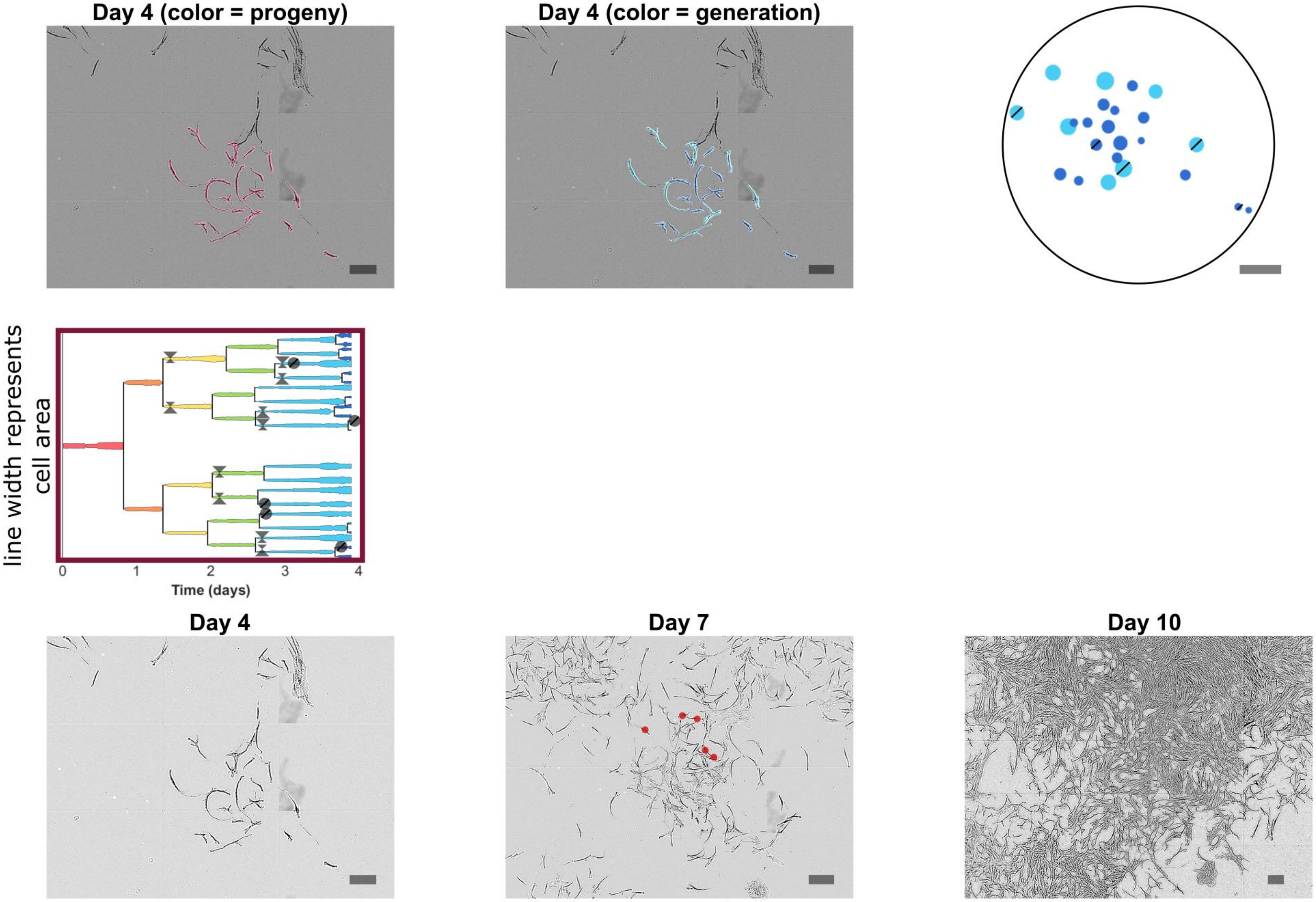
Day 4 (color = progeny)
Day 4 (color = generation)
line width represents cell area
0
1
2
3
4
Time (days)
Day 4
Day 7
Day 10

Colony G

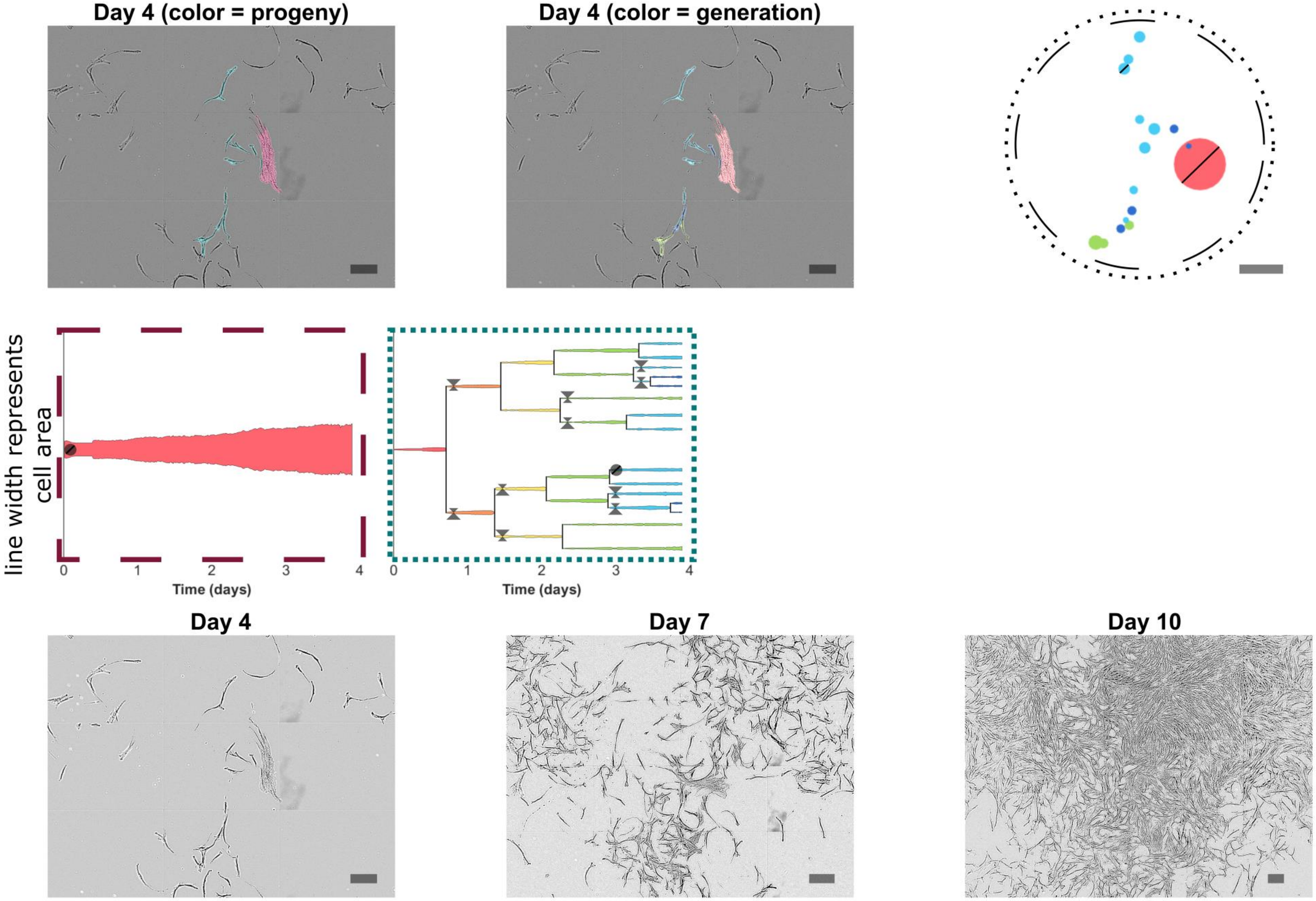

Day 4 (color = progeny)
Day 4 (color = generation)
line width represents cell area
0
1
2
3
4
Time (days)
0
1
2
3
4
Time (days)
Day 4
Day 7
Day 10

Colony H

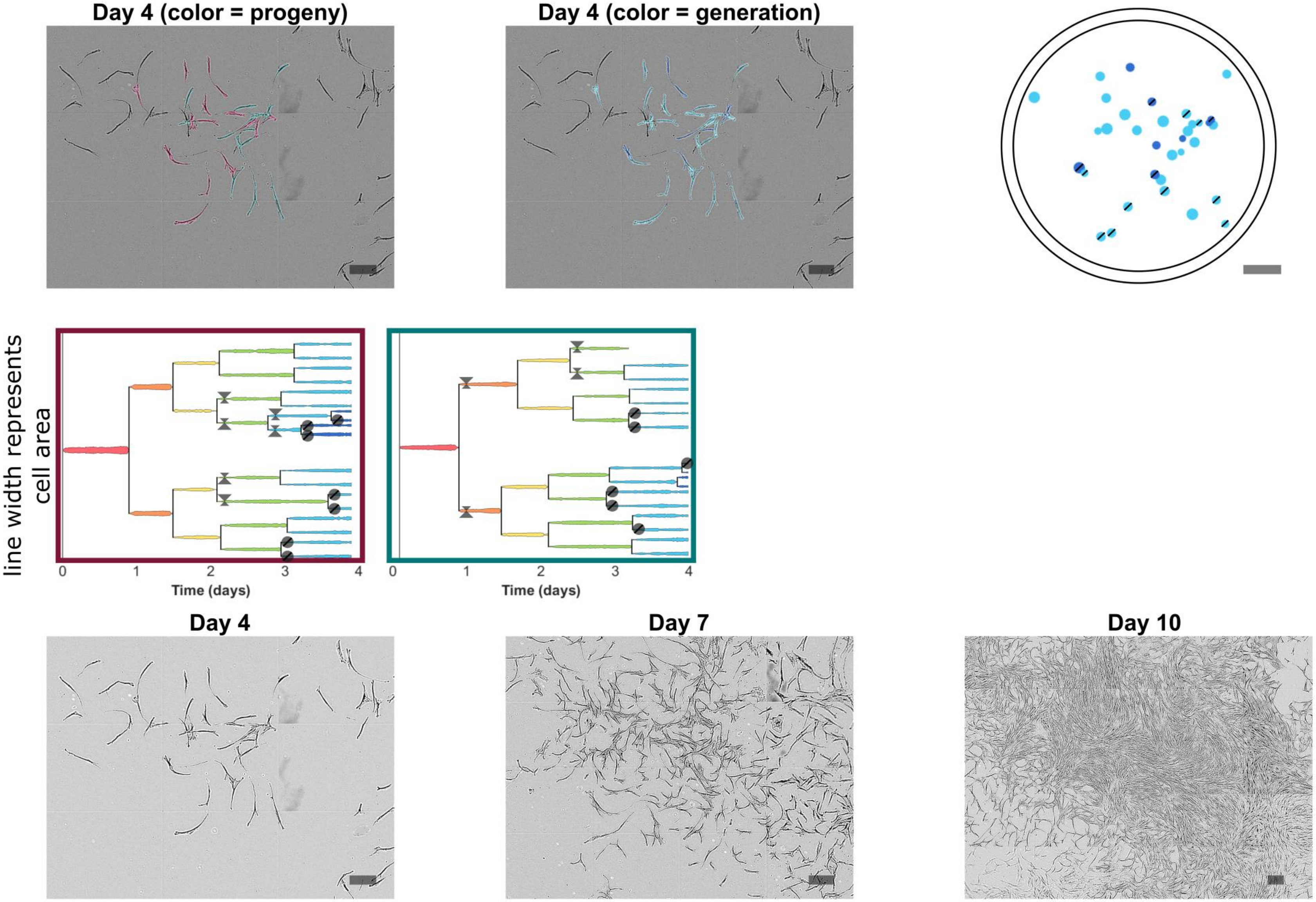

Day 4 (color = progeny)
Day 4 (color = generation)
line width represents cell area
0
1
2
3
4
Time (days)
0
1
2
3
4
Time (days)
Day 4
Day 7
Day 10

Colony I

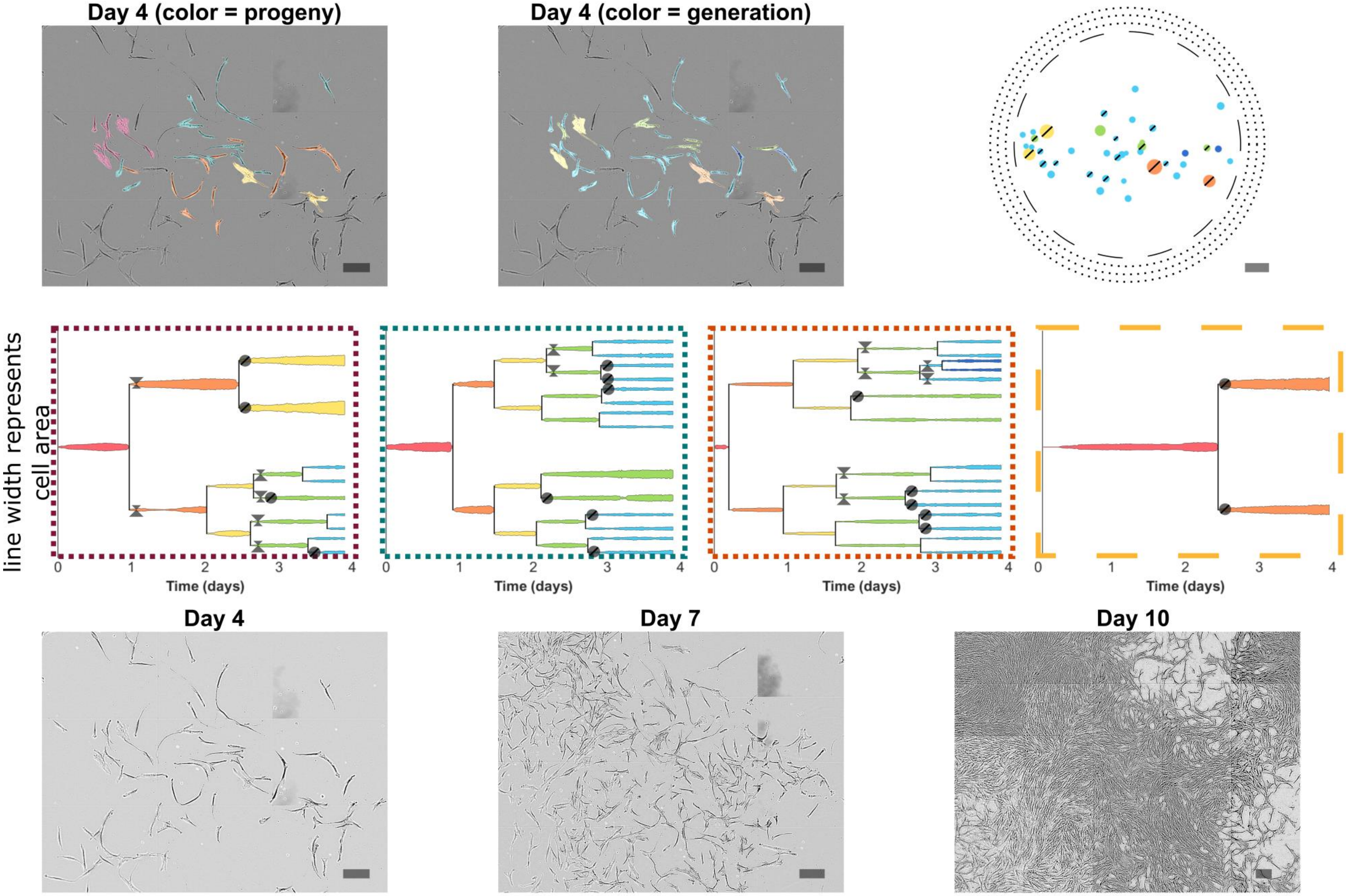
Day 4 (color = progeny)
Day 4 (color = generation)
line width represents cell area
0
1
2
3
4
Time (days)
Day 4
Day 7
Day 10

Colony J

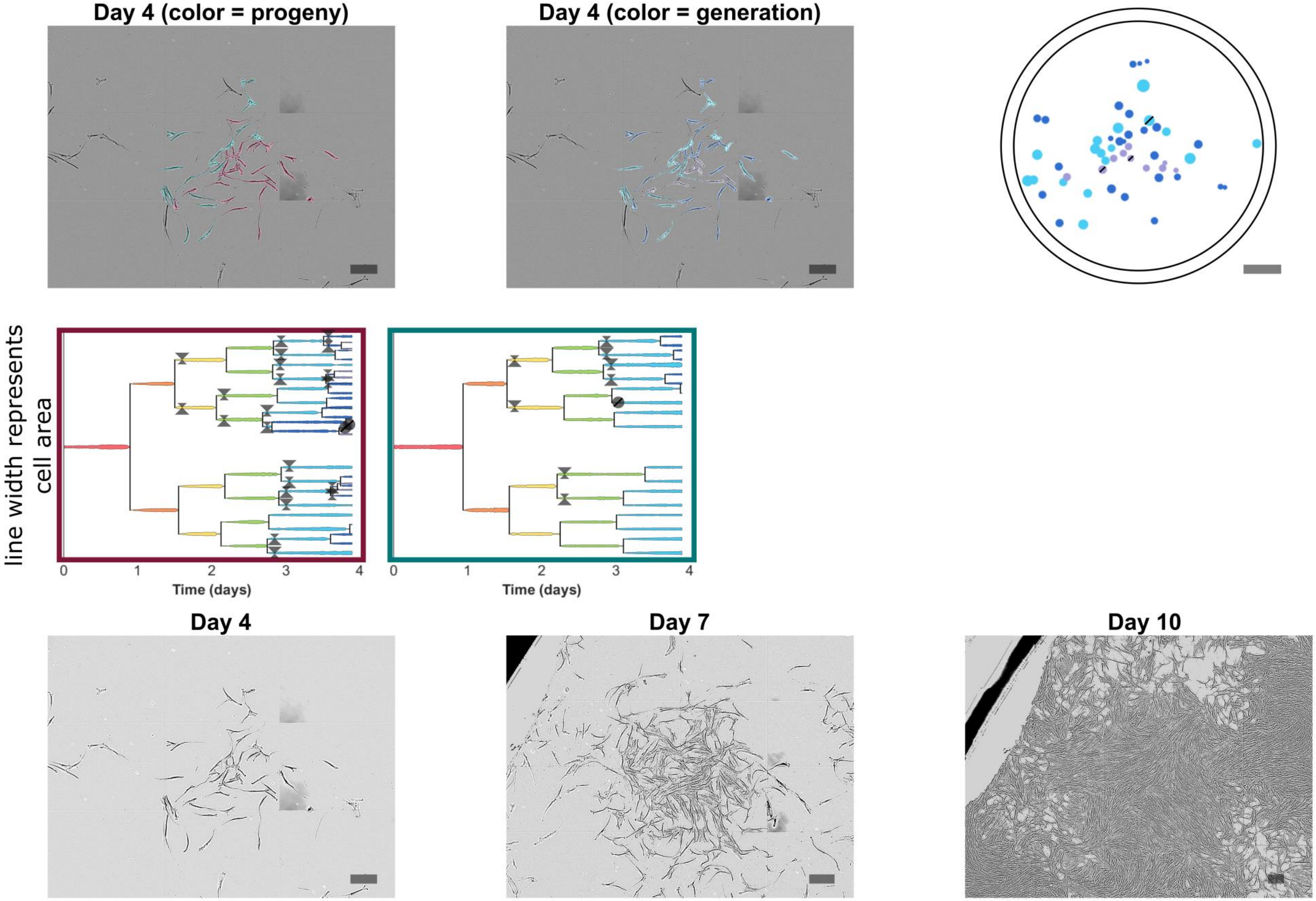
Day 4 (color = progeny)
Day 4 (color = generation)
line width represents cell area
0
1
2
3
4
Time (days)
0
1
2
3
4
Time (days)
Day 4
Day 7
Day 10

Colony K

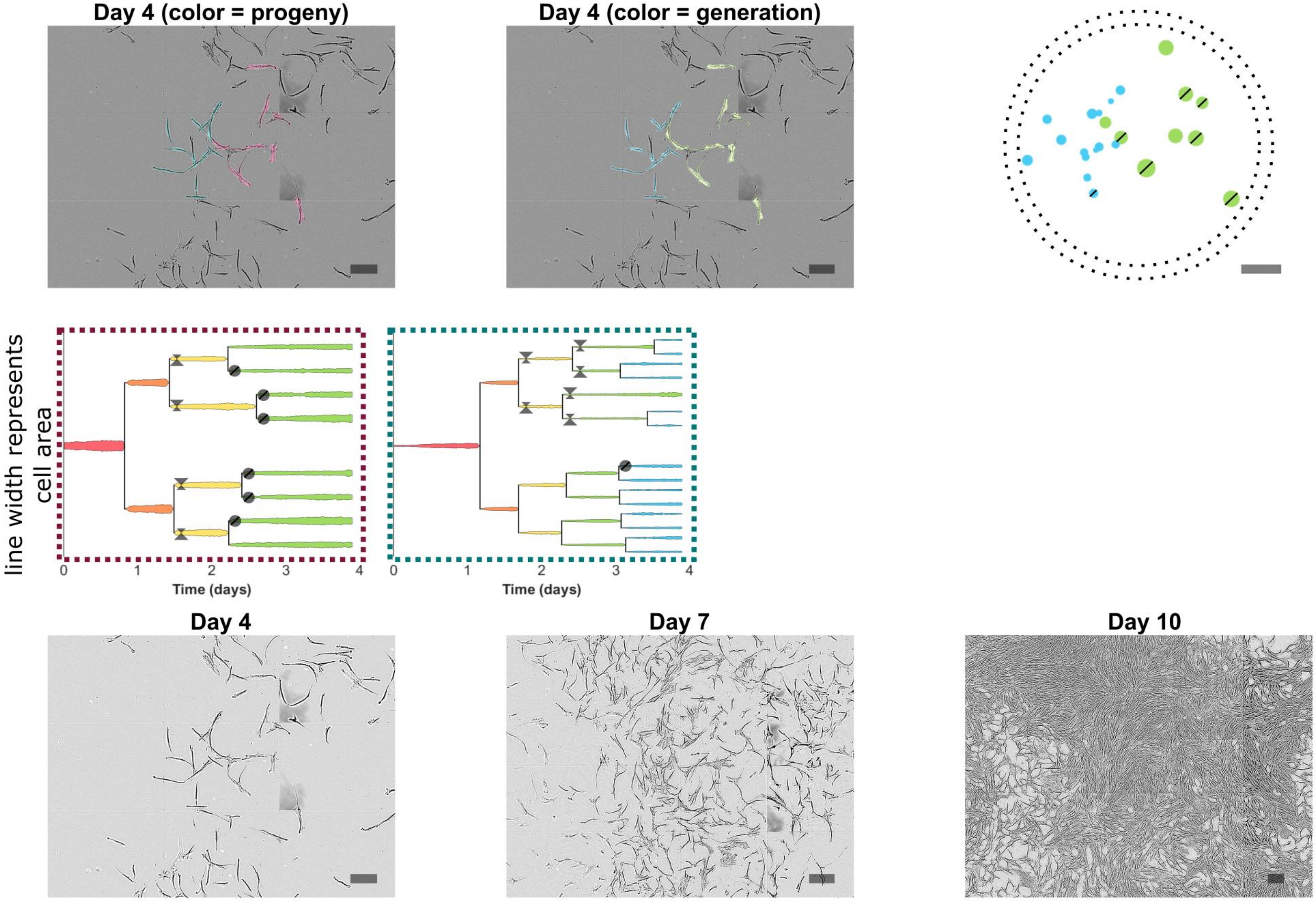

Day 4 (color = progeny)
Day 4 (color = generation)
line width represents
cell area
0
1
2
3
4
Time (days)
0
1
2
3
4
Time (days)
Day 4
Day 7
Day 10

Colony L

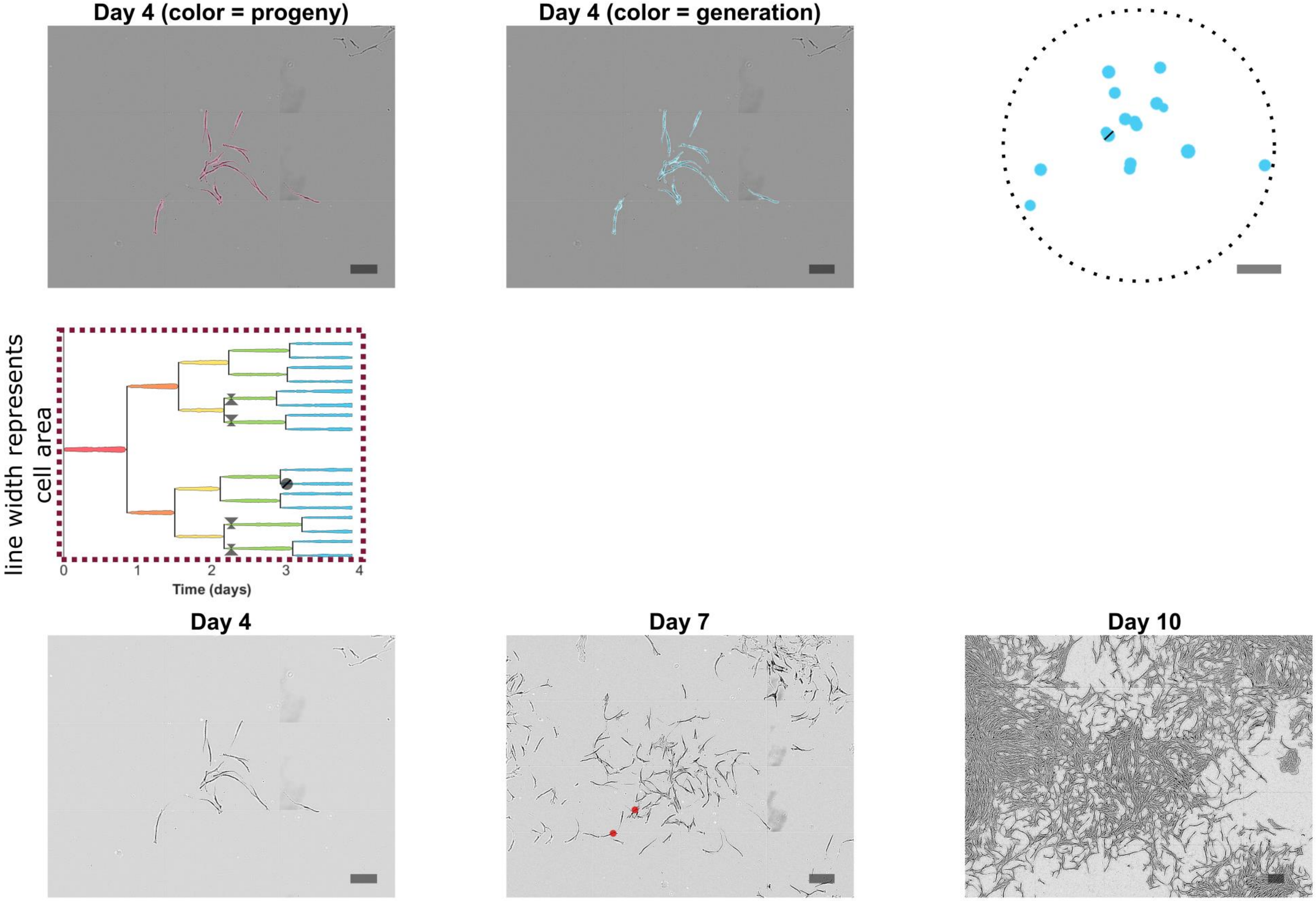
Day 4 (color = progeny)
Day 4 (color = generation)
line width represents
cell area
0
1
2
3
4
Time (days)
Day 4
Day 7
Day 10

Colony M

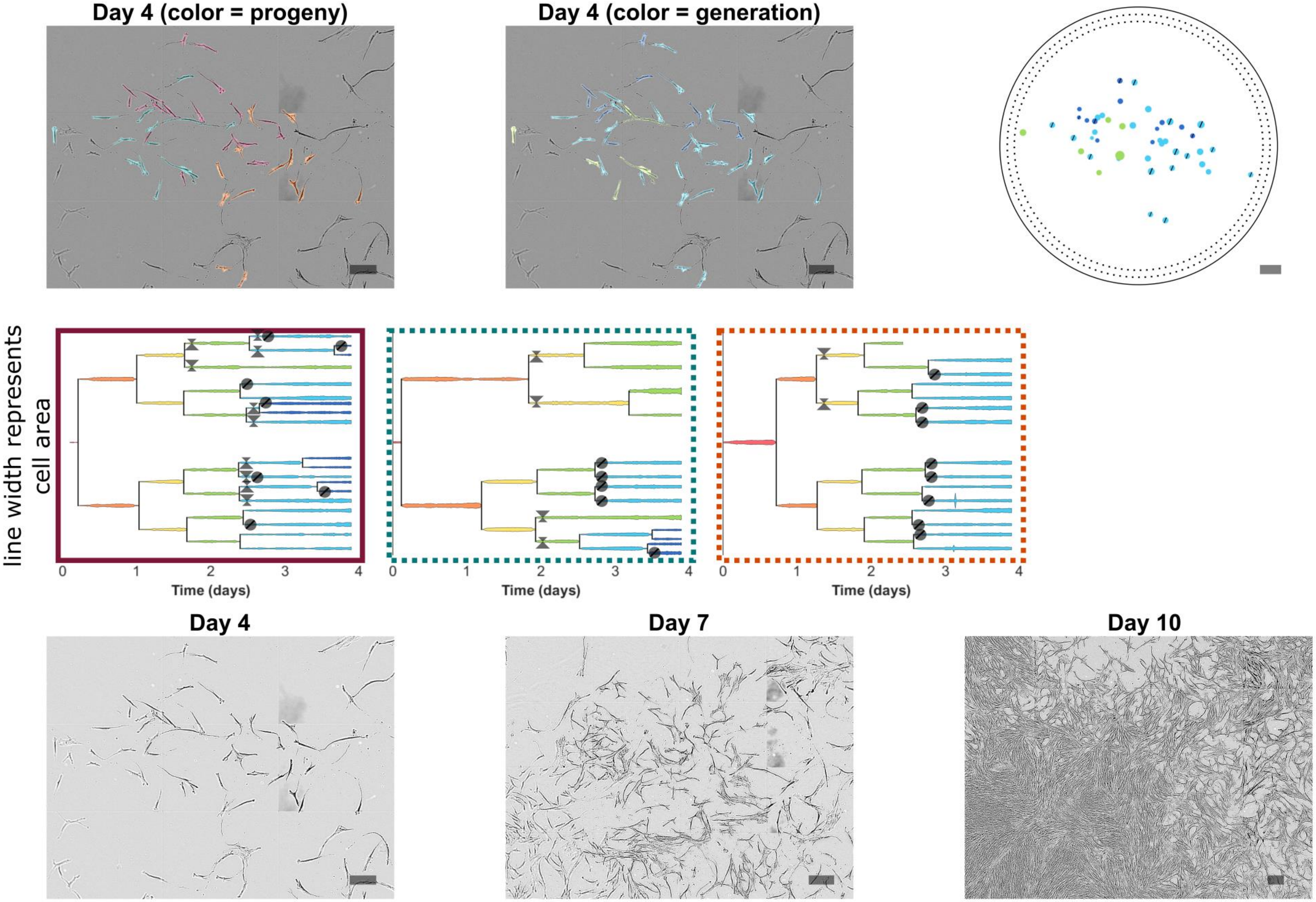

Day 4 (color = progeny)
Day 4 (color = generation)
line width represents cell area
0
1
2
3
4
Time (days)
0
1
2
3
4
Time (days)
0
1
2
3
4
Time (days)
Day 4
Day 7
Day 10

Colony N

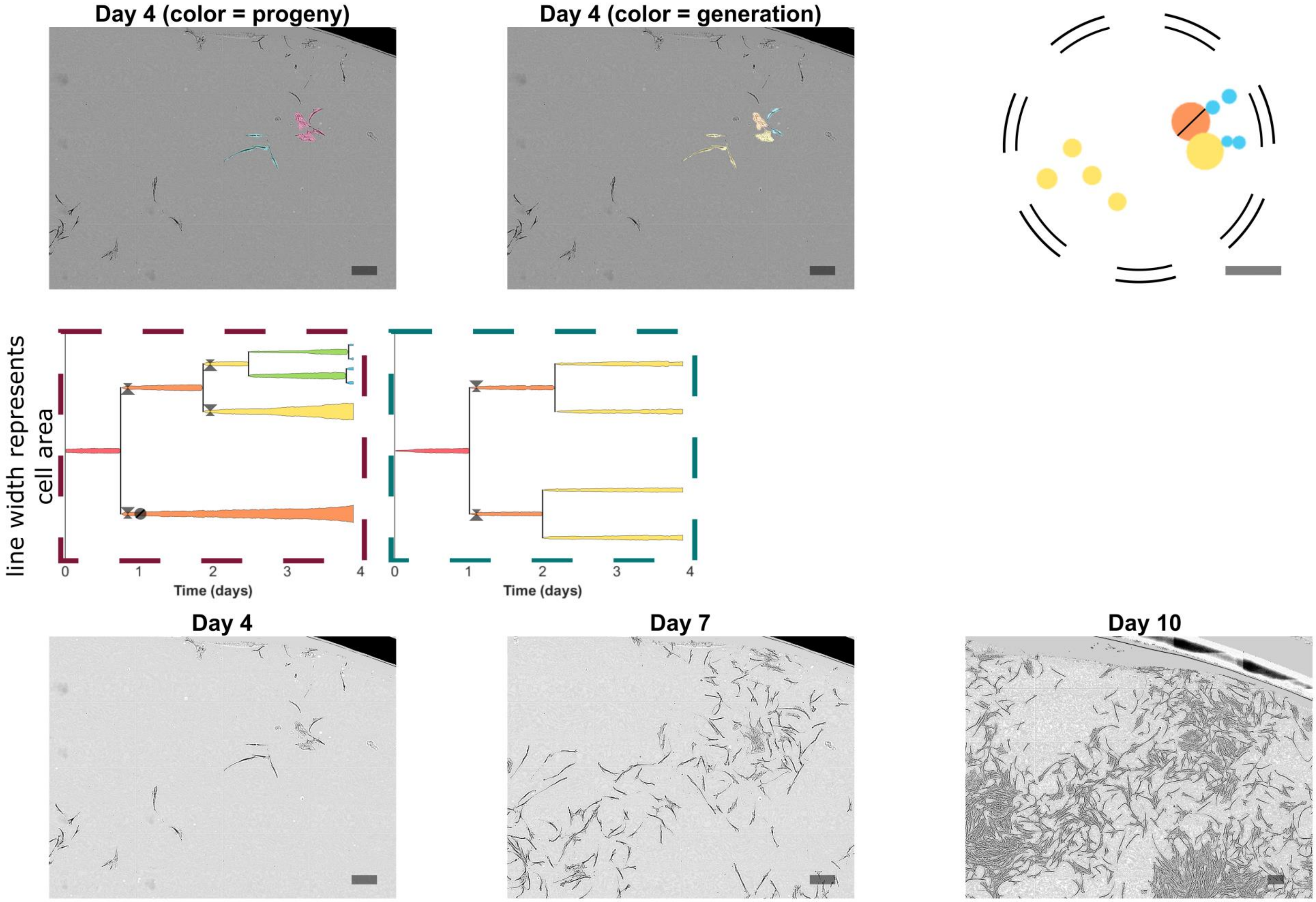

Day 4 (color = progeny)
Day 4 (color = generation)
line width represents cell area
0
1
2
3
4
Time (days)
0
1
2
3
4
Time (days)
Day 4
Day 7
Day 10

Colony O1

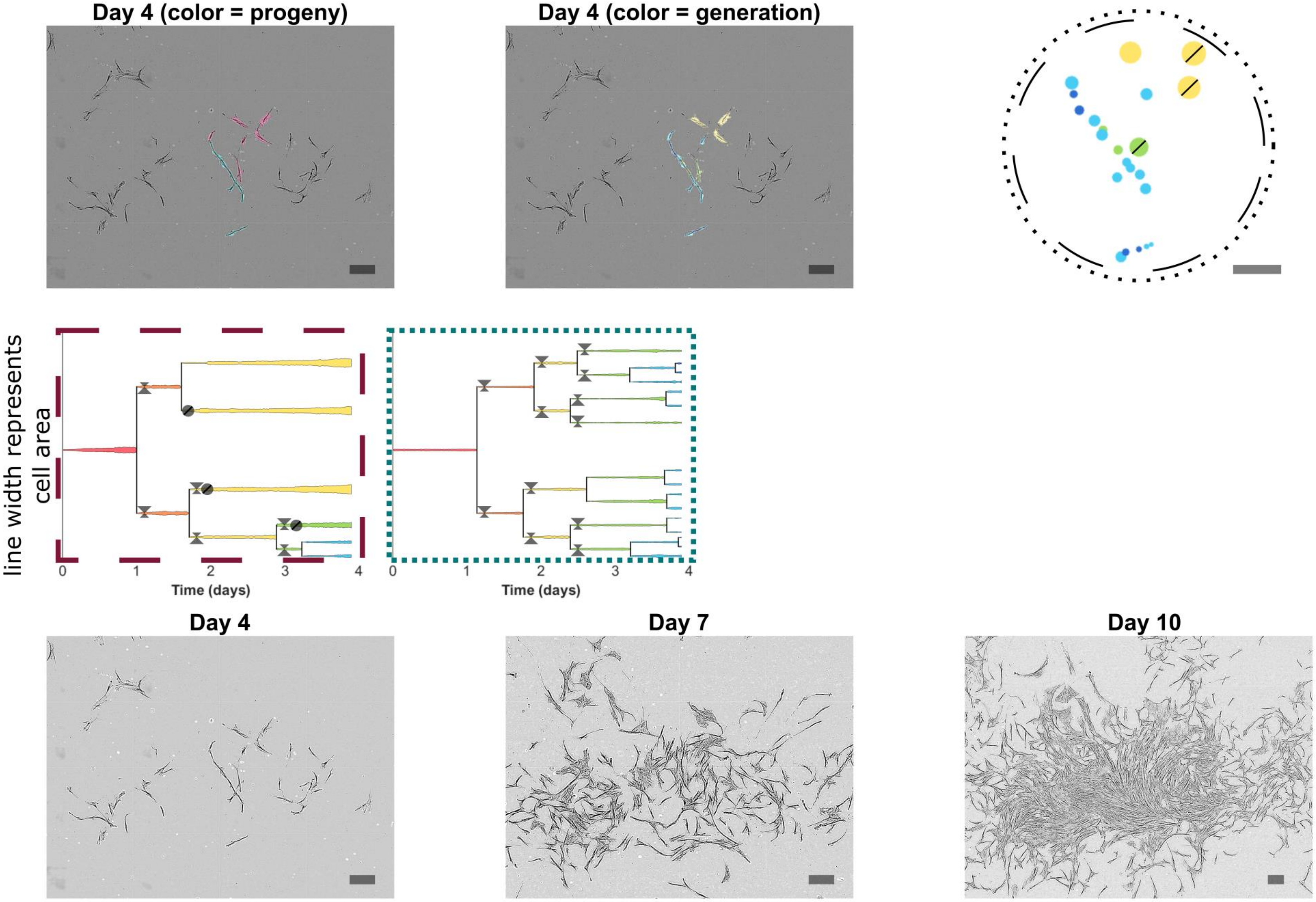
Day 4 (color = progeny)
Day 4 (color = generation)
line width represents cell area
0
1
2
3
4
Time (days)
0
1
2
3
4
Time (days)
Day 4
Day 7
Day 10

Colony O2

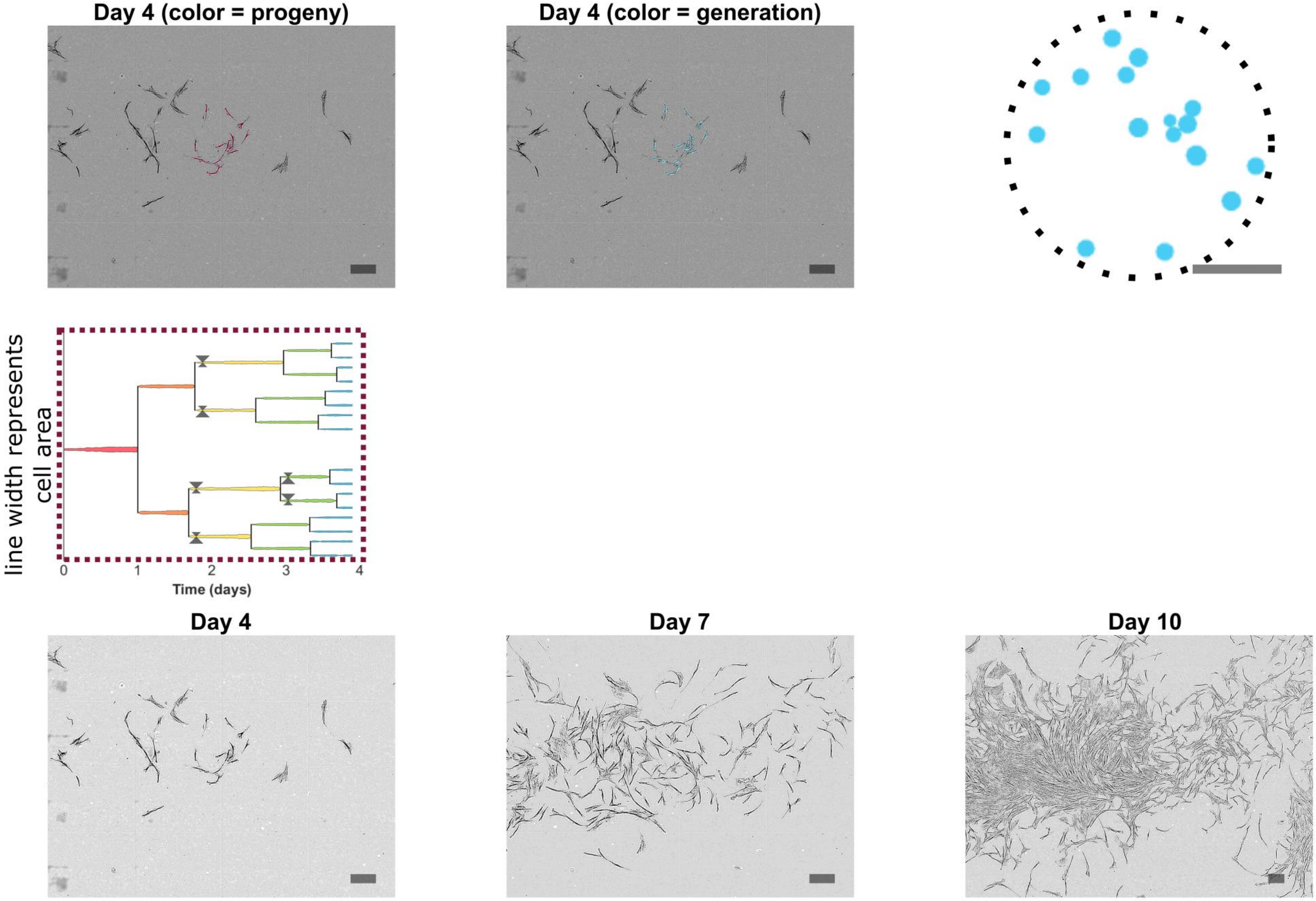
Day 4 (color = progeny)
Day 4 (color = generation)
line width represents cell area
0
1
2
3
4
Time (days)
Day 4
Day 7
Day 10

Colony P

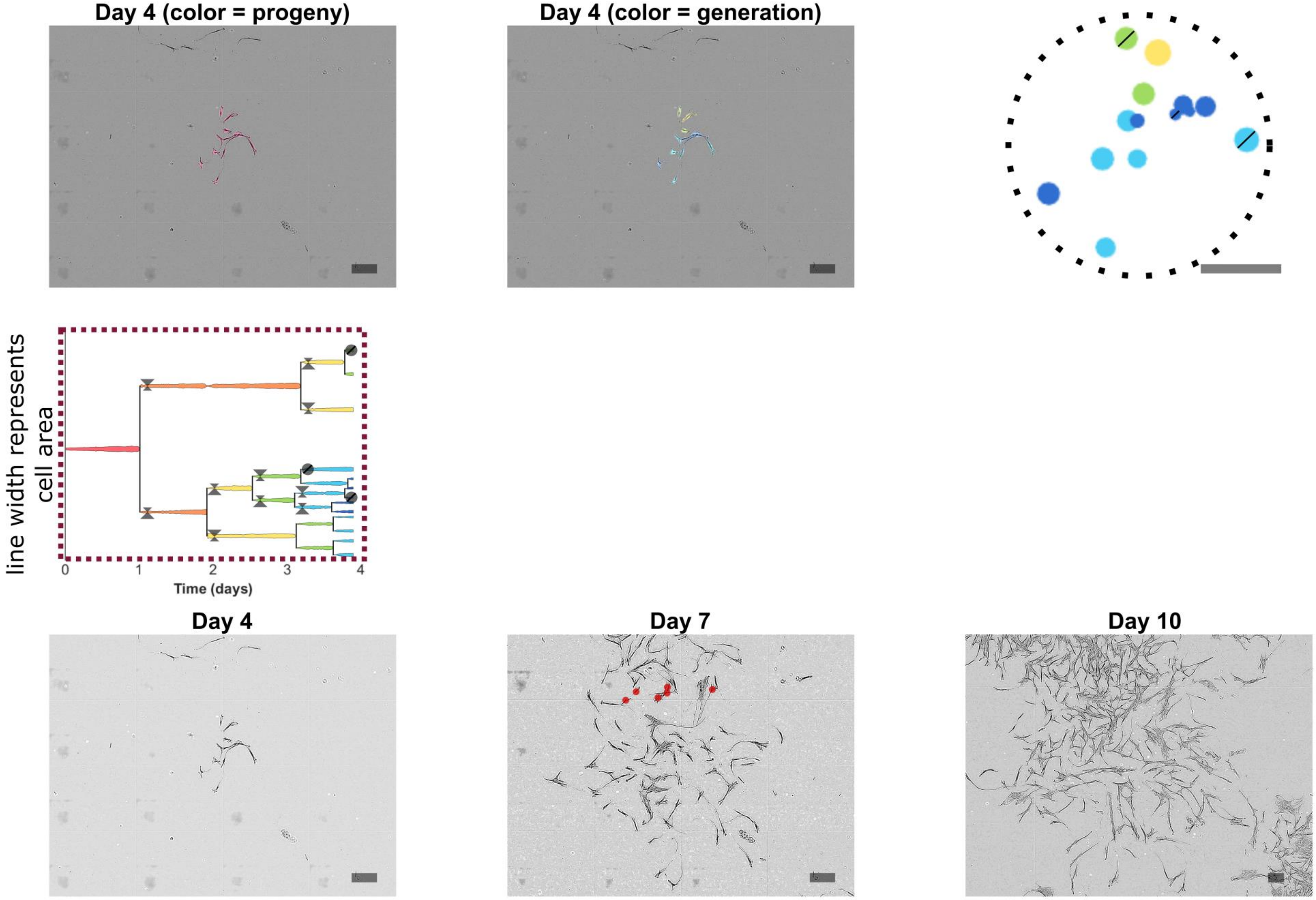

Day 4 (color = progeny)
Day 4 (color = generation)
line width represents
cell area
0
1
2
3
4
Time (days)
Day 4
Day 7
Day 10

Colony Q1

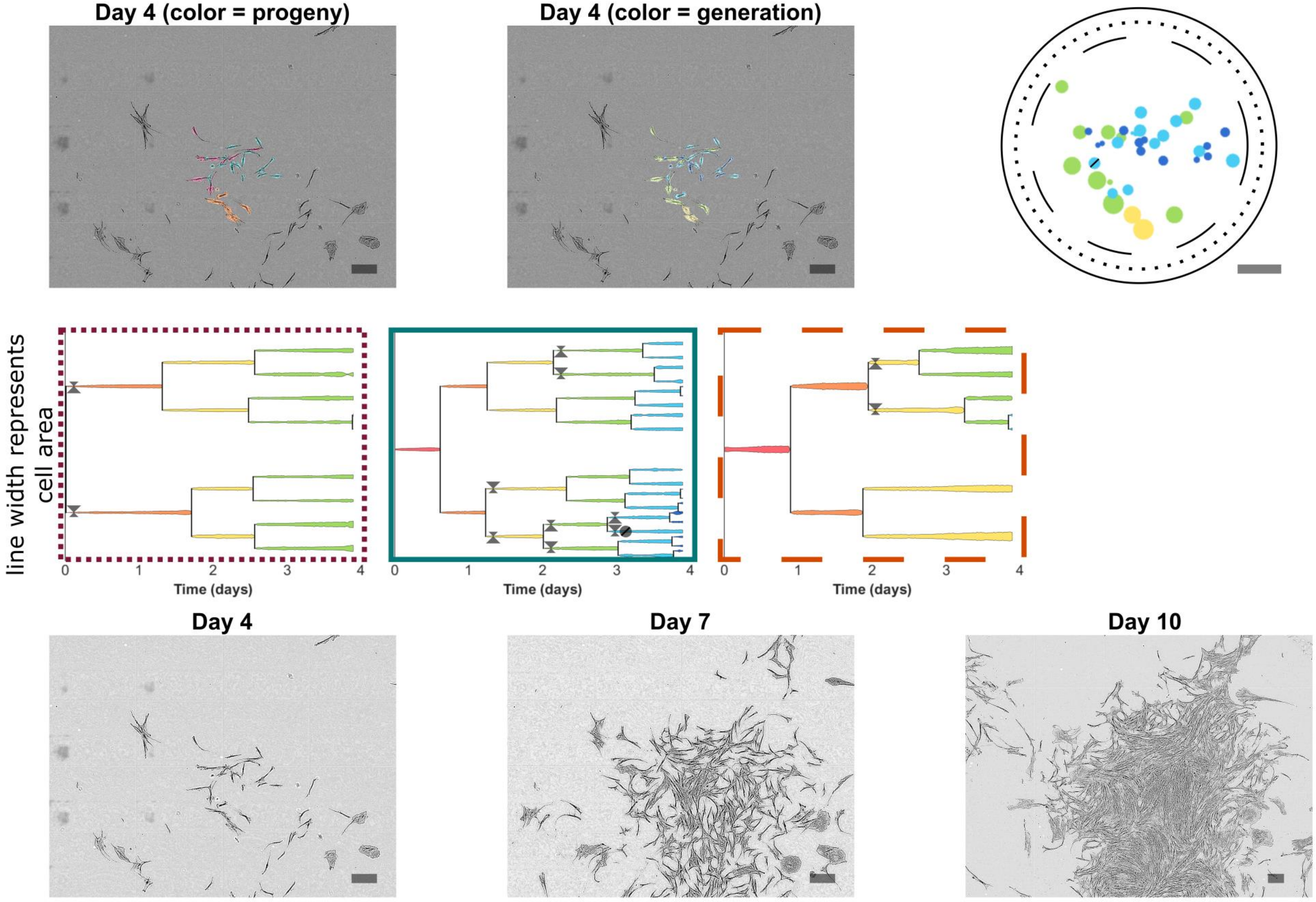

Day 4 (color = progeny)
Day 4 (color = generation)
line width represents cell area
0
1
2
3
4
Time (days)
Day 4
Day 7
Day 10

Colony Q2

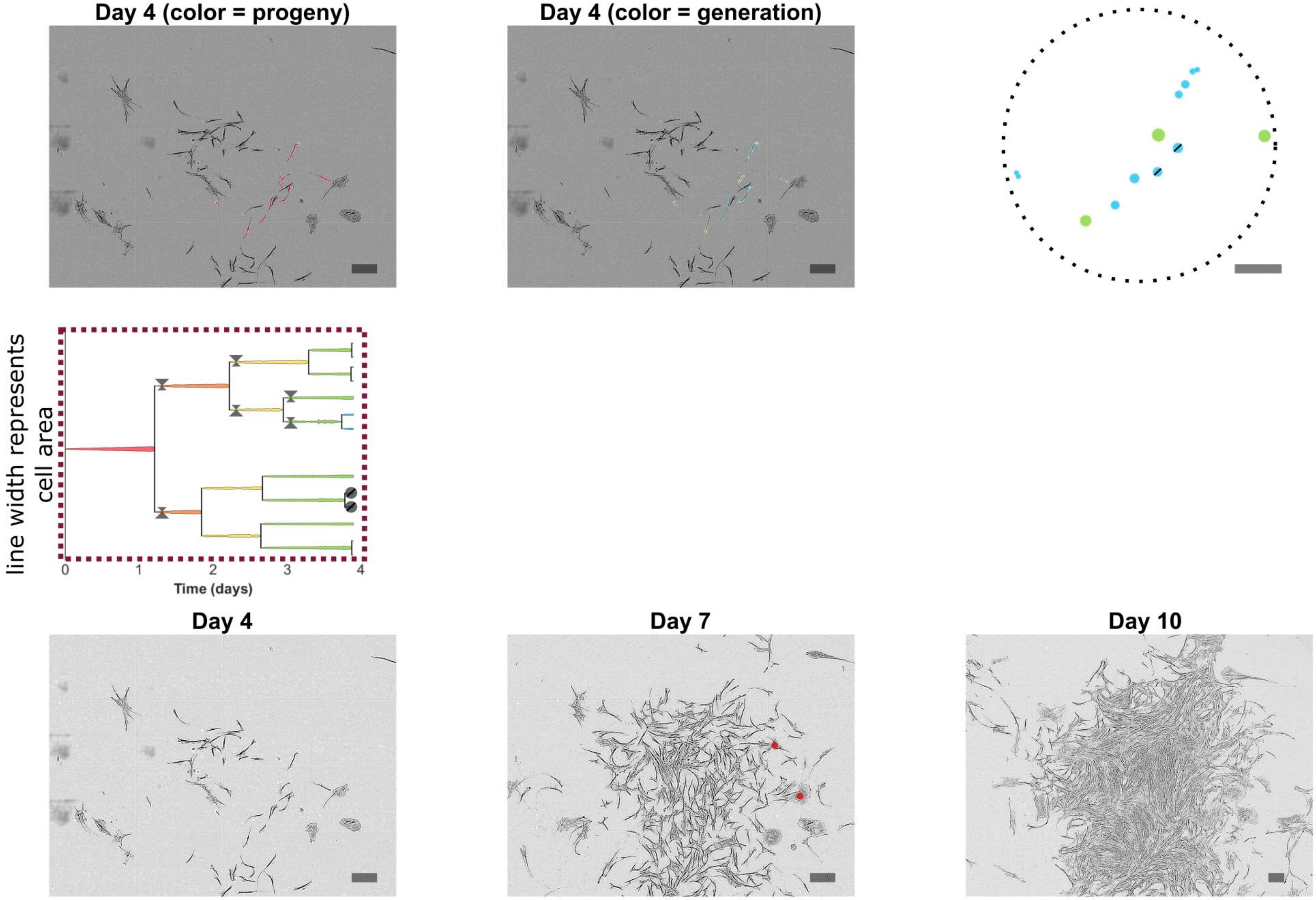

Day 4 (color = progeny)
Day 4 (color = generation)
line width represents cell area
0
1
2
3
4
Time (days)
Day 4
Day 7
Day 10

Colony R

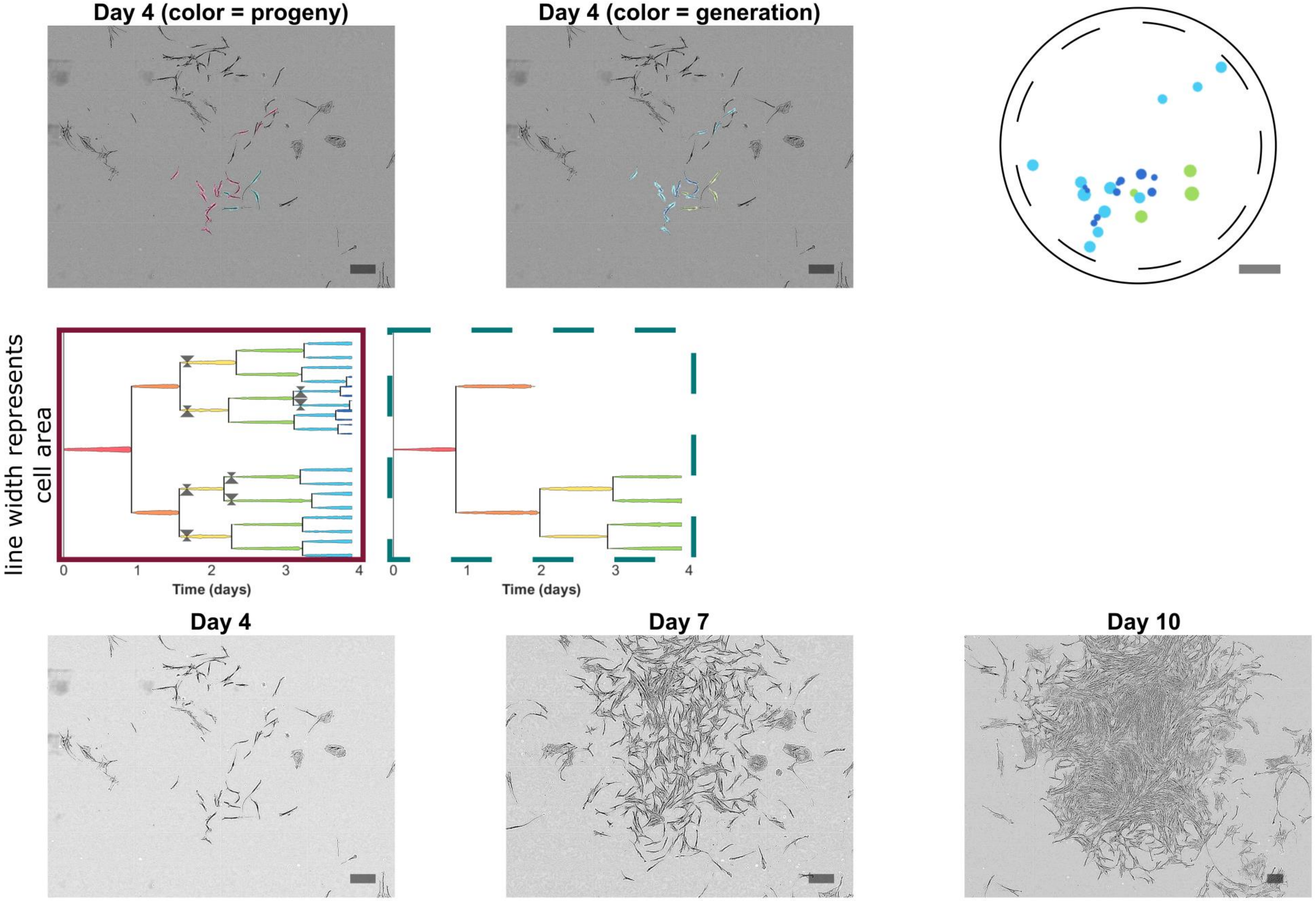
Day 4 (color = progeny)
Day 4 (color = generation)
line width represents cell area
0
1
2
3
4
Time (days)
0
1
2
3
4
Time (days)
Day 4
Day 7
Day 10

Colony S

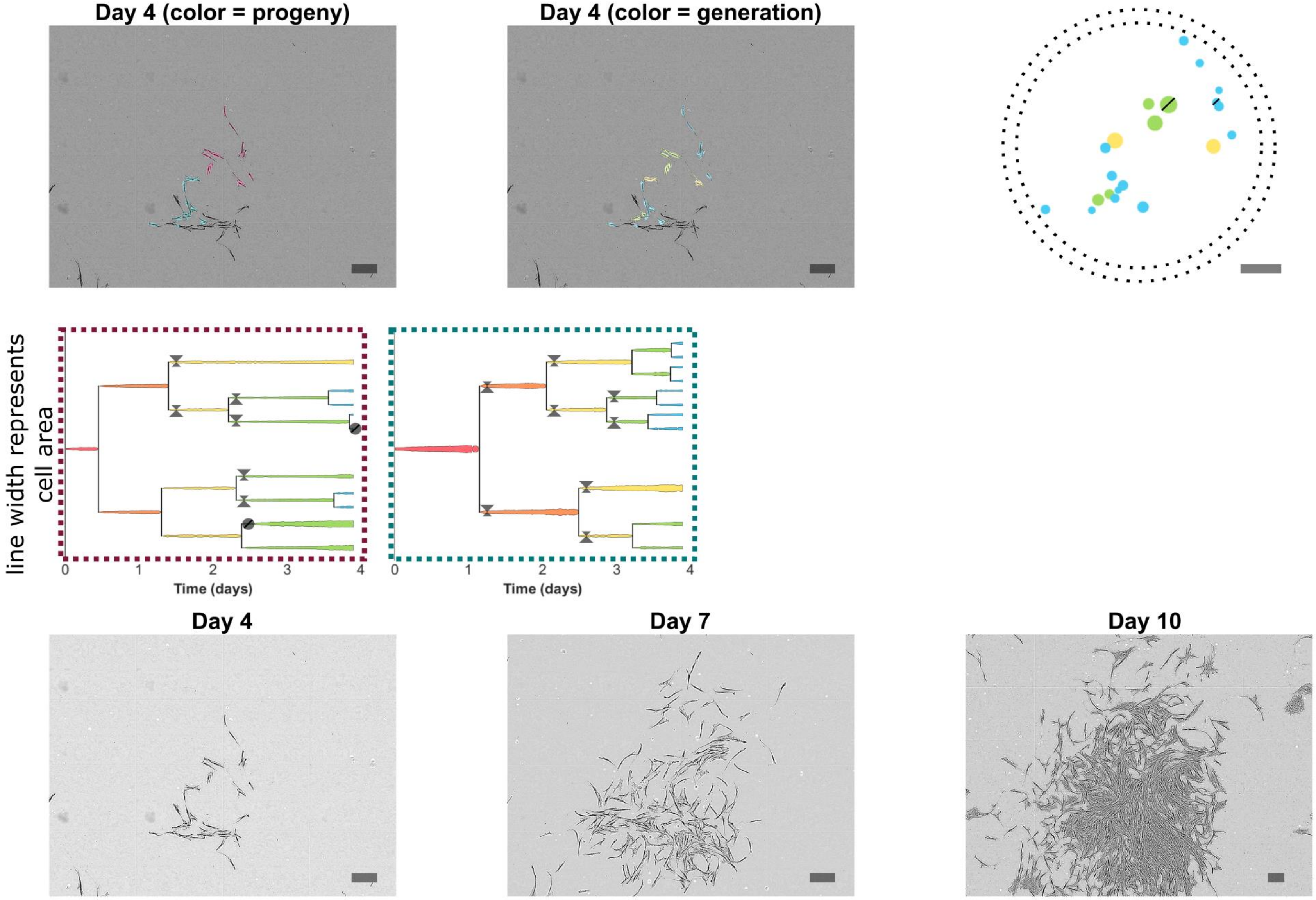

Day 4 (color = progeny)
Day 4 (color = generation)
line width represents cell area
0
1
2
3
4
Time (days)
0
1
2
3
4
Time (days)
Day 4
Day 7
Day 10

Colony T

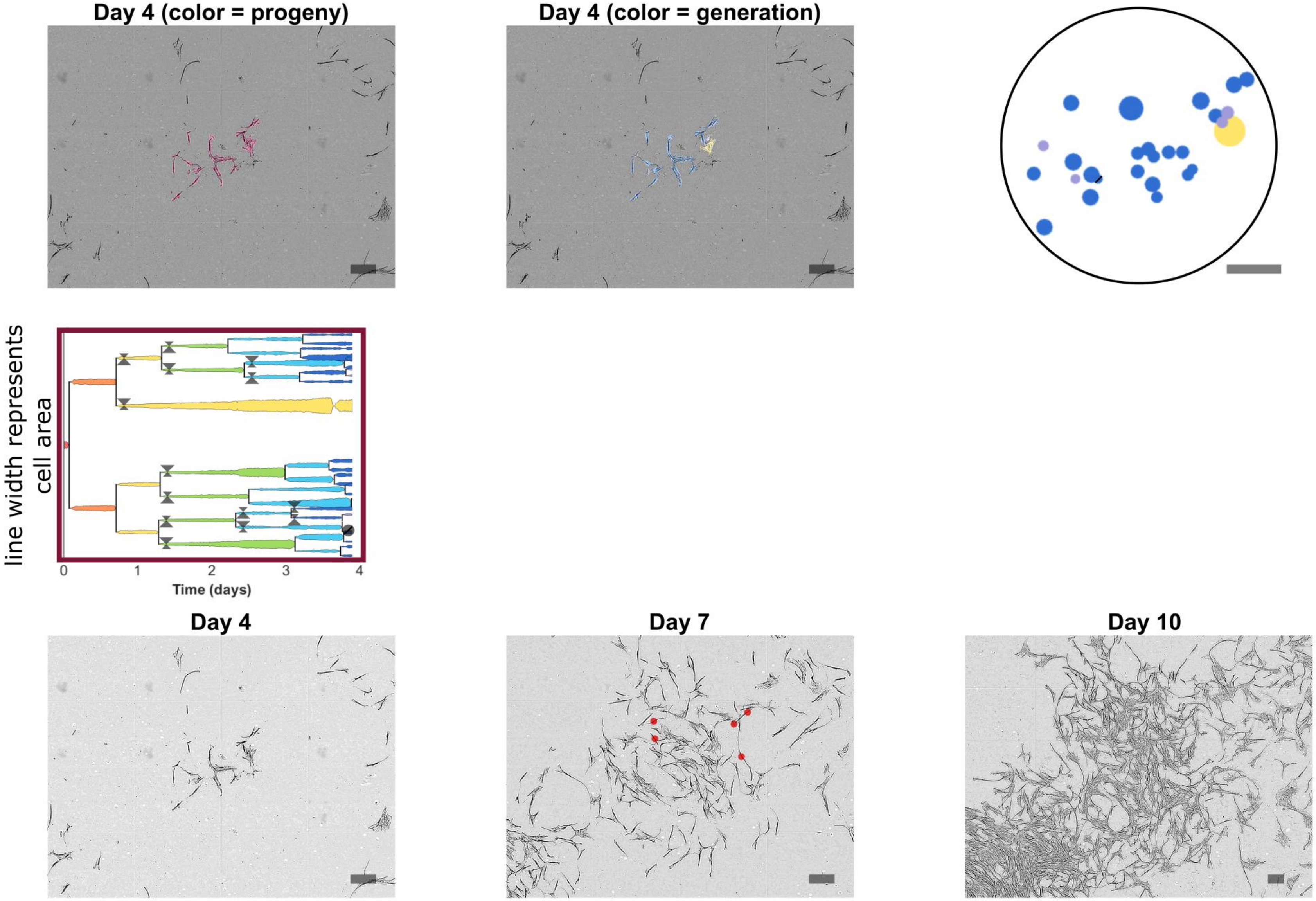

Day 4 (color = progeny)
Day 4 (color = generation)
line width represents cell area
0
1
2
3
4
Time (days)
Day 4
Day 7
Day 10

Colony U

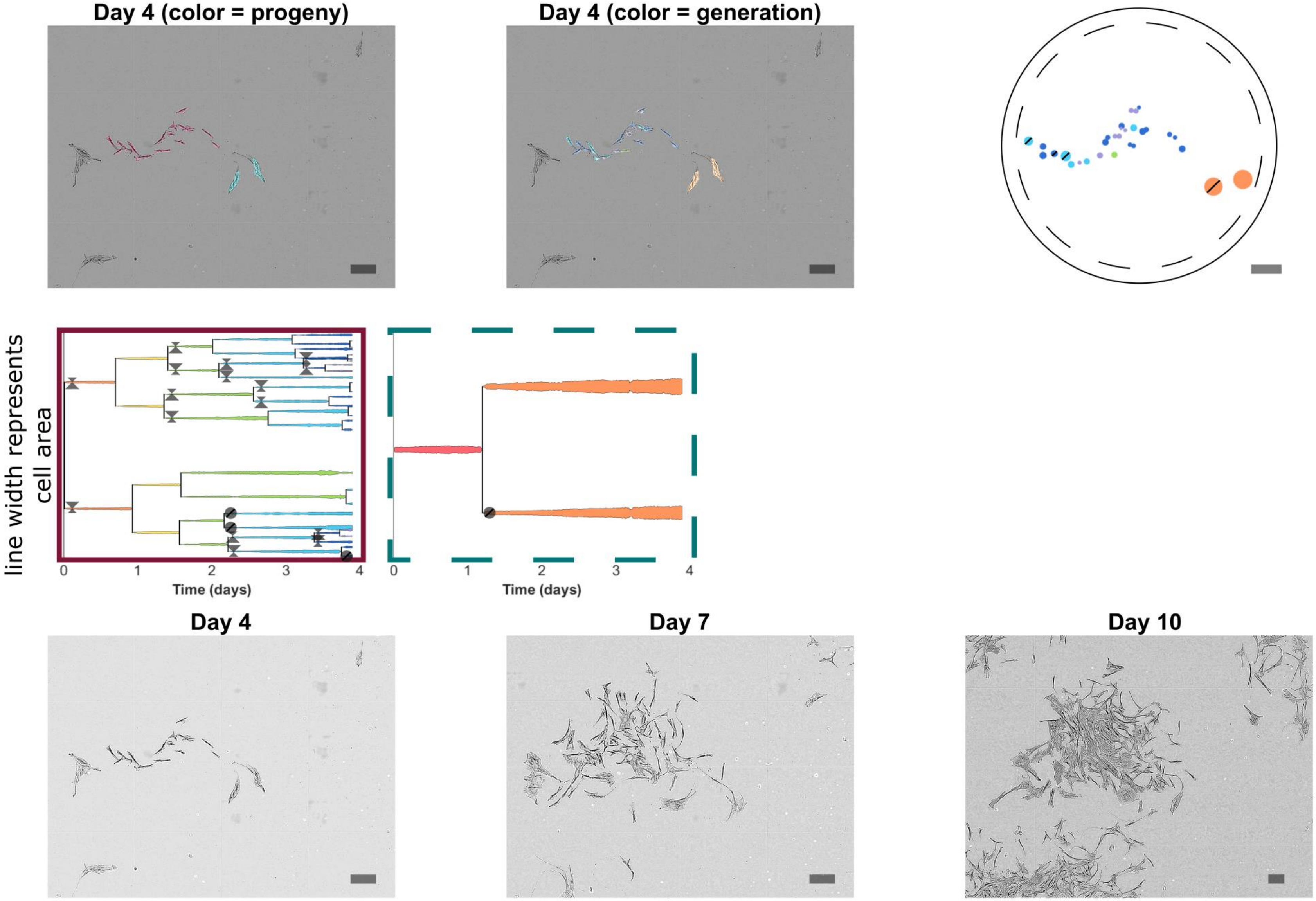
Day 4 (color = progeny)
Day 4 (color = generation)
line width represents cell area
0
1
2
3
4
Time (days)
0
1
2
3
4
Time (days)
Day 4
Day 7
Day 10

Colony V

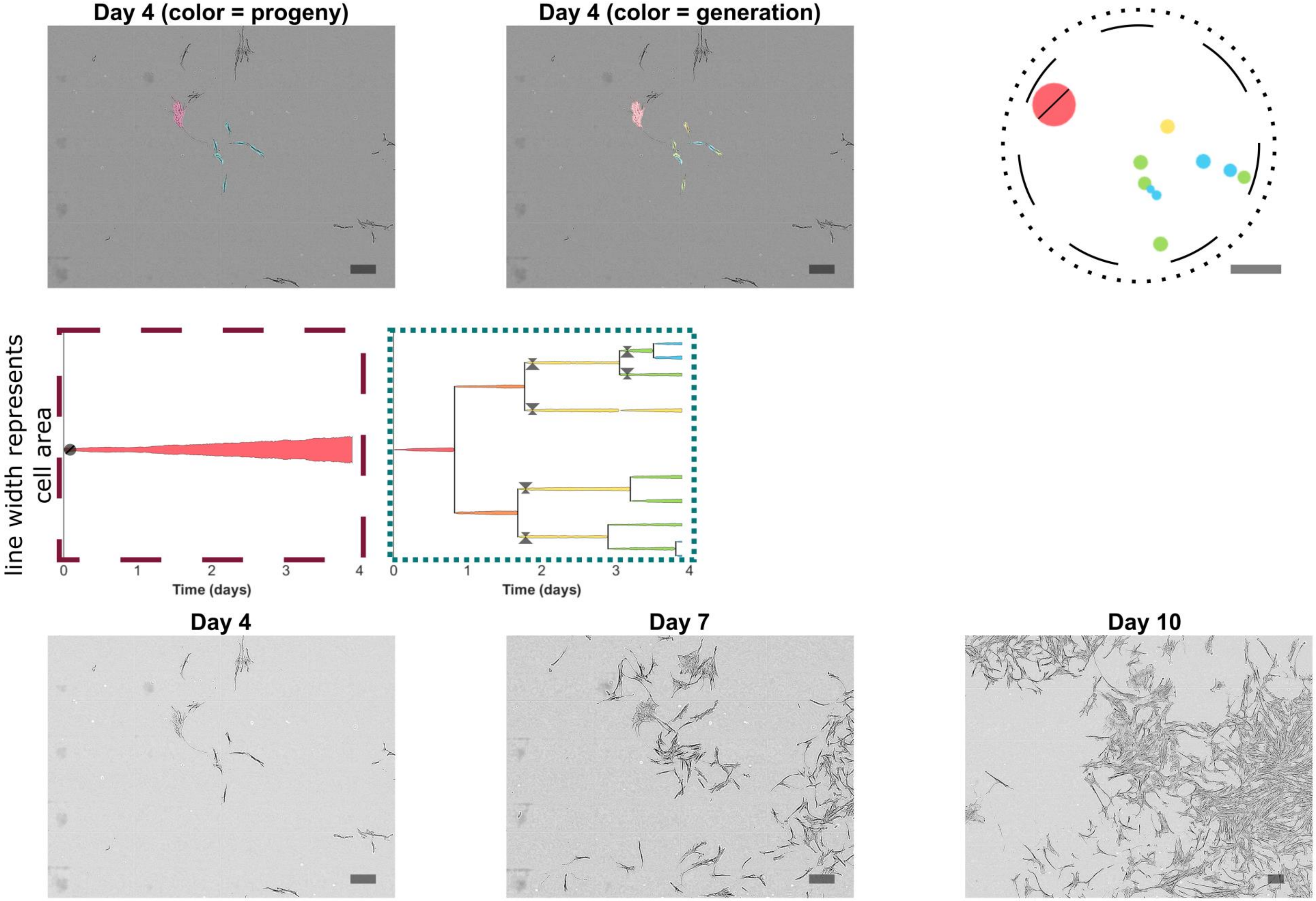

Day 4 (color = progeny)
Day 4 (color = generation)
line width represents cell area
0
1
2
3
4
Time (days)
0
1
2
3
4
Time (days)
Day 4
Day 7
Day 10

Colony W

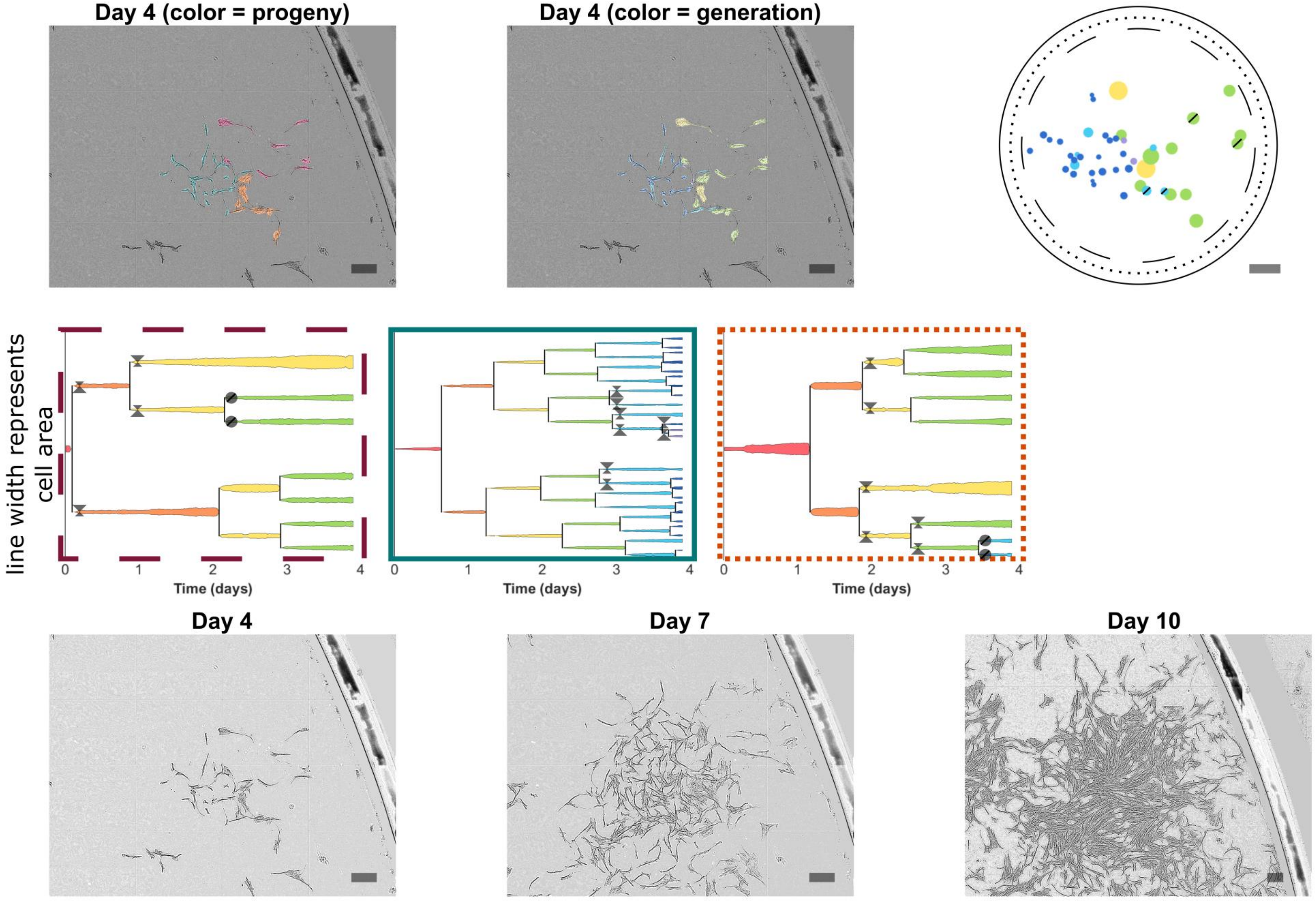
Day 4 (color = progeny)
Day 4 (color = generation)
line width represents cell area
0 1 2 3 4
Time (days)
0 1 2 3 4
Time (days)
0 1 2 3 4
Time (days)
Day 4
Day 7
Day 10

Colony X

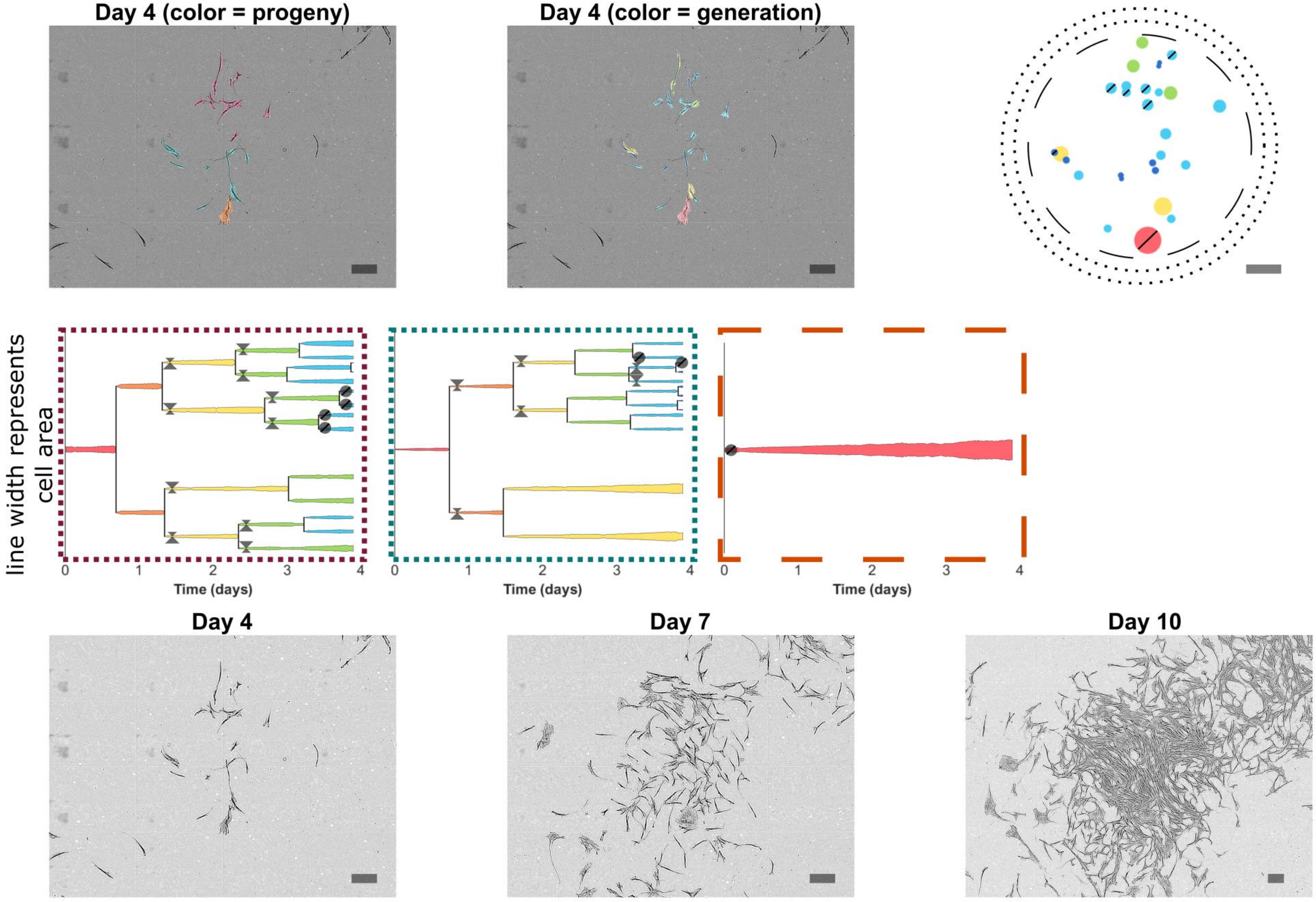

Day 4 (color = progeny)
Day 4 (color = generation)
line width represents cell area
0
1
2
3
4
Time (days)
Day 4
Day 7
Day 10

Colony Y

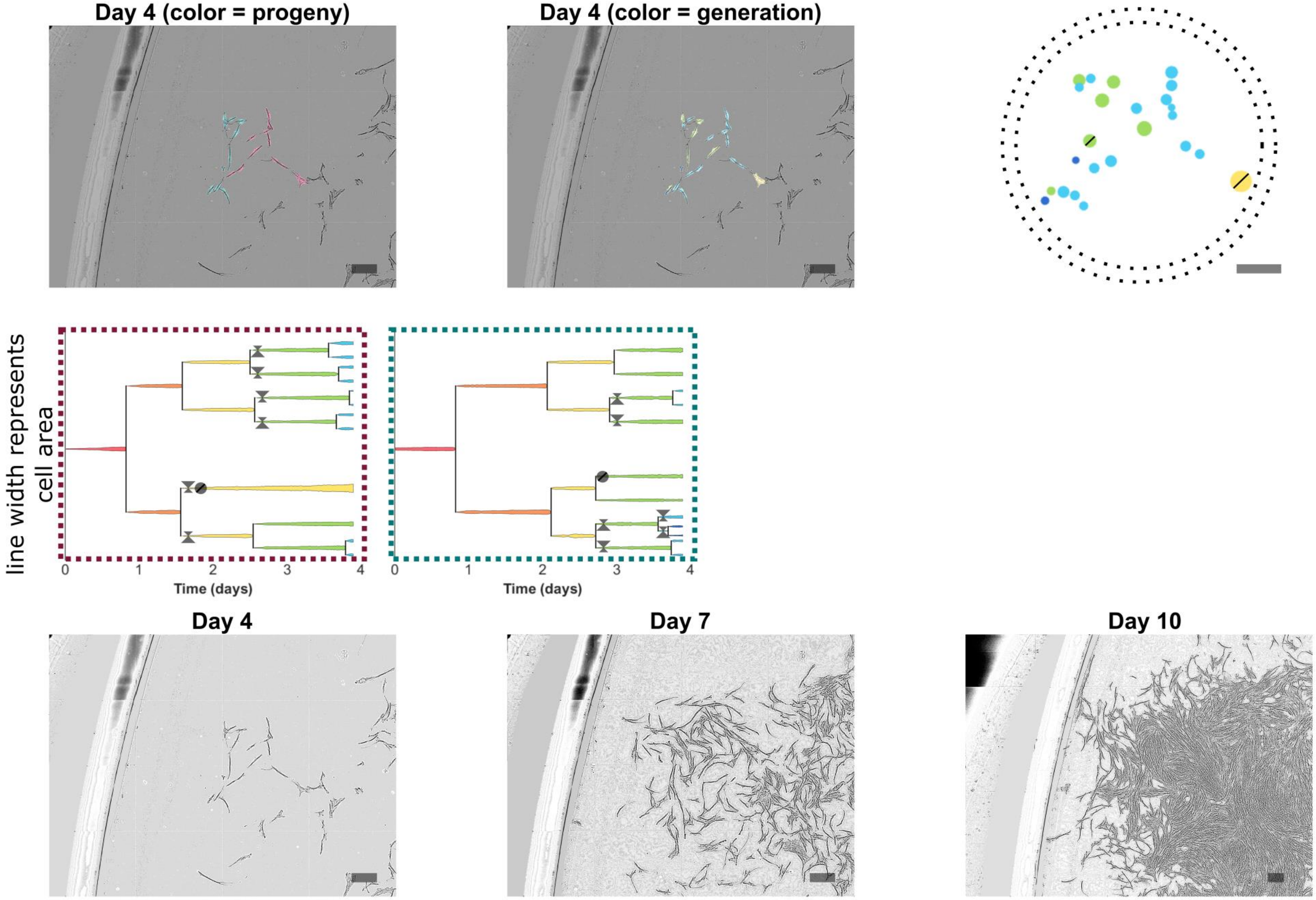

Day 4 (color = progeny)
Day 4 (color = generation)
line width represents cell area
0
1
2
3
4
Time (days)
Day 4
Day 7
Day 10

Colony Z

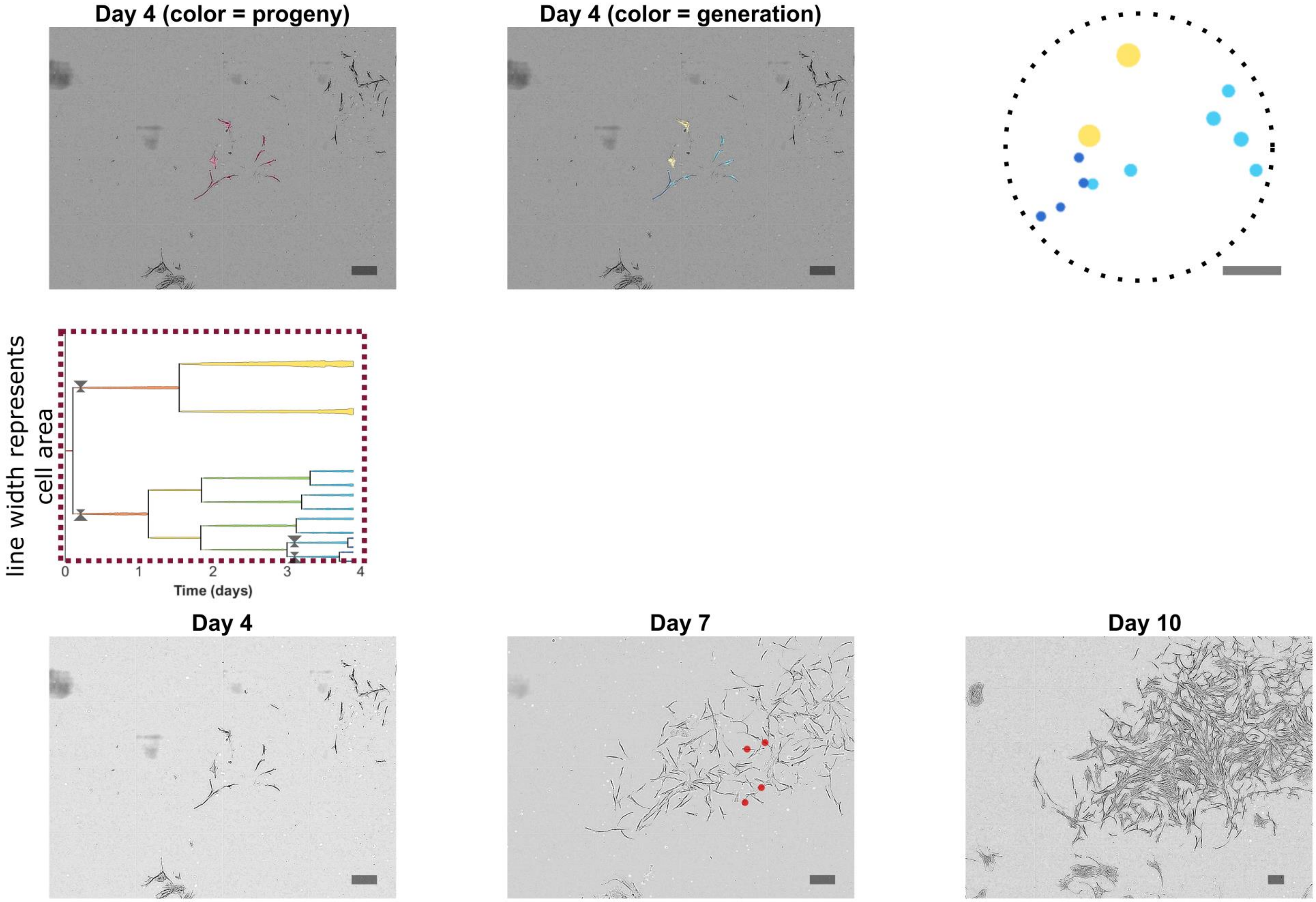

Day 4 (color = progeny)
Day 4 (color = generation)
line width represents cell area
Time (days)
0
1
2
3
4
Day 4
Day 7
Day 10
