# Supplementary figures and images for: Emergent heterogeneity in putative mesenchymal stem cell colonies: Single-cell time lapsed analysis
Source: PLoS One. 2019 Apr 3;14(4):e0213452. doi: 10.1371/journal.pone.0213452 (PMC6447157; doi:10.1371/journal.pone.0213452)

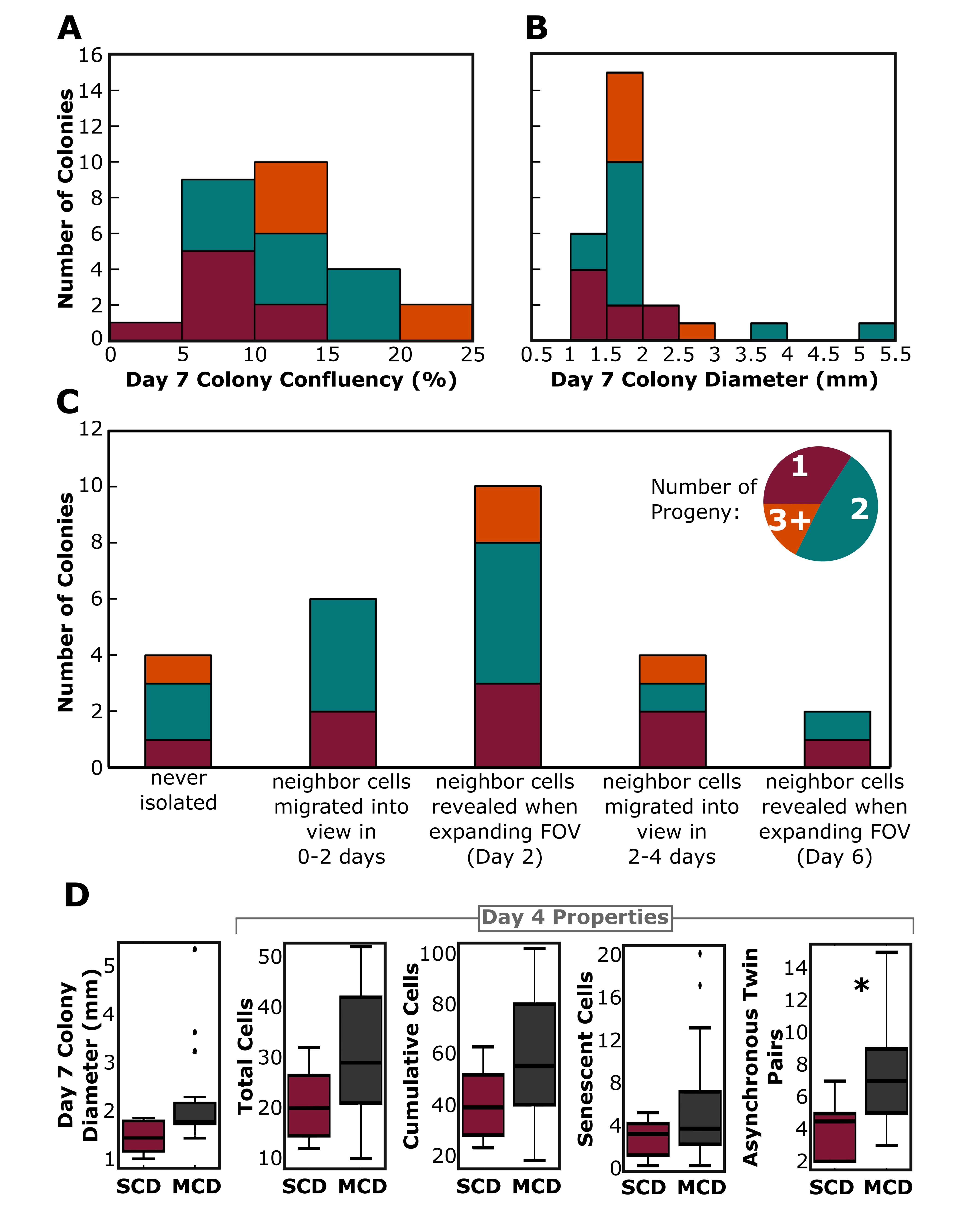

Supplement: S2 Fig — (A) The confluency of colonies at Day 7, organized by number of originating progenies studied at the single-cell level up to Day 4. In general, single-cell-derived (SCD) colonies tended to be lower in confluency relative to colonies originating from two or more cells, though there was no statistical correlation between colony confluency and number of cells it originated from. (B) The approximate diameter of all colonies studied at Day 7 is reported; again, no statistical correlation was found between colony diameter and the number of originating progenies. (C) A categorical analysis of the degree of isolation the colonies developed in is presented. Four colonies developed from originating cells that attached relatively close to neighboring cells. In these cases, the initial 1.7 x 1.3 mm montaged field of view (FOV) contained one or more cells close enough to the studied progeny/progenies to be observed at the first time point, yet far enough away that they and their progeny migrated into and out of view and therefore could not be analyzed at the single-cell level. In the next category, neighboring colonies migrated into the FOV of the developing colony within the first two days of development. Many of the colonies were classified into the middle category: no neighboring cells were observed until the FOV was expanded to 2.6 x 2.1 mm at the end of Day 2 (see Methods). In the fourth category, cells not belonging to the originating progeny migrated into the expanded FOV between Days 2 and 4. In the final category, the neighboring cells closest to the developing colony were not revealed until the FOV was again expanded (3.5 x 2.6 mm). (D) Boxplots reporting pairwise comparisons of single-cell-derived (SCD) versus multi-cell-derived (MCD) colonies of several properties using a Student’s t-test (see S1 Table for all properties analyzed). Ovals outside of whiskers denote statistical outliers. All measured colony properties differing between MCD and SCD colonies at the p < 0 [file pone.0213452.s002.tif]

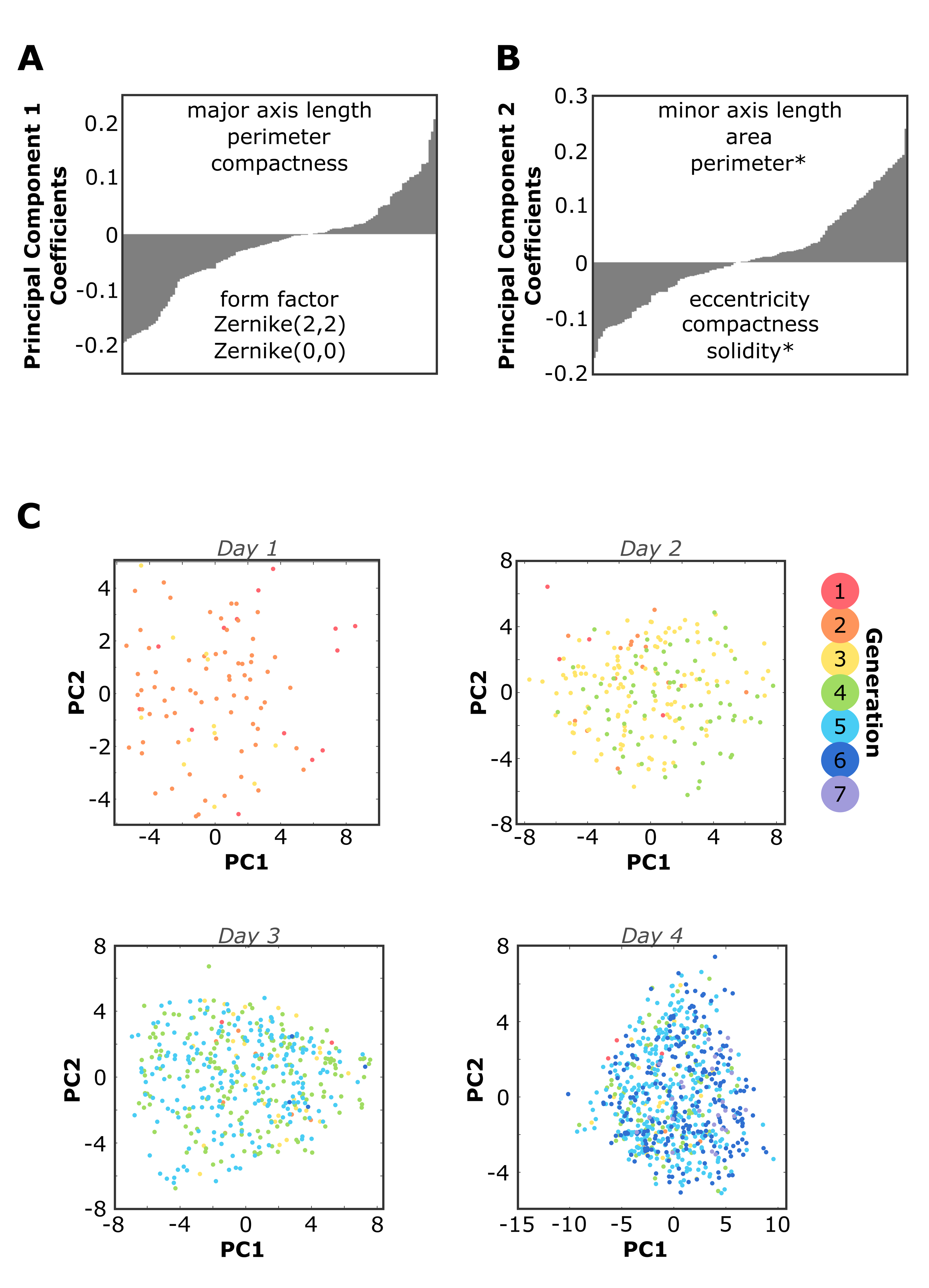

Supplement: S3 Fig — (A) Coefficient values of principal component 1 (PC1) for the properties analyzed in the PCA presented in Fig 3B. The observed trend in PC1-PC2 space in Fig 3 was not caused by a few properties, but rather a linear combination of many. Properties with the highest coefficient values in positive and negative PC1 space are listed and represent the average measurements over the course of the cells’ lifetimes (e.g., the average value for major axis length of cells over their lifetimes had the highest positive coefficient value for PC1). (B) Similarly, the coefficients for PC2 are presented for the PCA in Fig 3B. The top-contributing properties represent the average measurements over the course of the cells’ lifetimes, with the exception of those marked with an asterisk (*), which were properties measured at the time point of each cell’s birth (e.g., the solidity of cells at the time point of their birth had the third-largest contribution in negative PC2 space). (C) PCA was conducted on all cells existing at the indicated time points, where the input measurements were properties measured directly by CellProfiler at that time point in the experiment (rather than at time points relative to the cells’ timeline, as in Fig 3). The trend in generation is not apparent in this method of analysis, demonstrating that the biophysical properties of cells, while fundamentally different among cell generations over the course of their lifetimes, cannot indicate the generation or function of cells at any one given time point. (TIF) [file pone.0213452.s003.tif]

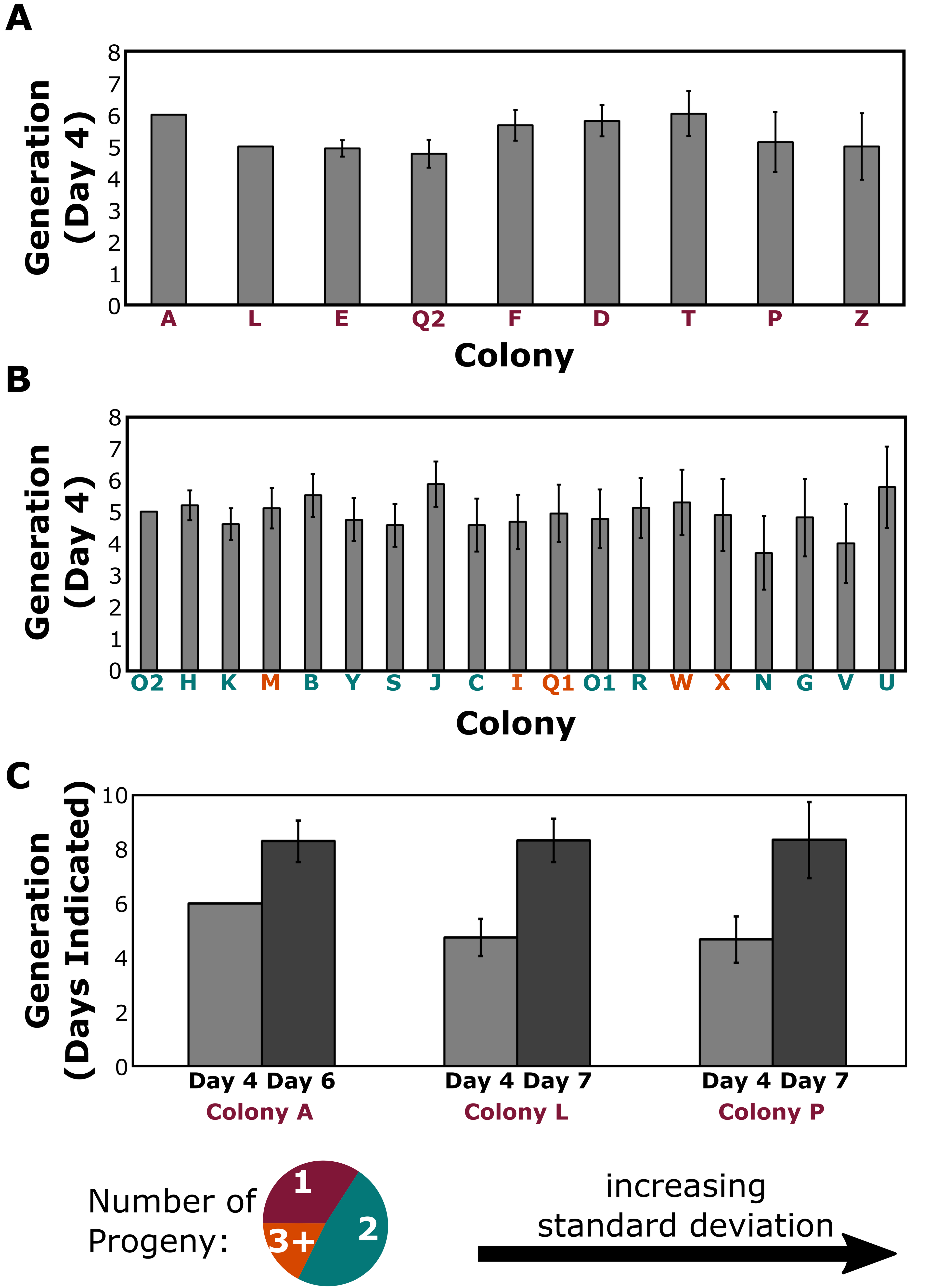

Supplement: S4 Fig — (A) Average generation of cells for each single-cell-derived (SCD) colony. Error bars represent one standard deviation. (B) Average generation of cells for each multi-cell-derived (MCD) colony at Day 4. The number of originating progenies is indicated by color. MCD colonies had statistically higher standard deviations in generation at Day 4 compared to SCD colonies in a t-test (p < 0.001). (C) The average cell generation for three SCD colonies analyzed up to Day 6 or 7. The range of generations for each colony increased after Day 4 in all three colonies. (TIF) [file pone.0213452.s004.tif]

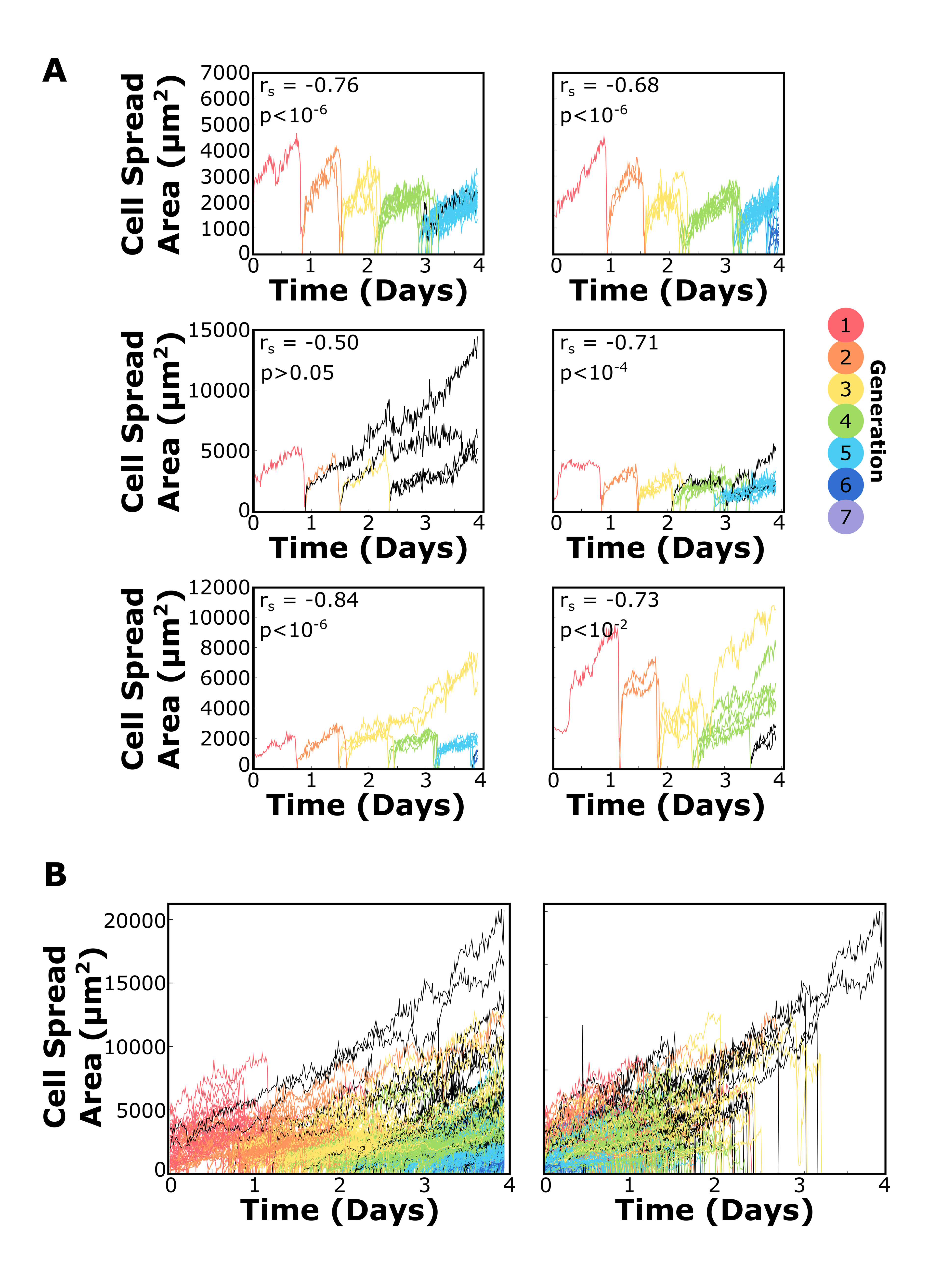

Supplement: S5 Fig — (A) Further examples of area-vs-time curves for individual cells are given for several progenies to demonstrate the relationship between cell area, generation (color scheme provided), and senescence (labeled black). The Spearman correlation coefficient (rs) and associated p- value for the comparison of average cell area versus generation is also given for each progeny. The top two graphs represent two progenies with a strong decreasing trend in average cell area and generation. The middle two graphs show examples of cells within single progenies that deviate from this trend due to senescence, and the bottom two graphs demonstrate that not all deviating cells were senescent. (B) Area vs time for all cells studied, with time labeled relative to the start of the experiment (left) and relative to the birth time point of each cell (right). While a few senescent cells grew to a very large spread area, area alone could not distinguish senescent from proliferating cells. (TIF) [file pone.0213452.s005.tif]

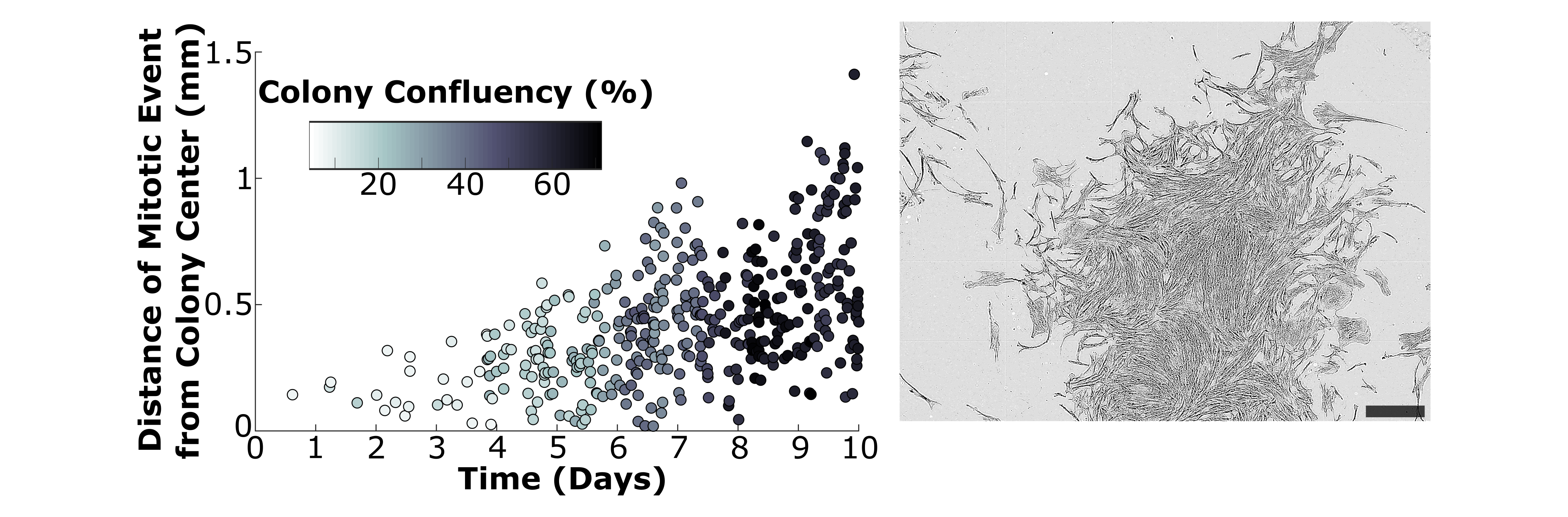

Supplement: S6 Fig — The location of each cell division was measured over the course of ten days of colony development and is reported as the location relative to the colony center (left). Colony confluency was also measured at each time point (indicated in the color bar), and a phase contrast image of the colony at Day 10 qualitatively demonstrates the confluent conditions in which mitotic events were detected (right; adjusted for brightness and contrast). Scale bar = 0.5 mm. (TIF) [file pone.0213452.s006.tif]

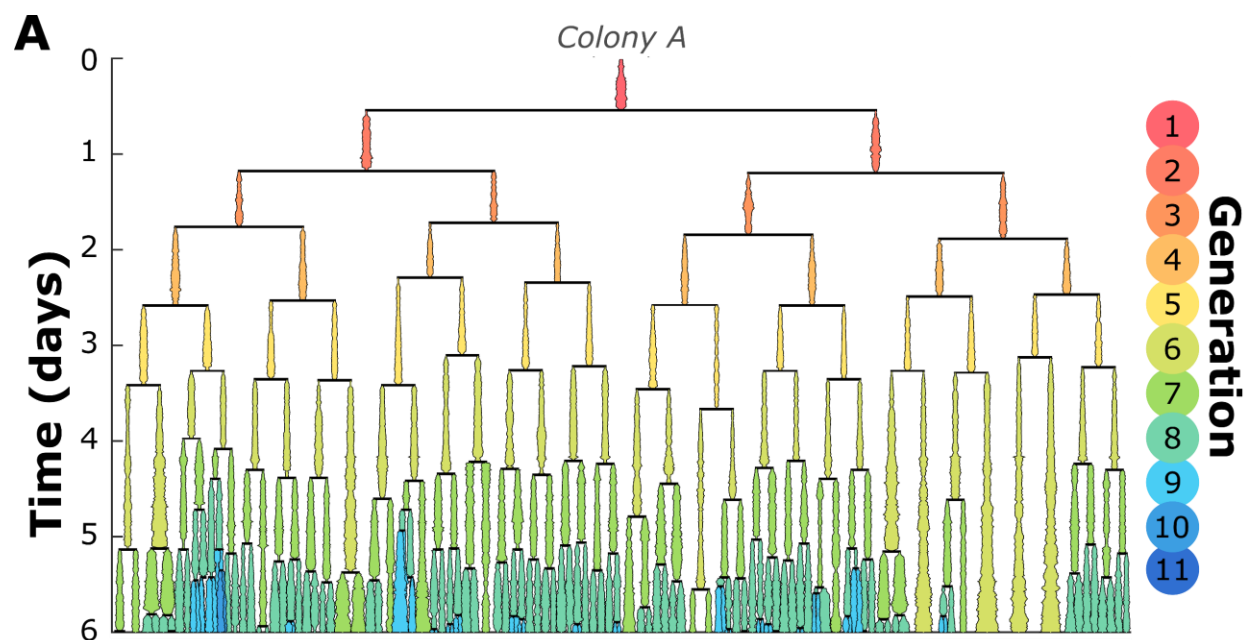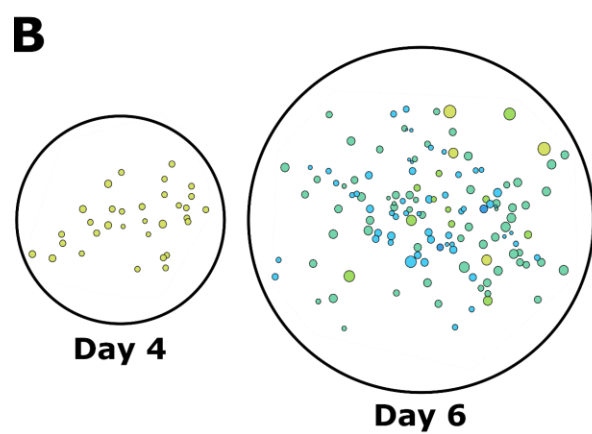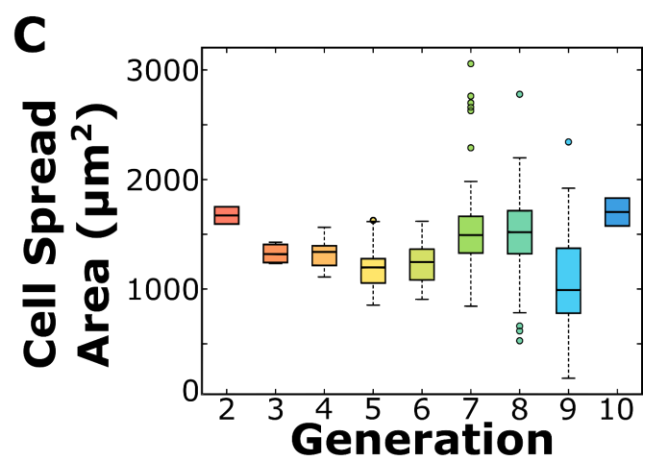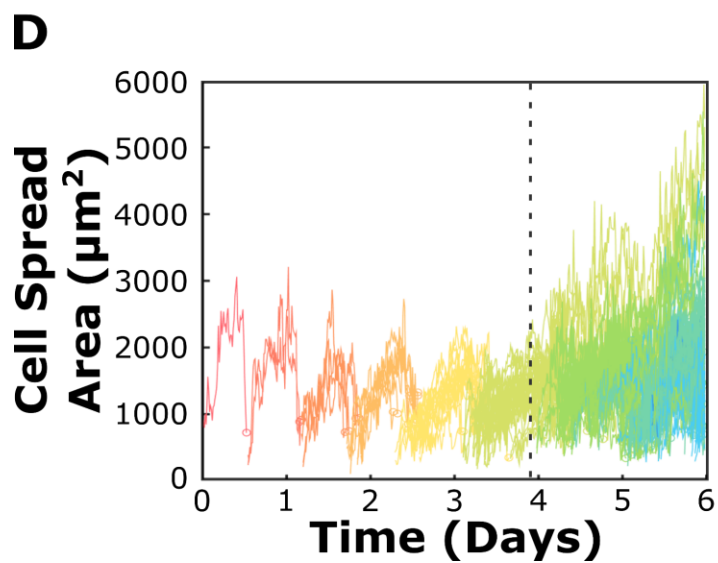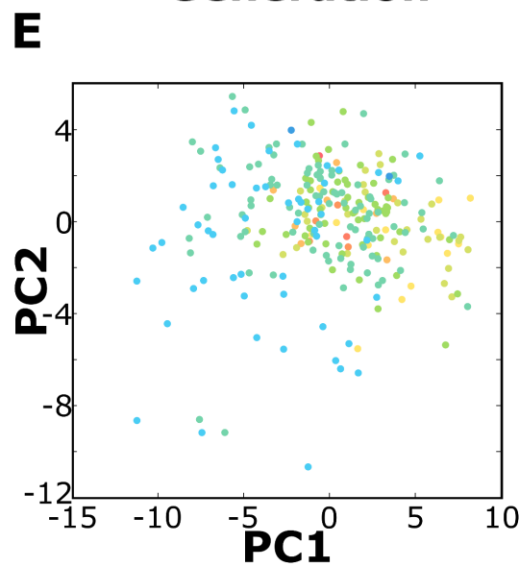

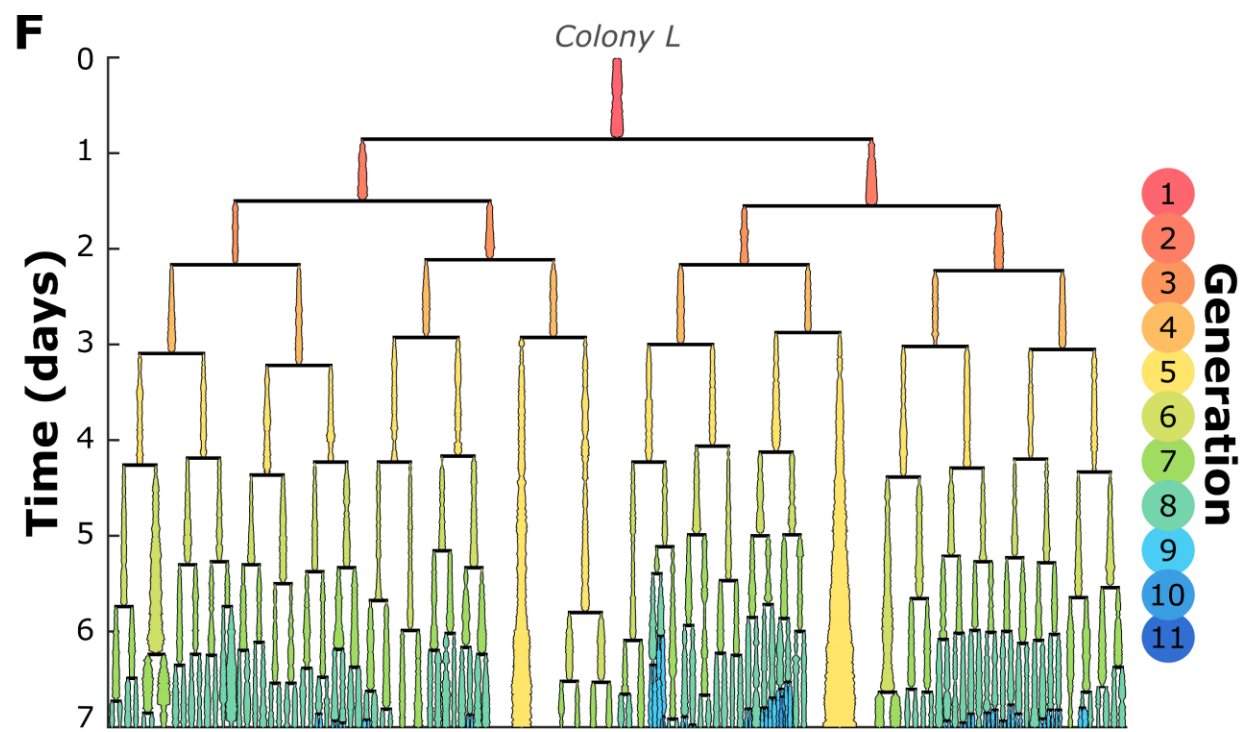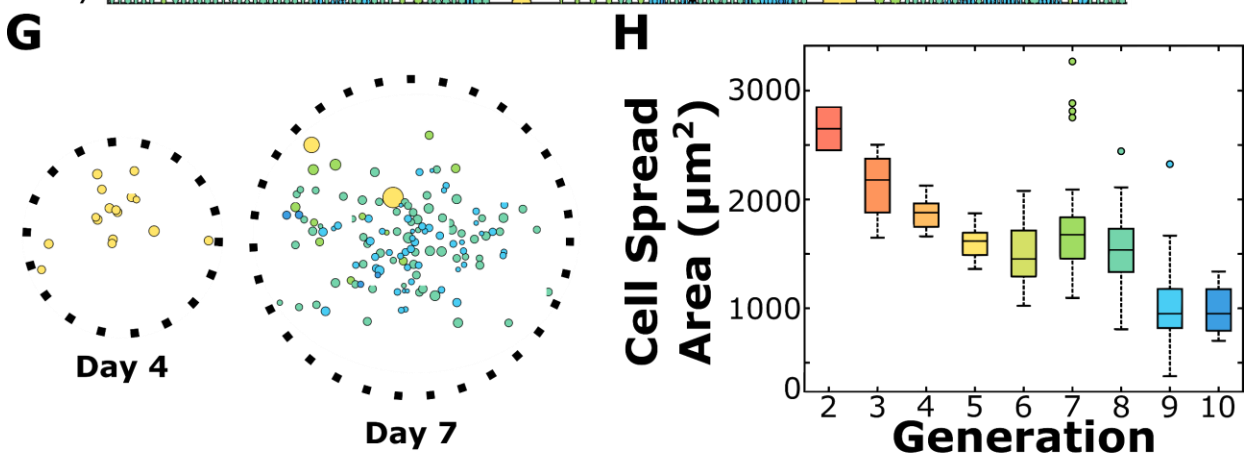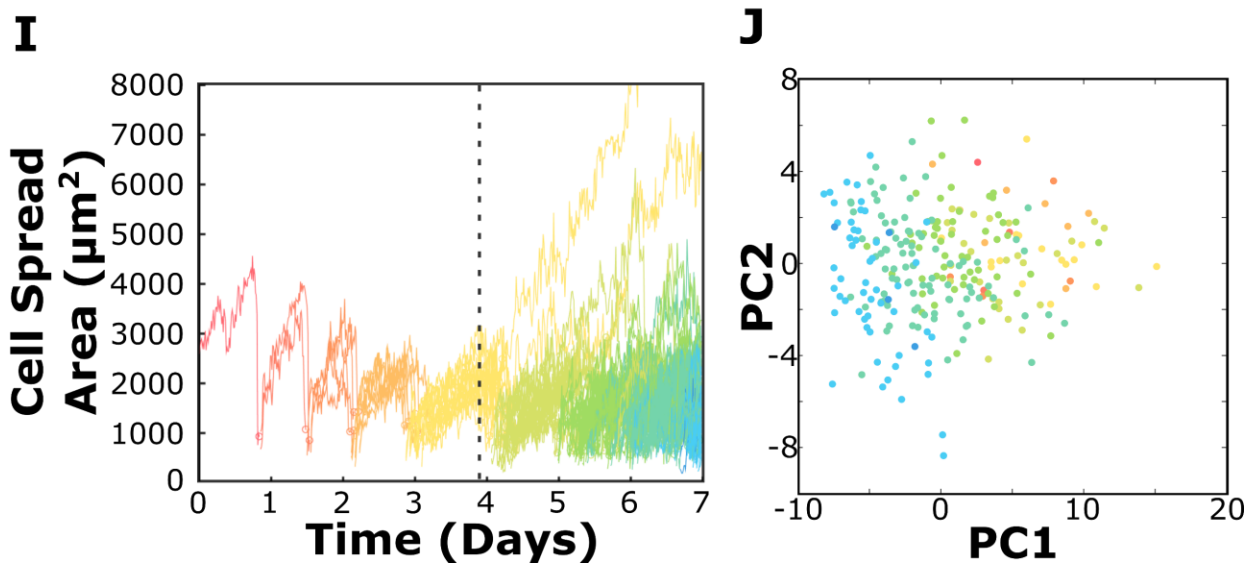

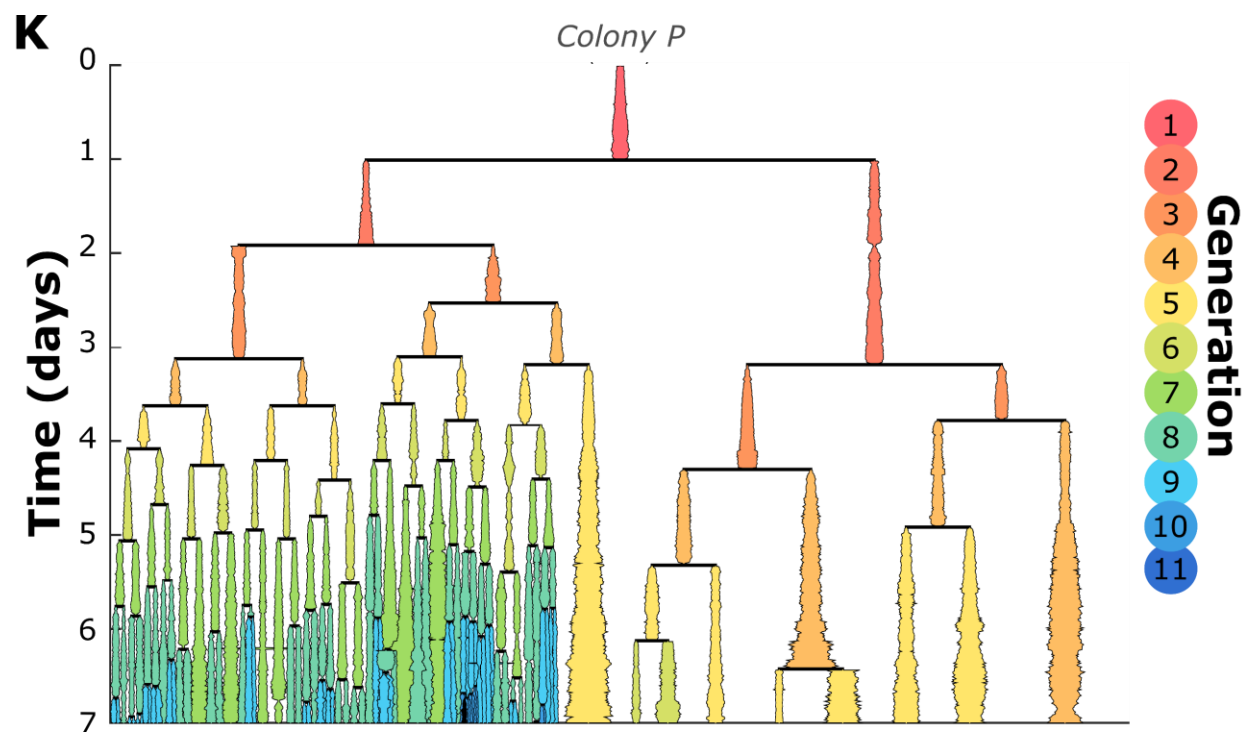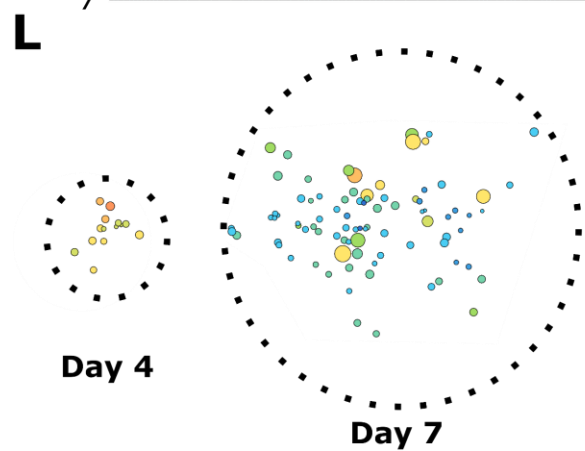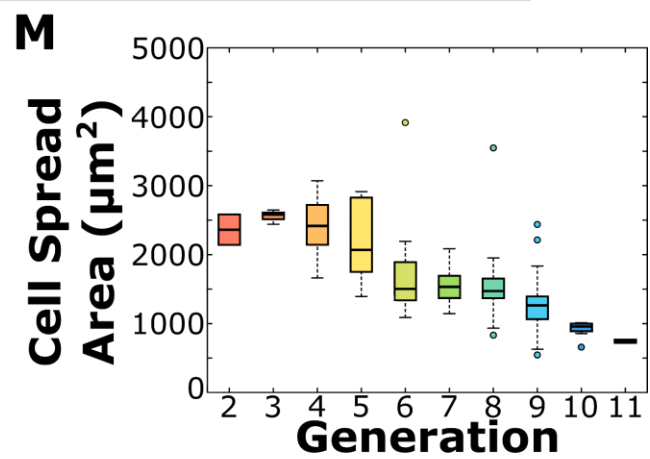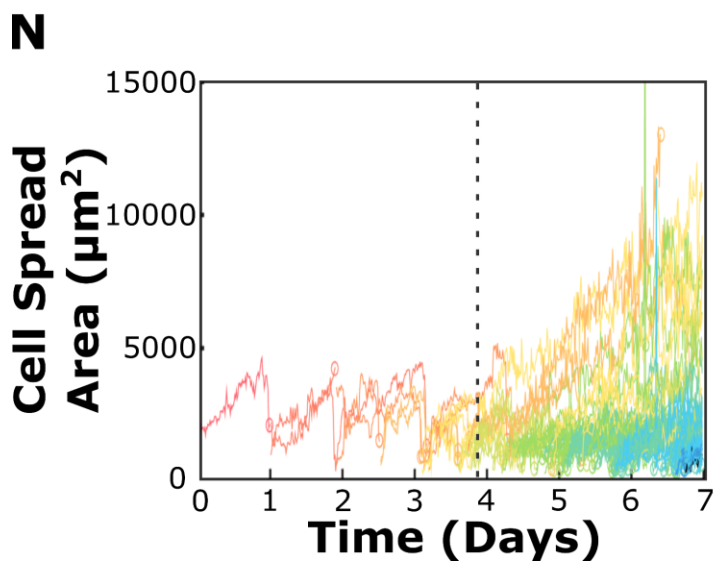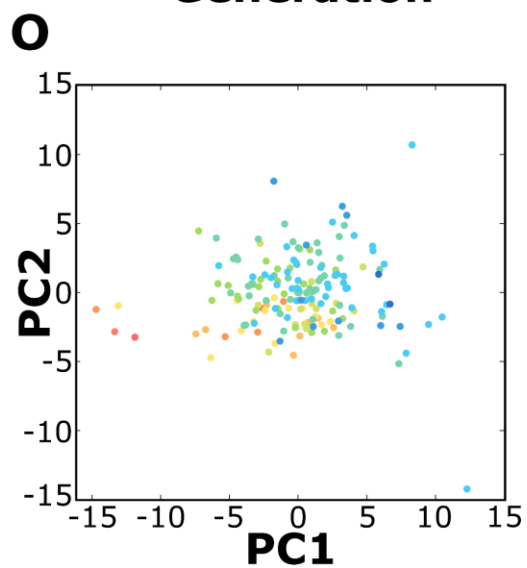

Supplement: S7 Fig — Three single-cell-derived (SCD) colonies (Colonies A, L, and P) were analyzed at the single-cell level up to Day 6 or Day 7. (A, F, K): Lineage trees for each SCD colony. Lineage lines are color-coded by cell generation, and the width of the lines represents the area of the cell at each 15-minute time point. (B, G, L): Glyphs representing cell area, generation, location, and proliferative capacity of their progeny at fixed time points (see Fig 4 for detailed key). (C, H, M): Boxplots of cell area, categorized by cell generation. Circles outside of whiskers represent statistical outliers. Cell area was calculated as the average area of each cell over all 15-minute time points for the first 0.83 days of their lifetimes. One outlier data point in panel M (5th generation, average area = 7,288 μm2) was removed from this figure for scaling purposes. (D, I, N): Area-vs-time curves for each cell over the course of analysis. Data line color represents cell generation, and the vertical dashed line indicates the time point at which cells were fed. (E, J, O): Principal component analysis (PCA) of all cells belonging to the colony over the course of analysis. Input variables were averaged values of 54 properties measured by CellProfiler (see Methods), and data point colors represent cell generation. (PDF) [file pone.0213452.s007.pdf]
